# WC1/WC2–Sre1 Transcriptional Cascade Controls Predation and Chlamydospore Formation in the Nematode‐Trapping Fungus *Arthrobotrys flagrans*

**DOI:** 10.1111/1751-7915.70412

**Published:** 2026-07-09

**Authors:** Yu Zhang, Jiafang Zuo, Qianfei Shi, Peiji Zhao, Hanbo Zhang, Minghe Mo, Guohong Li

**Affiliations:** ^1^ State Key Laboratory for Conservation and Utilization of Bio‐Resources in Yunnan, Yunnan Key Laboratory of Basic Research and Innovative Application for Green Biological Production Yunnan University Kunming China; ^2^ School of Ecology and Environment Yunnan University Kunming China

**Keywords:** *Arthrobotrys flagrans*, biocontrol, chlamydospores, GATA‐type transcription factor, pathogenicity, plant‐parasitic nematodes

## Abstract

*Arthrobotrys flagrans* is a nematode‐trapping fungus that kills plant‐parasitic nematodes (PPNs) using adhesive traps and withstands adverse conditions by producing chlamydospores. It holds promise as a biocontrol agent against PPNs, yet the mechanisms underlying its pathogenicity and chlamydospore formation remain poorly characterized, hampering the development of efficient biocontrol strains. Here, we systematically characterized all eight GATA‐type transcription factors in *A. flagrans* and found that all were significantly upregulated during both trap and chlamydospore formation. These eight regulators exhibit specialized and coordinated functions in hyphal growth, stress responses, pathogenicity, and chlamydospore formation. WC2, WC1, Sre1, AreA, ASD4 and GATA1 positively regulate pathogenicity, whereas Ams2 and NsdD act as negative regulators, likely through the repression of virulence factors and adhesins. Moreover, all eight factors govern chlamydospore number, with WC1, WC2 and Sre1 additionally controlling chlamydospore diameter. Further investigation of their interactions and regulatory relationships revealed a novel, light‐independent WC1/WC2–Sre1 transcriptional cascade as a key node within the network governing pathogenicity and chlamydospore formation. Overexpression of *WC2* significantly increases pathogenicity and chlamydospore production, providing functional validation that WC2 is a precise genetic target for engineering superior biocontrol strains. Overall, this work elucidates the functional atlas of GATA‐type transcription factors in *A. flagrans* , reveals a novel light‐signalling co‐option mechanism for developmental regulation, and provides both actionable molecular targets for strain improvement and a theoretical framework for advancing the biocontrol of PPNs.

## Graphical abstract

Systematic functional characterization of all eight GATA‐type transcription factors in the nematode‐trapping fungus *Arthrobotrys flagrans* reveals a novel, light‐independent WC1/WC2–Sre1 transcriptional cascade that orchestrates both trap formation and chlamydospore development, providing actionable targets for engineering superior biocontrol strains.

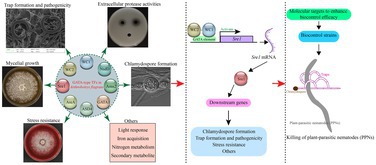

## 1 Introduction

Plant‐parasitic nematodes (PPNs) cause billions of dollars in annual crop losses, threatening global food security (Jones et al. 2013; Huang et al. 2025; Vanin et al. 2025). The unsustainability of chemical nematicides has spurred the search for eco‐friendly alternatives, with nematophagous fungi emerging as promising biocontrol agents (Li et al. 2015; Afzal and Mukhtar 2024). As natural antagonists of nematodes, nematophagous fungi represent an important resource for nematode control and offer a promising alternative strategy for sustainable management (Li et al. 2015, 2024). Of these fungi, nematode‐trapping (NT) fungi have emerged as promising biocontrol agents, valued for their minimal toxicity, remarkable efficacy, and environmental compatibility, garnering increasing attention in recent years (Wernet and Fischer 2023; Zhang et al. 2023; Hu et al. 2024).

*Arthrobotrys flagrans* (formerly *Duddingtonia flagrans*) is a model NT fungus that captures nematodes using adhesive traps and survives adverse conditions as chlamydospores (Wernet and Fischer 2023; Zhang et al. 2025). The chlamydospore‐based agent Bioverm has been applied to control parasitic nematodes in various animals (Bastos Ferreira et al. 2026). Moreover, *A. flagrans* has been demonstrated to exhibit nematicidal activity against plant‐parasitic nematodes such as *Meloidogyne incognita*, *M. javanica* , and *Xiphinema index*, positioning it as a primary source for the development of bionematicides (Mei et al. 2021; Wernet and Fischer 2023). Biocontrol agents based on their chlamydospores offer enhanced storage stability under various conditions and a longer shelf life compared with conidia‐based agents, highlighting their greater potential for practical application (Peng et al. 2021).

As an important indicator of transition from a saprophytic to a predatory lifestyle in NT fungi, trap formation has become a major focus of research seeking to elucidate its underlying regulatory mechanisms. Previous studies have shown that G‐protein‐coupled receptor GprC, polyketide synthase, CFEM‐domain proteins, virulence factors (NipA, TrsA, CyrA) (Wernet et al. 2021; Emser et al. 2024, 2025), cell‐end marker proteins (TeaA) (Kriegler et al. 2025), and intercellular communication proteins (SofT) (Youssar et al. 2019) all participate in trap formation and are critical for pathogenicity in *A. flagrans* . The STRIPAK (striatin‐interacting phosphatase and kinase) component SipC and transcription factors FlbD and Swi6 contribute significantly to both trap and chlamydospore development in *A. flagrans* (Wernet et al. 2022; Zhang et al. 2023, 2025; Linghu et al. 2024). Notably, the velvet family proteins and LaeA are conserved regulators of fungal pathogenesis across diverse genera, including the NT fungi *Arthrobotrys oligospora* and *A. flagrans* , where they govern trap formation and nematode virulence (Zhang et al. 2023; Calvo et al. 2024). More recently, iron acquisition mediated by siderophores has been shown to be essential for adhesive knob formation and pathogenicity in the NT fungus *Dactylellina haptotyla*, adding a new dimension to our understanding of the nutritional and metabolic requirements for trap morphogenesis (Chen et al. 2023; Miao et al. 2023; Lei et al. 2025, 2026). Furthermore, research on the mechanisms of chlamydospore formation has largely focused on the unicellular fungus *Candida albicans* , with comparatively fewer studies in filamentous fungi (Hernández‐Cervantes et al. 2020). Due to the complexity of trap and chlamydospore formation, the transcriptional regulatory network governing these processes remains poorly understood, and identifying additional regulatory factors is required to elucidate the underlying mechanisms.

Transcription factors are regulatory proteins capable of binding DNA, modulating the transcriptional process of genes, and thus play a key role in gene expression regulation (Moon et al. 2025). GATA‐type transcription factors are evolutionarily highly conserved and present in animals, fungi, and plants (Virolainen and Chekunova 2024). These factors contain a characteristic zinc finger (ZnF) DNA‐binding domain composed of a specific arrangement of amino acids (CX_2_CX_17−20_CX_2_C), which folds into two β‐strands followed by an α‐helix (Schwechheimer et al. 2022; Virolainen and Chekunova 2024). Four zinc‐coordinating cysteines enable the recognition of the consensus sequence (A/T)GATA(A/G) in target promoter regions (Moon et al. 2025). GATA‐type transcription factors are encoded across diverse eukaryotes, with the largest repertoires found in plants (e.g., 28 in *Brachypodium distachyon* , 39 in *Populus trichocarpa* , and 87 in *Gossypium hirsutum* ), whereas other lineages possess more limited sets, such as six in humans, five in *Drosophila melanogaster* , ten in *Saccharomyces cerevisiae* , and seven in *Aspergillus oryzae* (Schwechheimer et al. 2022; Virolainen and Chekunova 2024). Although the GATA‐type transcription factor family has been identified in diverse organisms, only a limited number of members in fungi have been functionally characterized to date (Ren et al. 2024). These include WC1 (also known as WCL1, WC‐1, or LreA), WC2 (WCL2/WC‐2), AreA (Nrf1, ClnR1, Fnr1, Nut1, Nit2), NsdD, ASD4 (AreB), Ams2, and Sre1 (SreA, Urbs1) (Schwechheimer et al. 2022).

WC1 and WC2 were identified as central components in mediating fungal blue‐light perception and the control of circadian rhythms (Froehlich et al. 2002). Beyond their roles in light responses, the WC complex also governs mushroom development, secondary metabolism, and virulence (Vonk et al. 2024). NsdD acts as a positive regulator of sexual development and a repressor of asexual reproduction in *Aspergillus* species (Moon et al. 2025). Furthermore, it plays a critical role in the biosynthesis of secondary metabolites, including melanin, pigments, and sterigmatocystin (Song et al. 2025). AreA and ASD4 are central transcriptional regulators of nitrogen metabolism. They also modulate broader biological processes such as hyphal growth, conidiation, and secondary metabolism (Li et al. 2026). Sre1 is a master regulator of iron homeostasis in fungi, primarily repressing iron acquisition genes under iron‐replete conditions to prevent toxic accumulation (Chao et al. 2008). Ams2 controls histone gene expression and chromosome segregation, processes essential for growth, appressoria development, and virulence (Trickey et al. 2013; Liu et al. 2018). In summary, GATA‐type transcription factors are pivotal regulators with pleiotropic functions that are vital for numerous aspects of biological processes (Virolainen and Chekunova 2024). However, the diversity and functions of GATA‐type transcription factors in NT fungi remain unknown.

In this study, we systematically identified all eight GATA‐type transcription factors in *A. flagrans* and found that each was significantly upregulated during both chlamydospore and trap formation. To dissect how these factors orchestrate the dual life strategy of predation and sporulation, we combined targeted gene deletions with multi‐phenotypic profiling to define their roles in growth, stress adaptation, pathogenicity, and chlamydospore formation. We further characterized the protein interaction network among these eight factors and constructed the regulatory network of WC2 via integrated RNA‐seq, DAP‐seq, and ChIP‐qPCR analyses. Focusing on the WC1/WC2 complex, we demonstrated its central role and uncovered a downstream cascade in which it directly activates another GATA‐type transcription factor, Sre1, to jointly regulate chlamydospore formation and nematode pathogenicity. Together, this systematic dissection not only fills a fundamental knowledge gap in our understanding of the regulatory cascades governing trap and chlamydospore development in NT fungi but also provides a theoretical framework and candidate molecular targets for enhancing biocontrol efficacy against PPNs.

## 2 Materials and Methods

### 2.1 Strains and Culture Conditions

The wild‐type (WT) strain *A. flagrans* was cultured on PDA at 28°C. All knockout and transgenic strains were maintained on PDA containing appropriate selective antibiotics (hygromycin B, G418, or both) according to their resistance markers. Detailed strain genotypes, antibiotic concentrations, and sources are provided in Table S1 and the Supporting Information.

### 2.2 Sequence and Phylogenetic Analysis

Gene structure, conserved domains, and phylogenetic relationships were analysed using standard bioinformatic tools as detailed in the Supporting Information.

### 2.3 Protoplast Preparation and Transformation

Protoplast isolation and PEG‐mediated transformation of *A. flagrans* were performed exactly as described in our previous study (Zhang et al. 2025). The detailed composition of buffers, enzymatic digestion conditions, and regeneration medium is provided in the Supporting Information. Positive transformants were selected on PDA containing hygromycin B (100 μg/mL) or G418 (50 μg/mL).

### 2.4 Gene Deletion and Complementation

Knockout cassettes containing ~1 kb upstream and downstream homologous arms and the hph resistance gene were assembled into pUC19 and transformed into protoplasts (Zhang et al. 2025). Complementation constructs carrying the native promoter, ORF, and terminator of each gene together with a G418 resistance cassette were introduced into the respective deletion mutants. The detailed protocol is provided in the Supporting Information. Primers used for strain construction are listed in Table S2.

### 2.5 GFP Fluorescence Localization

*WC1* and *WC2* were C‐terminally tagged with GFP and expressed under their native promoters (Zhang et al. 2025). Nuclei and cell walls were counterstained with DAPI and Calcofluor White, respectively. Detailed construction procedures are provided in the Supporting Information.

### 2.6 Bimolecular Fluorescence Complementation (BiFC) Assay

The BiFC assay was conducted based on a split‐GFP system (Hu et al. 2024). GFP was split into the N‐terminal (GFP^N^) and the C‐terminal (GFP^C^) fragments. The GFP^N^ was fused to the N‐terminus of WC1, and the GFP^C^ to the C‐terminus of WC2. Both fusion constructs, each with its native regulatory sequences, were co‐transformed into protoplasts. Control strains carrying individual fusion constructs were generated in parallel. Detailed construction procedures are provided in the Supporting Information.

### 2.7 Gene Overexpression

To create an overexpression plasmid of *WC2* under the control of the *H2B* promoter, the *H2B* gene promoter (~2.0 kb), the *WC2* ORF, and the *H2B* terminator (~1.0 kb) were amplified by PCR, using *A. flagrans* genomic DNA as template (Emser et al. 2024). These fragments, together with the hygromycin resistance gene (*hph*), were assembled in order into the pUC19 vector to yield the *OEWC2* plasmid, which was subsequently transformed into *A. flagrans* protoplasts.

### 2.8 Expression Analysis of *Sre1* in Δ*WC1* and Δ*WC2* Mutants

To test whether Sre1 functions downstream of WC1 and WC2 in regulating chlamydospore and trap formation, we expressed *Sre1* in the Δ*WC1* and Δ*WC2* mutants, respectively (Ren et al. 2024). To this end, a complementation plasmid was constructed by assembling the *H2B* promoter (~2.0 kb), the *Sre1* ORF, the *H2B* terminator (~1.0 kb), and a G418 resistance cassette into the pUC19 vector. Subsequently, the *OESre1* plasmid was transformed into protoplasts of both Δ*WC1* and Δ*WC2* mutants.

### 2.9 Analyses of Mycelial Growth

For growth assays, 0.8 cm mycelial plugs were harvested from 4‐day‐old PDA cultures of the WT and knockout strains. The plugs were centrally inoculated onto PDA and TYGA media (with three replicates per strain) and incubated at 28°C. Colony diameters were measured daily over a 5‐day period, and growth trends were analysed with GraphPad Prism 10 (GraphPad Software, Boston, MA, USA) (Zhang et al. 2025).

### 2.10 Chemical Stress Tolerance Assay

For stress assays, mycelial plugs (0.8 cm in diameter) of WT and knockout strains were inoculated onto PDA plates containing specified concentrations of chemical stressors. The tested compounds and concentrations were as follows: sorbitol (0.25, 0.5 and 1 M), NaCl (0.1, 0.2 and 0.3 M), SDS (0.01%, 0.02% and 0.03%), Congo red (25, 50 and 100 mM), and H_2_O_2_ (2.5 and 5 mM). Inoculated plates were incubated at 28°C for 5 days. Colony diameters were measured daily, and the relative growth inhibition (RGI) was calculated for each strain (Zhang et al. 2023, 2025). Three replicates were performed for each treatment condition.

### 2.11 Chlamydospore Formation Assay

To quantify chlamydospore formation, mycelial plugs (0.8 cm in diameter) were inoculated onto 6‐cm WA plates and incubated at 28°C (Zhang et al. 2025). The plates were monitored daily, and chlamydospore counts were recorded on day 14. Chlamydospore morphology was examined using confocal microscopy, and chlamydospore diameters were measured with Adobe Photoshop (Adobe Systems Incorporated, San Jose, CA, USA). The experiment was independently repeated at least three times, with three biological replicates included for each fungal strain per experiment.

### 2.12 Trap Formation and Pathogenicity Assays

Following a previously described method, fresh mycelia grown in PDB for 1 day were harvested and evenly spread (10 mg) on 3.5 cm water agar (WA) plates. The plates were incubated at 28°C for 2–3 days until the mycelia fully covered the surface. Subsequently, approximately 300 synchronized *C. elegans* were added to each plate to induce trap formation at 28°C. The trap numbers and nematode mortality were observed and recorded at 0, 3, 6, 12, 24, 36 and 48 h post‐inoculation (Zhang et al. 2023, 2025). Three biological replicates were performed for each fungal strain. Per‐trap killing efficiency was calculated as the ratio of nematode mortality (%) to the number of traps for each replicate at 12 and 24 h post‐nematode addition.

### 2.13 Assay of Proteolytic Activity

Strains were cultured in LMZ liquid medium at 28°C with shaking (180 rpm) for 7 days. Proteolytic activity in the fermentation supernatant was measured using the skim milk plate method (Zhang et al. 2023).

### 2.14 Interaction Network Construction

The STRING online platform was used to predict interactions among the eight GATA‐type transcription factors. Selected interactions were validated by yeast two‐hybrid (Y2H) assays using the Y2HGold‐GAL4 system. Coding sequences of *WC2* and *AreA* were cloned into pGADT7 (prey), and *WC1*, *ASD4* and *Sre1* were cloned into pGBKT7 (bait). Co‐transformed Y2HGold strains were assayed on selective media as previously described (Zhang et al. 2023).

### 2.15 Yeast One‐Hybrid (Y1H) Assay

The Sre1 promoter (~2.0 kb) was cloned into pHis2 vector to generate pHis2‐*Sre1* plasmid, and the coding sequence (CDS) of *WC2* was inserted into the pGADT7‐Rec2 vector to create pGADT7‐Rec2‐*WC2* plasmid. Both constructs were co‐transformed into Y187 cells. Interactions were assessed on SD/–His/−Leu/−Trp plates supplemented with 50 mM 3‐AT.

### 2.16 Chromatin Immunoprecipitation (ChIP) Analysis

ChIP was performed using the Magna ChIP HiSens Kit (Sigma‐Aldrich) according to the manufacturer's protocol. Fungal biomass was collected 24 h after chlamydospore induction, cross‐linked with 1% formaldehyde, and quenched with 125 mM glycine. Chromatin was sheared by sonication to 100–1000 bp and immunoprecipitated with anti‐GFP antibody (abcam). Enrichment was quantified by qPCR using the 2^−∆∆Ct^ method (Shin et al. 2024). Primers are listed in Table S2. The detailed protocol is provided in the Supporting Information.

### 2.17 RNA Extraction and Real‐Time Quantitative PCR (RT‐qPCR)

RNA from mycelial samples was extracted by using Trizol Total RNA Isolation Kit (Sangon Biotech). Next, RNA was reverse transcribed to cDNA via HiScript III 1st Strand cDNA Synthesis Kit (Vazyme). RT‐qPCR was performed using ChamQ SYBR qPCR Master Mix (Vazyme). The 2^−∆∆Ct^ method was used to analyse the obtained data. Detailed kit information, cycling parameters, and primer sequences are provided in the Supporting Information and Table S2.

### 2.18 RNA‐Seq Analysis

WT and Δ*WC2* mycelia were harvested at 0, 1 and 3 days after chlamydospore induction on WA plates (supplemented with 2.5 g/L glucose). Library construction and sequencing (Illumina Novaseq X Plus) were performed by Majorbio (Shanghai, China). Reads were filtered by fastp, aligned with HISAT2, and differentially expressed genes were identified using |log_2_(fold change)| ≥ 2 and *p* < 0.05 (Zhang et al. 2023).

### 2.19 DNA Affinity Purification Sequencing (DAP‐Seq) Analysis

The cDNA sequence of the *WC2* gene was inserted into the Halo‐tagged DB3 vector. Subsequently, this plasmid, along with the genomic DNA of *A. flagrans* , was dispatched to Genedenovo Biotechnology Co. Ltd. (Guangzhou, China) for WC2‐halo protein expression and DAP‐seq analysis. Two independent immunoprecipitation (IP) experiments were performed, with the input group (without the addition of WC2‐halo protein) serving as the control. The purified DNA products were then subjected to sequencing on the Illumina platform to analyse and identify the putative DNA binding sites of the target protein across the whole genome (Bai et al. 2023).

### 2.20 Statistical Analyses

All quantitative data were derived from at least three biologically independent replicates and are expressed as mean ± standard deviation (SD). Statistical comparisons between two groups were performed by two‐tailed Student's *t*‐tests in GraphPad Prism (version 10). Asterisks indicate statistical significance: *p < 0.05; **p < 0.01; ***p < 0.001.

## 3 Results

### 3.1 Sequence Analysis and Gene Expression Pattern of Eight GATA‐Type Transcription Factors in *A. flagrans*

**FIGURE 1:**
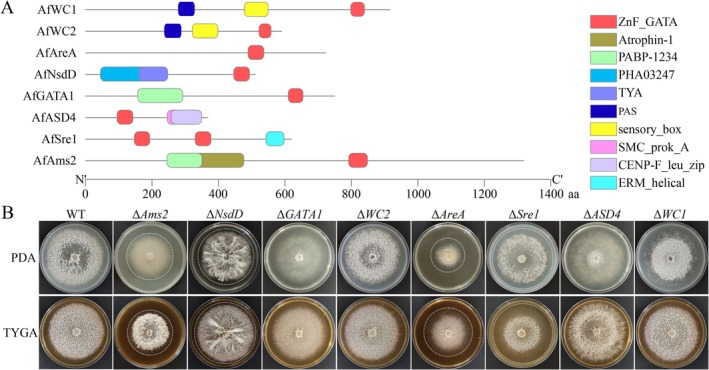
Identification of eight GATA‐type transcription factors in *A. flagrans* and analysis of their effects on hyphal growth. (A) Analysis of conserved domains of the eight GATA‐type transcription factors in *A. flagrans* . (B) Colonies of the WT and eight GATA‐type transcription factor mutants grown on PDA and TYGA plates at 28°C for 5 days. White circles indicate colony margins.

Based on the genomic data of *A. flagrans* (Zhang et al. 2023), we identified eight GATA‐type transcription factors, namely WC2, WC1, Sre1, NsdD, AreA, Ams2, ASD4, and GATA1, all of which contain conserved ZnF_GATA domains and characteristic amino acid residues (Figures 1A and S1, and Table S3). Phylogenetic analysis revealed that seven GATA‐type transcription factors in *A. flagrans* clustered with their known homologues from other filamentous fungi, whereas GATA1 likely represents a novel member that is distinct from all functionally characterized GATA‐type transcription factors (Figure S2). Further analysis classified the DNA‐binding GATA‐type domain sequences of the eight transcription factors into four distinct patterns (Table S3): CX_2_CX_17_CX_2_C (Sre1, AreA, ASD4), CX_2_CX_18_CX_2_C (WC2, WC1, NsdD), CX_2_CX_20_CX_2_C (GATA1), and a notable mutant form CX_2_CX_18_EX_2_S (Ams2). These sequence differences suggest substantial divergence in the downstream genes regulated by these factors, implying corresponding functional diversity. Notably, transcriptomic analysis revealed that all eight GATA‐type transcription factors were upregulated to varying degrees during both trap and chlamydospore formation, a pattern consistently confirmed by RT‐qPCR (Figure S3). These findings suggest that these eight GATA‐type transcription factors are likely involved in important biological functions related to pathogenicity and chlamydospore formation (Figure S3).

### 3.2 Generation of Gene Deletion Mutants and Complementation Strains for All Eight GATA‐Type Transcription Factors

To investigate the biological functions of the eight GATA‐type transcription factors in *A. flagrans* , we performed targeted gene knockout via homologous recombination for each factor. All knockout events were verified using a set of three specific primer pairs (Figures S4–S11). Furthermore, complementation strains were generated by reintroducing the respective native element, including ORF, promoter, and terminator regions, into the corresponding deletion mutants. RT‐qPCR analysis confirmed that the expression level of each GATA‐type transcription factor in its complementation strain was restored to a level comparable to that in the WT strain (Figure S12A).

### 3.3 GATA‐Type Transcription Factors Differentially Regulate Growth and Stress Responses in *A. flagrans*

To assess the impact of the eight GATA‐type transcription factor genes on growth, WT and mutants were inoculated on both PDA and TYGA media and cultured for 5 days. The two media yielded consistent results: deletion of *Sre1*, *AreA*, *Ams2* or *NsdD* reduced radial growth, indicating that these genes positively regulate hyphal extension in *A. flagrans* . In contrast, the deletion of *WC2*, *WC1*, *GATA1* or *ASD4* showed no significant effect on mycelial growth (Figures 1B and S13). Notably, the Δ*NsdD* mutants produced abundant, cotton‐like aerial hyphae (Figure 1B), a phenotype that aligns with observations in Δ*NsdD* or Δ*FlbD* mutants of *A. flagrans* , *A. nidulans* , *A. niger* , and *Fusarium graminearum* (Ojeda‐López et al. 2018; Zhang et al. 2025). This consistency suggests a conserved role for NsdD/FlbD in regulating aerial hyphal morphology. The complementation strains for all eight GATA‐type transcription factors showed no significant difference in mycelial growth compared to the WT strain (Figure S12B).

To investigate the role of the eight GATA‐type transcription factors in stress adaptation, WT and mutants were cultured on PDA medium supplemented with gradient concentrations of sodium chloride (NaCl), sorbitol, sodium dodecyl sulfate (SDS), Congo red (CR), and hydrogen peroxide (H_2_O_2_). The results demonstrated that high concentrations of all five chemical stressors inhibited the growth of both the WT and the eight GATA‐type transcription factor knockout strains. In contrast, lower concentrations of NaCl (0.1 M) and sorbitol (0.25 M), as well as Congo red (25 mM), enhanced the growth of the Δ*AreA* and Δ*GATA1* mutants, respectively (Figures S14–17). In addition, the stress‐response profiles of Δ*WC2*, Δ*WC1*, Δ*NsdD*, and Δ*Ams2* mutants were highly correlated across all five chemical stressors, whereas the responses of Δ*AreA* and Δ*Sre1* mutants showed a strong correlation specifically under SDS and Congo red stress (Figures S14–S17). Δ*WC2*, Δ*WC1*, Δ*NsdD*, Δ*Ams2*, and Δ*ASD4* mutants exhibited reduced sensitivity to 0.02% SDS compared to the WT strain, whereas Δ*AreA*, Δ*Sre1*, and Δ*GATA1* mutants displayed increased sensitivity (Figures S15 and S17A). For Congo red stress, the sensitivities of Δ*WC2*, Δ*WC1*, Δ*AreA*, and Δ*Sre1* mutants to Congo red (25–100 mM) were increased relative to the WT, while no significant change was observed for Δ*NsdD* and Δ*Ams2* mutants (Figures S15 and S17B). When exposed to sorbitol (0.25 M), Δ*WC2* and Δ*WC1* mutants exhibited reduced sensitivity, whereas their sensitivity to NaCl (0.1 M and 0.2 M) remained unchanged (Figure S17C). Similarly, Δ*Sre1* and Δ*GATA1* mutants showed no significant change in sensitivity to either sorbitol (0.25 and 0.5 M) or NaCl (0.1 and 0.2 M) (Figure S17D). In contrast, Δ*NsdD* and Δ*Ams2* mutants displayed reduced sensitivity to both sorbitol and NaCl at these concentrations (Figure S17). Furthermore, all knockout mutants except Δ*ASD4* exhibited increased sensitivity to H_2_O_2_ (Figures S16 and S17E). In summary, the distinct susceptibility profiles indicate that the GATA‐type transcription factors perform overlapping and specialized functions in mediating the fungal response to diverse environmental stresses.

### 3.4 Contribution of GATA‐Type Transcription Factors to Fungal Pathogenicity

**FIGURE 2:**
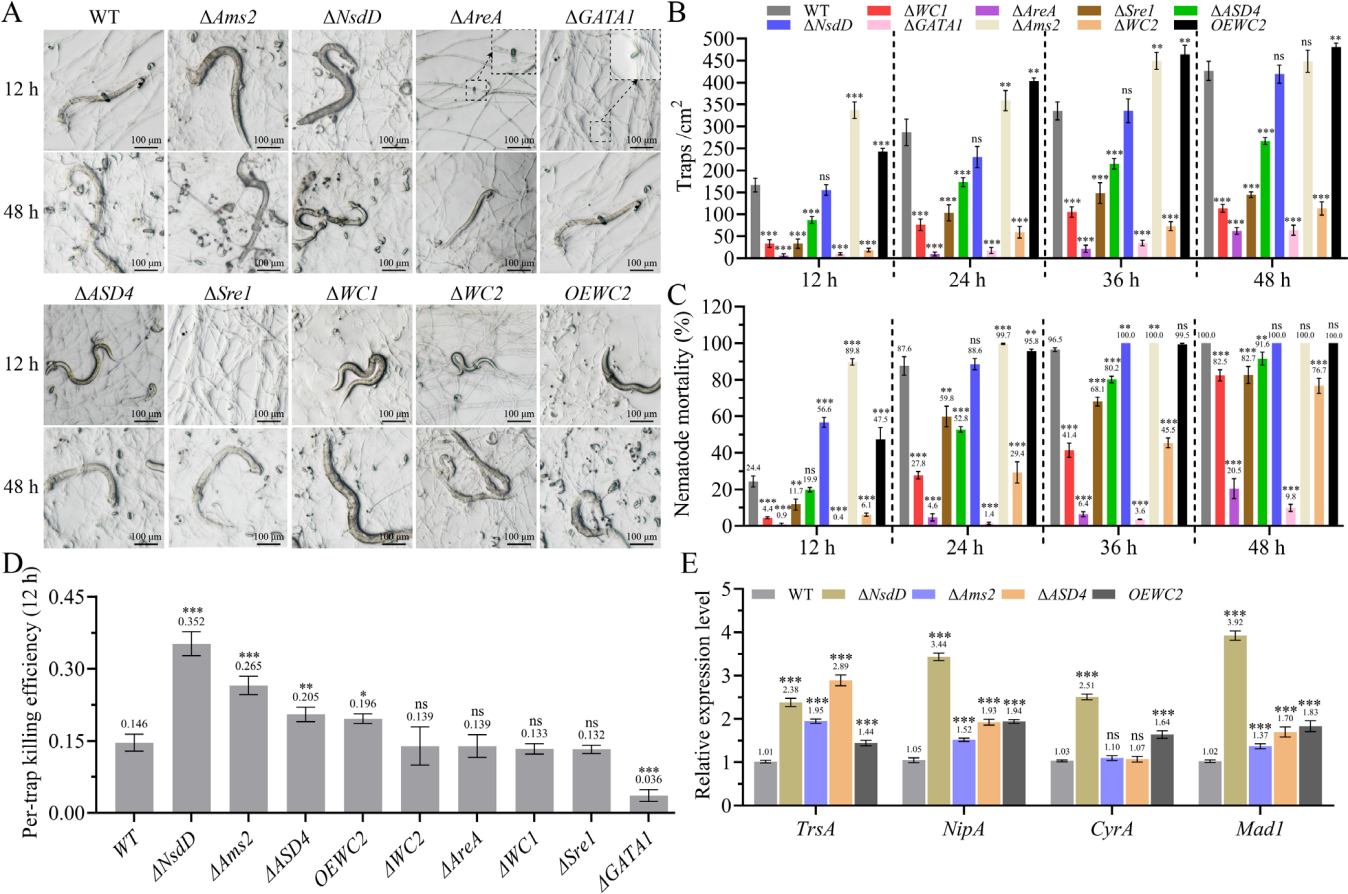
Comparison of trap formation and pathogenicity between WT and eight GATA‐type transcription factor mutants. (A) The representative images of trap and pathogenicity at 12 and 48 h, respectively. (B, C) Quantification of the number of traps (B) and nematode mortality (C) by WT and eight GATA‐type transcription factor mutants at 12, 24, 36 and 48 h. (D) Per‐trap killing efficiency at 12 h post‐nematode addition. Data are presented as mean ± SD from three independent replicates. Higher values indicate higher per‐trap killing efficiency. (E) RT‐qPCR analysis of the expression levels of four virulence factors in the Δ*NsdD*, Δ*Ams2*, Δ*ASD4* and OE*WC2* strains. Student's *t*‐test; *p < 0.05, **p < 0.01, ***p < 0.001.

Predatory activity against nematodes represents a key ecological function of *A. flagrans* (Zhang et al. 2023). In the WT strain, approximately 150 traps/cm^2^ were produced within 12 h after adding *C. elegans* , with a nematode mortality rate of about 24.4% (Figure 2A–C). Trap numbers increased over time, and nematode mortality reached 87.6%, 96.5%, and 100% at 24, 36, and 48 h, respectively (Figure 2B). However, Δ*WC1*, Δ*WC2*, Δ*AreA*, Δ*Sre1*, Δ*GATA1* and Δ*ASD4* mutants produced fewer traps and exhibited lower nematode mortality (4.4%, 6.1%, 0.4%, 11.7%, 0.9% and 19.9%, respectively) than the WT strain at 12 h (Figure 2A–C). At 48 h, the nematode mortality in these mutants maintained the same trend observed at 12 h. WC1, WC2, AreA, Sre1, GATA1 and ASD4 are positive regulators of trap formation and pathogenicity (Figure 2). Among them, Δ*AreA* and Δ*GATA1* mutants only formed immature, club‐like protrusions rather than fully developed ring‐shaped traps at 12 h. Mature ring traps were not observed in these mutants until 48 h (Figure 2A). Δ*WC2*, Δ*AreA* and Δ*GATA1* mutants showed the strongest impairment in pathogenicity. Meanwhile, the overexpression strain *OEWC2* showed increased trap formation and enhanced pathogenicity compared with the WT strain (Figure 2). Furthermore, complementation assays confirmed the full restoration of WT levels in both trap formation and nematode mortality for the eight GATA‐type transcription factor mutants (Figure S18). Notably, at 12 h, the Δ*Ams2* mutants produced significantly more traps (~330/cm^2^) (Figure 2B) and achieved a higher nematode mortality rate (~89.8%) than the WT strain (Figure 2C). The nematode mortality of the Δ*Ams2* mutants further increased to 99.7% at 24 h. Ams2 functions as a negative regulator of trap‐mediated pathogenicity. Interestingly, from 12 to 48 h, the Δ*NsdD* mutants produced a comparable number of traps to the WT strain, yet their nematode mortality was significantly higher, reaching 56.6%, 88.6%, and 100% at 12, 24, and 36 h, respectively (Figure 2C).

The discrepancy between trap formation and nematode mortality led us to speculate that some GATA‐type transcription factors may modulate pathogenicity through mechanisms other than simply increasing trap number. To test this hypothesis, we analysed the per‐trap killing efficiency for each strain. At 12 h, the per‐trap killing efficiencies of the Δ*NsdD*, Δ*Ams2*, Δ*ASD4*, and OE*WC2* strains were 2.41‐, 1.82‐, 1.40‐, and 1.34‐fold higher than that of the WT strain, respectively, indicating a significant improvement in the intrinsic killing capacity of individual traps produced by these strains (Figure 2D, Table S4). At 24 h, as WT mortality approached saturation (~88%), the differences in per‐trap killing efficiency between most mutants and the WT became less pronounced. In contrast, the Δ*GATA1* mutants showed a drastically reduced per‐trap killing efficiency, which was only 25% and 23% of the WT level at 12 and 24 h, respectively, consistent with its severe defects in trap morphogenesis and pathogenicity (Table S4). Notably, the observed discrepancy between trap formation and nematode mortality in certain mutants suggests that these transcription factors may regulate pathogenicity by modulating virulence factors rather than simply affecting trap number.

During the nematode predation process of NT fungi, virulence factors (NipA, TrsA, CyrA), extracellular proteases, and the adhesive protein Mad1 on the trap surface play crucial roles (Zou et al. 2010; Soares et al. 2013; Li et al. 2016; Emser et al. 2024, 2025). Given that the Δ*NsdD*, Δ*Ams2*, and Δ*ASD4* mutants exhibited significantly enhanced per‐trap killing efficiency, we examined the expression levels of these virulence factors and the adhesin in these mutants. In all three mutants, the transcript levels of *NipA*, *TrsA*, and *Mad1* were markedly upregulated, whereas *CyrA* was upregulated only in the Δ*NsdD* mutants and remained unchanged in the Δ*Ams2* and Δ*ASD4* mutants (Figure 2E). Consistently, overexpression of *WC2* led to significantly increased expression of *NipA*, *TrsA*, *CyrA*, and *Mad1* compared to the WT strain (Figure 2E). Furthermore, deletion of any GATA‐type transcription factor, with the exception of Ams2, reduced protease activity, while overexpression of WC2 enhanced it (Figure S19). This suggests that Ams2, NsdD, and ASD4 may negatively modulate certain virulence factors and adhesins required for efficient nematode killing, whereas WC2 acts as a positive regulator of these virulence‐associated genes (Figure 2).

In summary, our results define Ams2 as a negative regulator and WC1, WC2, AreA, Sre1, GATA1, and ASD4 as positive regulators of trap formation in *A. flagrans* , whereas NsdD, Ams2, and ASD4 act as potential suppressors of virulence effectors during nematode predation. These functionally validated regulators represent a suite of actionable targets for genetically improving biocontrol efficacy.

### 3.5 Loss of GATA‐Type Transcription Factors Results in Defective Chlamydospore Formation

**FIGURE 3:**
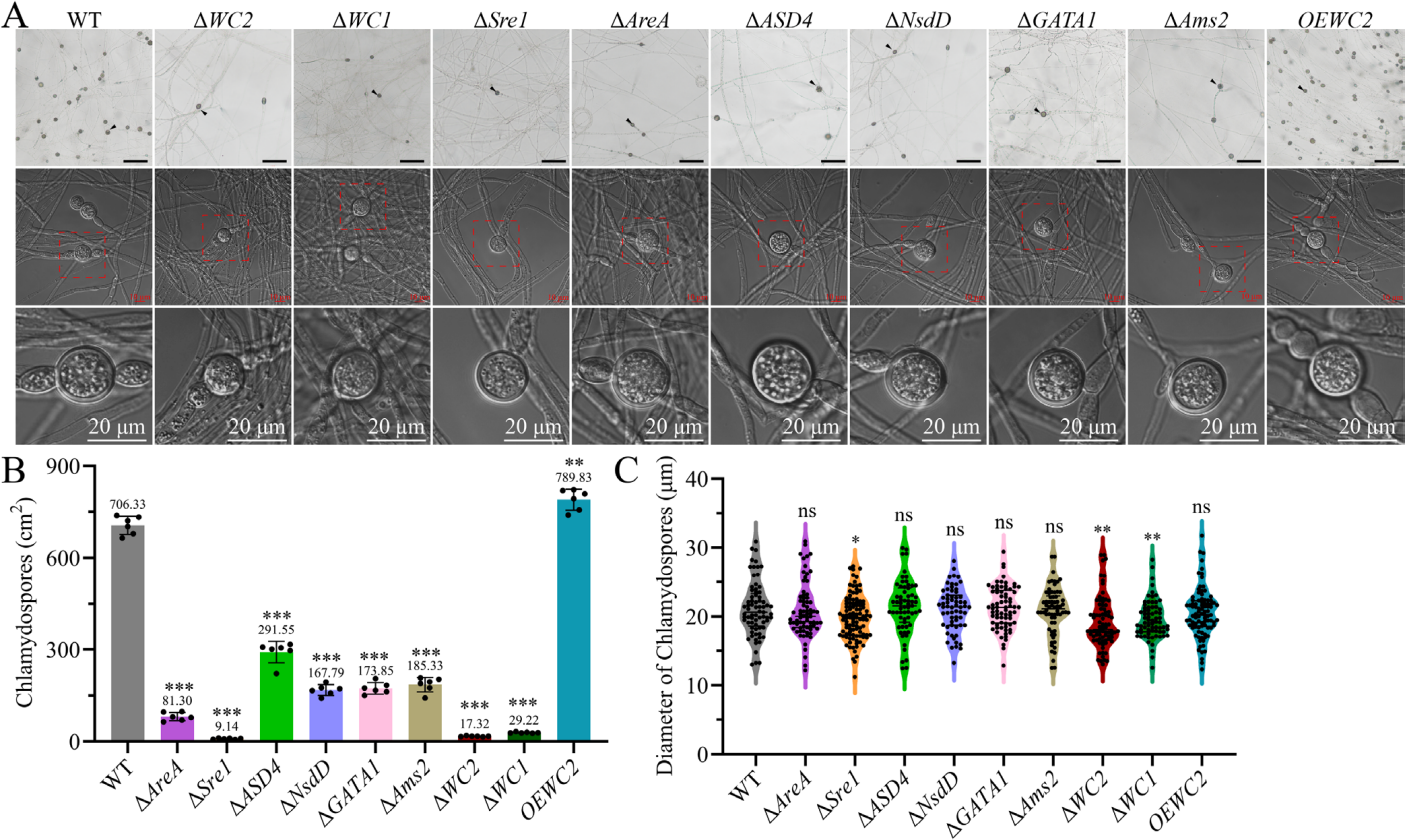
Functional analysis of eight GATA‐type transcription factors in chlamydospore formation. (A) Morphology of chlamydospores produced by the WT and eight GATA‐type transcription factor mutants after 14 days of culturing on WA medium. Scale bar, 100 μm. (B) The number of chlamydospores (Student's *t*‐test; *p < 0.05, **p < 0.01, ***p < 0.001). (C) Statistical analysis of chlamydospore diameter. The diameter of 30 chlamydospores per strain was randomly measured using Adobe Photoshop software (Student's *t*‐test; *p < 0.05, **p < 0.01, ***p < 0.001).

To investigate the regulatory roles of GATA‐type transcription factors in chlamydospore formation in *A. flagrans* , we compared both the number and morphology of chlamydospores produced by the mutants (Figure 3). The results showed that the Δ*WC2*, Δ*WC1*, Δ*Sre1*, Δ*GATA1*, Δ*AreA*, Δ*Ams2*, Δ*ASD4*, and Δ*NsdD* mutants produced significantly fewer chlamydospores than the WT strain, with yields corresponding to only 2.45%, 4.14%, 1.29%, 24.61%, 11.51%, 26.24%, 41.27%, and 23.75% of the WT level, respectively (Figure 3A,B). In addition, the diameter of chlamydospores was significantly smaller in the Δ*WC1*, Δ*WC2* and Δ*Sre1* mutants, whereas deletion of the other genes had no significant effect on chlamydospore size (Figure 3C). Overexpression of *WC2* increased chlamydospore production by approximately 11.82% relative to the WT, but did not significantly alter chlamydospore diameter (Figure 3B). Furthermore, complementation assays revealed that both the morphology and number of chlamydospores in the GATA‐type transcription factor complementation strains were comparable to those in the WT strain (Figure S20). Together, these findings demonstrate that all eight GATA‐type transcription factors play important roles in chlamydospore development. Notably, WC1, WC2, and Sre1 regulate not only the number but also the diameter of chlamydospores (Figure 3). WC1 and WC2 are light‐responsive transcription factors, yet whether their regulation of chlamydospore formation depends on light conditions remains unclear. Therefore, we examined the effect of light on chlamydospore formation. The results showed that chlamydospore formation was not significantly affected under continuous darkness, continuous light, or a 12‐h light/12‐h dark cycle. These results indicate that light does not influence chlamydospore formation and that the regulatory roles of WC1 and WC2 in this process are light‐independent (Figure S21).

### 3.6 Identification of the Protein Interaction Network of GATA‐Type Transcription Factors

**FIGURE 4:**
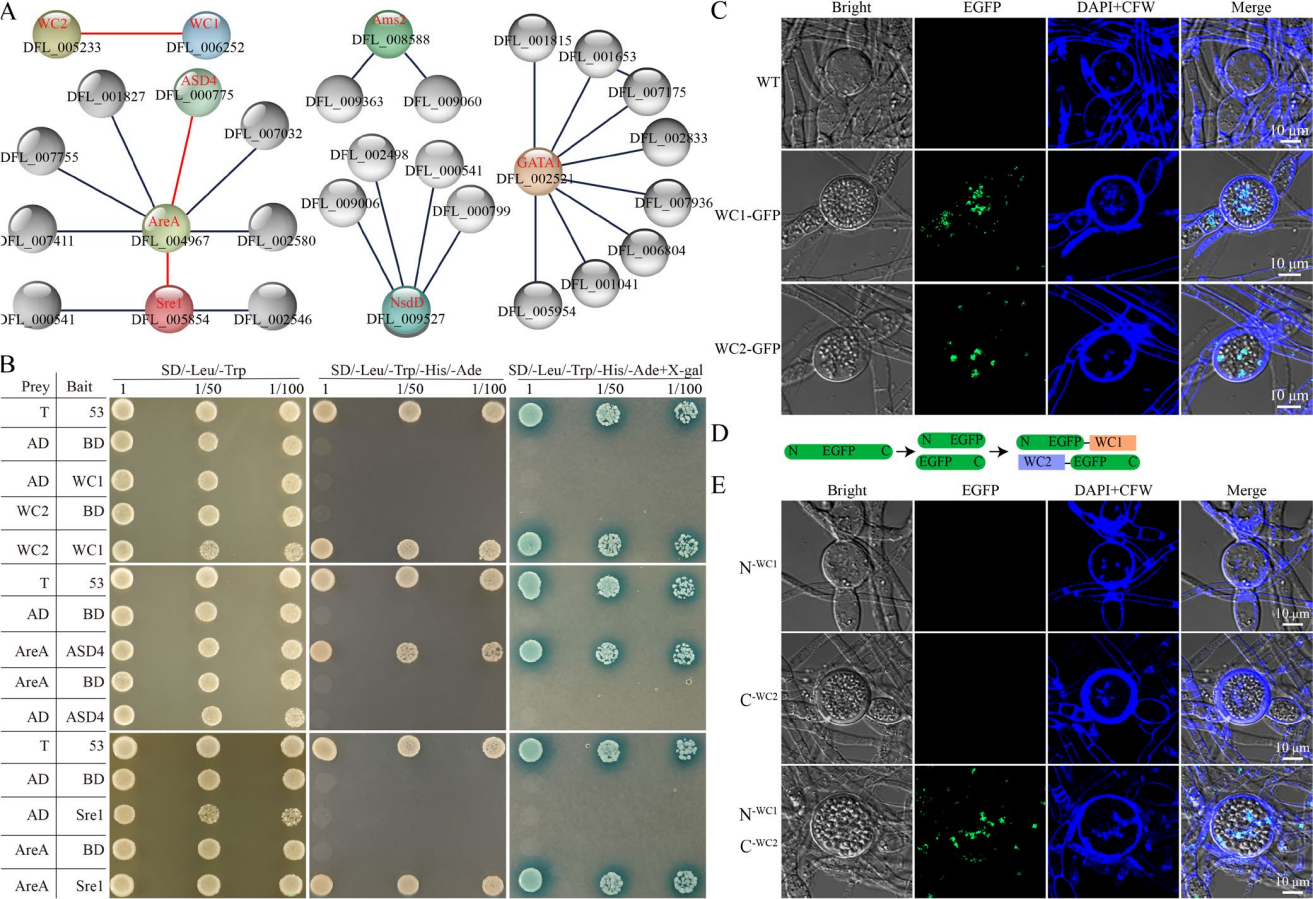
Interaction network construction of the eight GATA‐type transcription factors. (A) Prediction of interacting proteins for the eight GATA‐type transcription factors in *A. flagrans* using STRING software (http://string‐db.org/). (B) Y2H analysis of the interactions between WC1 and WC2, and between AreA and Sre1/ASD4. (C) Confocal microscopy analysis of the subcellular localization of WC1 and WC2. (D) Verification of the interactions between WC1 and WC2 during chlamydospore formation by BiFC assay. (E) Confocal microscopy analysis of WC1‐WC2 interaction localized to the nucleus of chlamydospores.

To elucidate the molecular mechanisms underlying the biological functions of GATA‐type transcription factors, we predicted their potential protein–protein interactions using the STRING tool (Figure 4A, Table S5). These predictions identified specific putative interactions: WC1‐WC2 and AreA‐ASD4‐Sre1. Conversely, Ams2, NsdD, and GATA1 did not show strong predicted connections within this set, implying they may function with other partners (Figure 4A). This predicted interaction network was further validated experimentally. Y2H assays confirmed the specific interaction between WC1 and WC2, as well as the interactions of AreA with ASD4 and Sre1 (Figure 4B). Phenotypic analysis revealed that WC1 and WC2 share consistent biological roles in both trap formation and chlamydospore development in *A. flagrans* (Figures 2 and 3), suggesting that their physical interaction may mediate the coordinated regulation of these processes. To investigate their subcellular localization, we generated GFP‐tagged versions of WC1 and WC2. Fluorescence microscopy showed that both proteins are localized to the nucleus in chlamydospores (Figure 4C). Importantly, BiFC assays demonstrated that WC1 and WC2 interact directly and co‐localize in the nuclei of chlamydospores (Figure 4D,E). These results demonstrate that the light‐responsive complex WC1/WC2 plays a crucial role in chlamydospore formation.

### 3.7 Transcriptomic Insight Into the Regulatory Role of WC2 in *A. flagrans*

To investigate the regulatory function of GATA‐type transcription factors, we selected WC2, which exhibits the most significant impact on both chlamydospore and trap formation, as the target for RNA‐seq analysis. Mycelial samples of the WT strain and the Δ*WC2* mutants were collected at 0, 1, and 3 days after chlamydospore induction for RNA‐seq analysis. Principal component analysis revealed substantial differences between the WT and Δ*WC2* mutants across the time points, while the three biological replicates of both the WT and Δ*WC2* mutants showed high similarity (Figure S22A). Differential expression analysis identified 2397, 1322, and 1697 DEGs in the Δ*WC2* mutants at day 0, day 1, and day 3, respectively (Figure S22B). Venn analysis indicated that 301 DEGs in the Δ*WC2* mutants were common to both the mycelial growth stage (day 0) and the chlamydospore formation stages (day 1 and day 3), whereas 1583 DEGs were specifically expressed during the mycelial growth stage (Figure S22C).

Further KEGG annotation analysis of the DEGs in the Δ*WC2* mutants during the mycelial growth stage (day 0) revealed that the top 20 enriched pathways primarily included: Biosynthesis of other secondary metabolites, Metabolism of terpenoids and polyketides, Nucleotide metabolism, Glycan biosynthesis and metabolism, Metabolism of other amino acids, Energy metabolism, Metabolism of cofactors and vitamins, Lipid metabolism, Amino acid metabolism, Carbohydrate metabolism, Replication and repair, Transcription, Folding, sorting and degradation, Translation, Membrane transport, Signal transduction, Cell growth and death, Transport and catabolism, and Aging (Figure S22D). The KEGG terms enriched for differentially expressed genes at the chlamydospore formation stage (day 1) largely overlapped with those at the mycelial growth stage (day 0). Among these, Carbohydrate metabolism, Amino acid metabolism, Translation, and Transport and catabolism contained the highest numbers of annotated genes (Figure S22E). We propose that WC2 may influence chlamydospore formation by regulating the above biological processes.

### 3.8 Integration of Transcriptome and DAP‐Seq Data Identifies the Core Regulatory Network Directly Controlled by WC2

**FIGURE 5:**
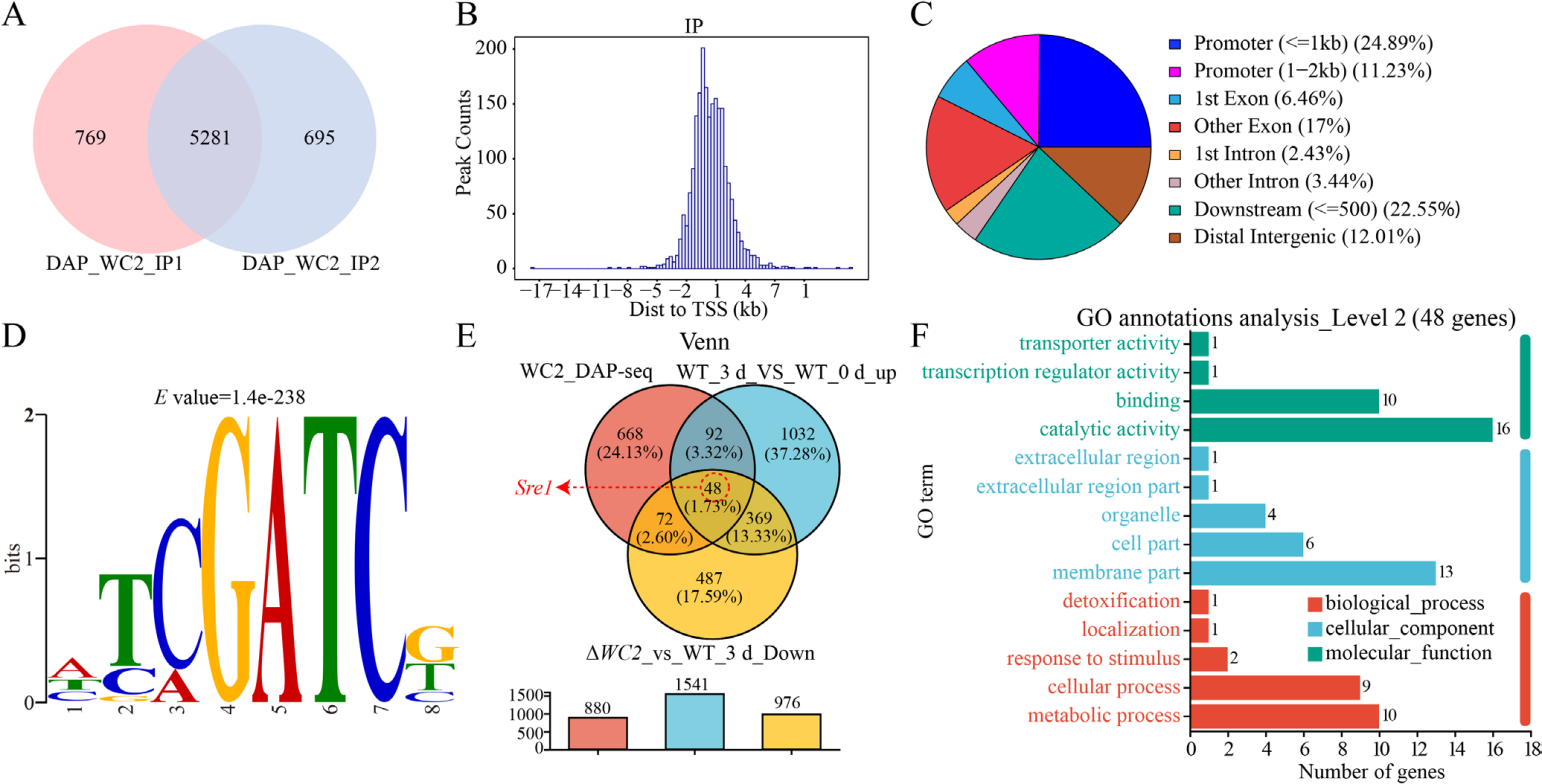
DAP‐seq analysis of WC2 in *A. flagrans* . (A) Western blot analysis of WC2 protein expressed and purified using a plant expression system. (B) Distribution of distances between obtained peaks and transcription start sites (TSS). (C) Correspondence of obtained peaks to various functional regions of genes. (D) Positional distribution of motifs enriched significantly in the WC2 target peaks. (E) Integrated DAP‐seq and RNA‐seq analysis of downstream target genes of WC2 during chlamydospore formation. (F) GO enrichment analysis of WC2 downstream target genes during chlamydospore formation.

To further investigate the regulatory mechanism of WC2 in *A. flagrans* , we performed DAP‐seq analysis to identify the downstream target genes of WC2. The results indicated that the peaks from the two IP experiments exhibited a high degree of reproducibility (Figure 5A). Annotation of peaks from two independent IP experiments revealed that most WC2 binding sites were located within 2 kb of transcription start sites (TSS) (Figure 5B), with 36.12% of these sites located in promoter regions (−2 kb) (Figure 5C). Using MEME‐ChIP to detect significantly enriched motifs, we identified the conserved DNA‐binding motif of WC2 as 5′‐(T/G/C)(C/A)GATC(A/G/C)‐3′ (*E*‐value = 1.4e−238). KEGG functional annotation of potential downstream genes regulated by WC2 showed that the top 20 enriched terms were consistent with the RNA‐seq results, mainly involving carbohydrate metabolism, amino acid metabolism, translation, and transport and catabolism (Figure S23A). GO enrichment analysis revealed that the top 20 terms were primarily associated with catalytic activity, binding, cell part, membrane part, metabolic process, and cellular process (Figure S23B).

Furthermore, to identify downstream target genes directly regulated by WC2 during chlamydospore formation, we performed a Venn analysis integrating differentially expressed genes from the transcriptome and peaks from the DAP‐seq data. The results indicated that 48 genes containing WC2 binding sites were significantly upregulated during chlamydospore formation and downregulated in the Δ*WC2* mutants (Figure 5D, Table S6). GO annotation of these genes showed enrichment in binding, catalytic activity, cell part, membrane part, metabolic process, and cellular process (Figure 5F). Notably, the GATA‐type transcription factor Sre1 was identified as a downstream target of WC2 (Figure 5E), suggesting cross‐regulation among GATA‐type transcription factors.

To investigate the regulatory interactions among GATA‐type transcription factors, we examined the expression levels of the other seven GATA‐type transcription factors in the Δ*WC2* mutants (Figure S24). The results showed that, compared with the WT strain, the expression levels of *Ams2*, *NsdD*, *ASD4*, and *Sre1* were downregulated in the Δ*WC2* mutants during the hyphal stage, whereas the expression levels of *GATA1*, *WC1*, and *AreA* were significantly upregulated (Figure S24). During chlamydospore formation, the expression levels of *AreA*, *ASD4*, *NsdD*, and *WC1* were markedly upregulated in the Δ*WC2* mutants, while those of *Ams2*, *GATA1*, and *Sre1* were significantly downregulated. These findings indicate that GATA‐type transcription factors mutually influence each other across different developmental stages in *A. flagrans* (Figure S24).

### 3.9 WC1 and WC2 Regulate Chlamydospore and Trap Formation by Activating *Sre1*

**FIGURE 6:**
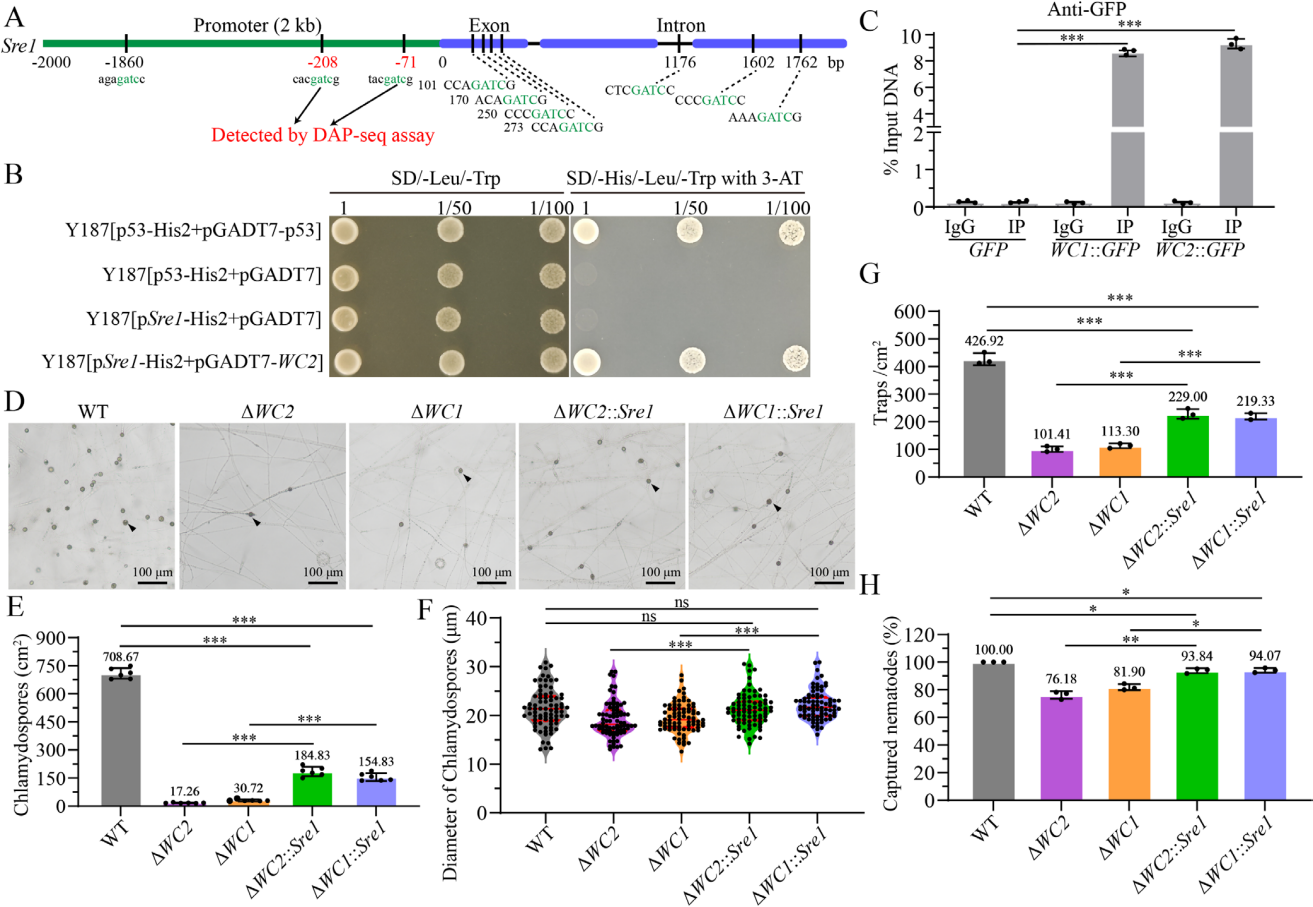
Analysis of the regulatory roles of WC2 and WC1 on Sre1. (A) DAP‐seq analysis of the DNA‐binding motifs of WC2 within the promoter and ORF regions of the *Sre1* gene. (B) Validation of the regulatory role of WC2 on *Sre1* by Y1H assay. Y187[p53‐His2 + pGADT7‐p53] was used as the positive control, while Y187[p53‐His2 + pGADT7] served as the negative control. (C) ChIP‐qPCR analysis of binding of WC1 and WC2 to the promoters of *Sre1* gene at day 1 of chlamydospore formation. A transformant carrying a GFP plasmid was used as the control strain. Enrichment using Anti‐GFP antibody (Student's *t*‐test; ***p < 0.001). (D) Comparative analysis of chlamydospore formation after expression of the *Sre1* gene in Δ*WC2* and Δ*WC1* mutants. (E, F) Comparative analysis of the number (E) and diameter (F) of chlamydospores. (G, H) Comparative analysis of the number of traps (G) and pathogenicity (H) (Student's *t*‐test; *p < 0.05, **p < 0.01, ***p < 0.001).

The integrated analysis of RNA‐seq and DAP‐seq data suggested that Sre1 may function as a downstream transcription factor of WC2 involved in chlamydospore formation. We analysed the GATA motifs located in the promoter, exons, and introns of the *Sre1* gene. The results revealed three GATA motifs in the promoter region of *Sre1*, two of which were detected in the DAP‐seq assay. In addition, six GATA motifs were identified in the exons and one in the intron (Figure 6A). Y1H assays demonstrated that WC2 can directly bind to the *Sre1* promoter (Figure 6B). Furthermore, ChIP‐qPCR assays showed significant enrichment of the *Sre1* promoter fragment in the *WC1*::*GFP* and *WC2*::*GFP* strains, confirming the binding ability of WC1 and WC2 to the promoter region of the *Sre1* gene (Figure 6C).

To verify the regulatory role of WC1 and WC2 on Sre1 during chlamydospore and trap formation, we examined whether the expression of *Sre1* in the Δ*WC1* and Δ*WC2* backgrounds could rescue the phenotypic defects of Δ*WC1* and Δ*WC2* mutants. The results showed that *Sre1* expression partially restored the impaired production of chlamydospores and traps in both mutants (Figure 6D). Specifically, *Sre1* expression increased chlamydospore production by approximately 4.04‐fold in the Δ*WC1* mutants and 9.7‐fold in the Δ*WC2* mutants and restored chlamydospore diameter to the WT level (Figure 6E,F). Furthermore, the formation of traps was found to be enhanced by approximately 0.94‐fold in Δ*WC1* mutants and 1.26‐fold in Δ*WC2* mutants upon *Sre1* expression, accompanied by a significant improvement in nematocidal activity (Figure 6G,H). These findings indicate that WC2 and WC1 activate *Sre1* expression to regulate chlamydospore and trap formation.

**FIGURE 7:**
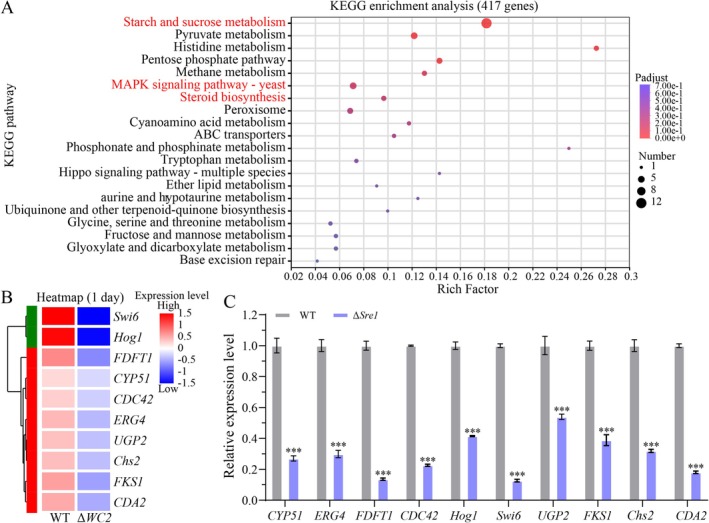
Identification and expression analysis of potential downstream genes of Sre1. (A) KEGG enrichment analysis of the 417 genes that were significantly upregulated during chlamydospore formation in the WT strain and downregulated in the Δ*WC2* mutants. (B) Expression profiles of ten selected candidate genes in the Δ*WC2* mutants based on RNA‐seq data. (C) RT‐qPCR validation of the ten candidate genes in the WT and Δ*Sre1* strains. Data are presented as mean ± SD from three independent biological replicates. Asterisks indicate significant differences compared to the WT strain (Student's *t*‐test; ***p < 0.001).

To further identify the downstream target genes of Sre1, we first performed KEGG enrichment analysis on the 417 genes that were significantly upregulated during chlamydospore formation in the WT strain and downregulated in the Δ*WC2* mutants. These genes were significantly enriched in pathways including starch and sucrose metabolism, MAPK signalling pathway, steroid biosynthesis, pyruvate metabolism, histidine metabolism, pentose phosphate pathway, and methane metabolism (Figure 7A). Based on these enrichments and functional annotations, we selected ten candidate genes for RT‐qPCR validation in the WT and Δ*Sre1* strains (Figure 7B, Table S7). These genes represent three functional categories critical for fungal development and pathogenicity. The first category comprises genes involved in ergosterol biosynthesis: FDFT1 (squalene synthase), ERG4 (delta(24(24(1)))‐sterol reductase), and CYP51 (cytochrome P450), which are essential for membrane sterol production and integrity (Yuan et al. 2025). Ergosterol is the major sterol in fungal membranes and is essential for various cellular processes such as cell growth, signalling, and stress responses (Yuan et al. 2025). In *Cryptococcus neoformans* and *B. cinerea* , Sre1 has been demonstrated to regulate ergosterol biosynthesis, thereby participating in conidiation. Overexpression of ergosterol biosynthesis‐related genes (*ERG2*, *ERG11*, *ERG25* and *ERG26*) can partially rescue the conidiation defects of sre1 knockout strains in *C. neoformans* (Matha et al. 2024). The second category includes components of the MAPK signalling pathway: CDC42 (a serine/threonine protein kinase), Hog1 (mitogen‐activated protein kinase), and Swi6 (a transcription factor), which play central roles in stress responses, cell wall integrity, and developmental signalling. The third category encompasses genes related to cell wall biogenesis: UGP2 (UTP‐glucose‐1‐phosphate uridylyltransferase) involved in UDP‐glucose synthesis, FKS1 (1,3‐beta‐glucan synthase) for β‐glucan synthesis, Chs2 (chitin synthase 2) for chitin synthesis, and CDA2 (chitin deacetylase) for chitosan biosynthesis. RT‐qPCR analysis confirmed that the expression levels of all ten genes were significantly downregulated in the Δ*Sre1* mutants compared to the WT strain, showing high consistency with the RNA‐seq data (Figure 7C). These results demonstrate that Sre1 positively regulates a diverse set of downstream genes involved in membrane homeostasis, stress signalling and cell wall remodelling, suggesting its pleiotropic role in chlamydospore and trap formation.

## 4 Discussion

**FIGURE 8:**
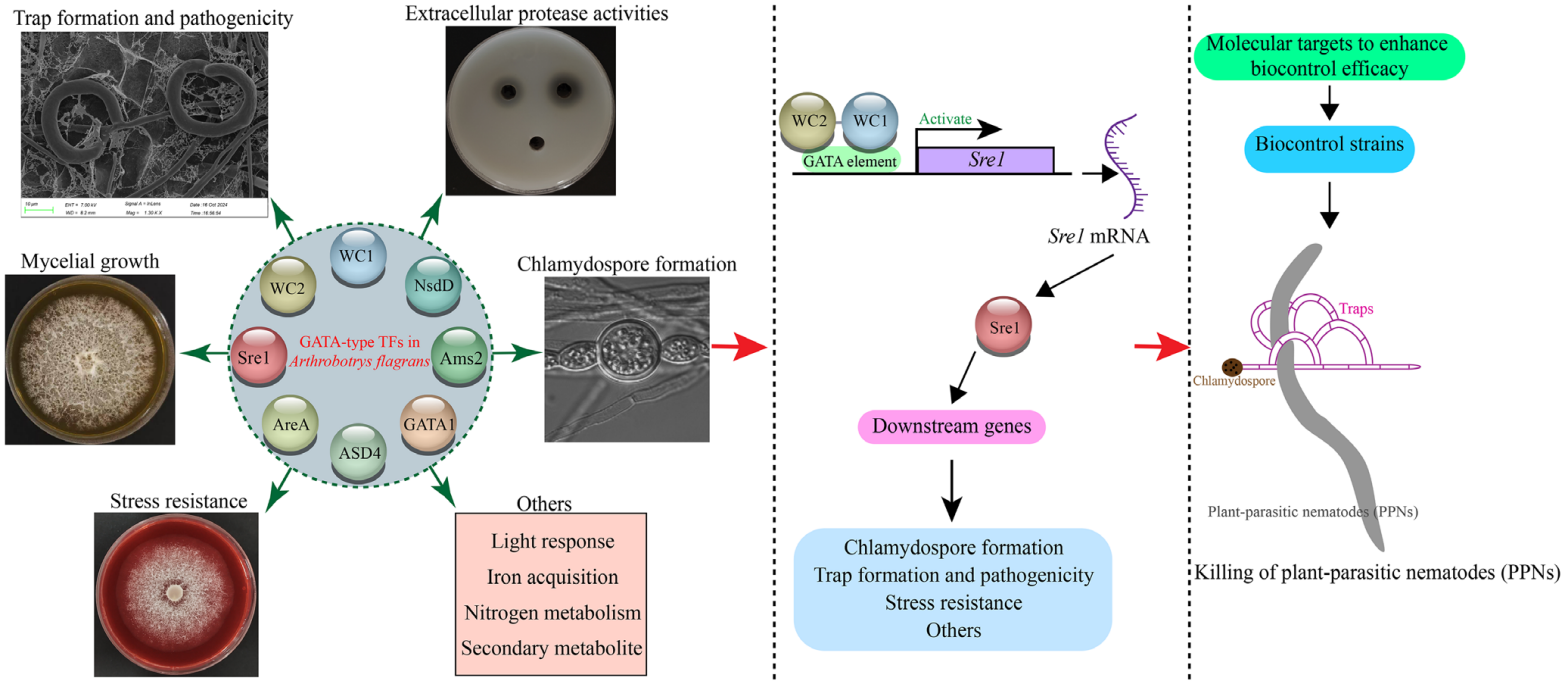
Schematic illustration of the regulation of GATA‐type transcription factors in *A. flagrans* . The eight GATA‐type transcription factors perform diverse functions in *A. flagrans* . The light‐responsive transcription factors WC1 and WC2 interact and act on the GATA elements in the promoter of the GATA‐type transcription factor Sre1, thereby activating *Sre1* gene expression and participating in the formation of chlamydospores and traps. This molecular regulatory network provides promising targets for engineering enhanced biocontrol strains and establishes a theoretical framework for the biological control of plant‐parasitic nematodes (PPNs).

As a key biocontrol agent of parasitic nematodes, *A. flagrans* relies on the trap and chlamydospore formation as central processes for executing its ecological functions and completing its life cycle (Youssar et al. 2019; Zhang et al. 2023). In this study, we systematically characterized eight GATA‐type transcription factors in *A. flagrans* and revealed their coordinated regulation of growth, stress adaptation, pathogenicity, and chlamydospore formation. Importantly, we uncovered a light‐independent WC1/WC2–Sre1 cascade that governs both trap and chlamydospore development (Figure 8).

### 4.1 Functional Divergence of GATA‐Type Transcription Factors Implies a Complex Regulatory Mechanism in *A. flagrans*

In this study, our systematic functional analysis revealed a clear division of labor among the eight GATA‐type transcription factors. WC1, WC2, GATA1 and ASD4 are dispensable for vegetative growth (Figure 1B) but play key roles in pathogenicity (Figure 2) and chlamydospore formation (Figure 3). In contrast, AreA, NsdD, Ams2 and Sre1 are essential for hyphal growth and critically regulate both pathogenicity and chlamydospore formation (Figures 2 and 3). AreA is a central regulator of nitrogen metabolism in fungi, affecting normal hyphal growth in numerous species (Luo et al. 2023; Baldin et al. 2024; Wang et al. 2024). In *A. flagrans* , the growth defect of the Δ*AreA* mutant was partially restored by low concentrations of NaCl or sorbitol, linking its function to osmotic homeostasis (Figure S17). Furthermore, consistent with its role in *A. flagrans* , Ams2 in *Sclerotinia sclerotiorum* positively regulates hyphal growth (Liu et al. 2018). Notably, the involvement of GATA‐type transcription factors in both developmental and metabolic processes parallels the dual role of Velvet proteins in NT fungi, where AfLaeA was shown to regulate trap formation, protease activity, and secondary metabolism (Zhang et al. 2023; Calvo et al. 2024). Such phenotypic differentiation indicates that, despite belonging to the same factor family, these regulators have evolved specialized tasks that together form a multilayered network integrating environmental cues.

### 4.2 GATA‐Type Transcription Factors Constitute a Regulatory Network Orchestrating Pathogenicity

Trap formation is a hallmark event in the lifestyle transition of NT fungi and an indispensable step for nematode predation (Li et al. 2016; Kriegler et al. 2025). In this study, we identified WC1, WC2, AreA, Sre1, GATA1 and ASD4 as positive regulators of trap formation and nematode killing, whereas Ams2 and NsdD act as negative regulators. Among the positive regulators, WC2 plays a key role. In *Botrytis cinerea*, the WC2 homologue BcWCL2 is required for infection cushion development and virulence (Ren et al. 2024), and we found that key trap formation genes were downregulated in the Δ*WC2* mutants (Figure S25). Furthermore, the Δ*AreA* and Δ*GATA1* mutants were only able to form immature knob‐like structures rather than mature ring traps even at 12 h, indicating that these factors are essential for trap morphogenesis (Figure 2). While AreA is best known for nitrogen catabolite repression (Luo et al. 2023), its severe trap defect suggests a direct integration into the predatory developmental program.

Strikingly, the Δ*Ams2* mutants produced excessive traps and exhibited sharply enhanced killing. This phenotype is reminiscent of the transcription factor FlbD, whose deletion leads to spontaneous trap formation and enhanced pathogenicity in *A. flagrans* (Zhang et al. 2025). In *S. sclerotiorum*, Ams2 controls histone gene expression and is required for appressorium (infection cushions) development and virulence (Liu et al. 2018), suggesting its function as a developmental regulator is broadly conserved. More importantly, the Δ*NsdD* mutants formed a normal number of traps but killed nematodes more efficiently, and the Δ*Ams2*, Δ*NsdD* and Δ*ASD4* strains all showed elevated per‐trap killing coupled with upregulation of virulence factors (NipA, TrsA, Mad1) and adhesin Mad1. NipA, TrsA and CyrA are known virulence factors in *A. flagrans* and Mad1 is an adhesin critical for trap‐nematode interaction (Zou et al. 2010; Soares et al. 2013; Li et al. 2016; Emser et al. 2024, 2025). This regulatory uncoupling of trap morphogenesis from functional execution provides a new perspective on the multi‐step predatory process.

### 4.3 GATA‐Type Transcription Factors Are Key Nodes in Chlamydospore Formation and Include a Light‐Independent WC1/WC2 Module

In filamentous fungi, light serves as an important environmental signal that influences various physiological processes, including metabolism, stress response, and development (Yu and Fischer 2019). In *A. flagrans* , all eight GATA‐type transcription factors are required for normal chlamydospore production, and WC1, WC2, and Sre1 additionally control chlamydospore diameter. Although WC1 and WC2 are established light‐responsive factors that mediate blue‐light perception and circadian rhythms in *Neurospora crassa* and other fungi (Froehlich et al. 2002; Ohm et al. 2013), we found that chlamydospore formation proceeds equally well under constant darkness, constant light, or 12 h light/dark cycles, indicating that the WC1/WC2–Sre1 cascade operates independently of light. This suggests that a conserved light‐responsive module has been recruited to regulate chlamydospore development. This is consistent with the light‐independent heterodimerization of BcWCL1 and BcWCL2 via their PAS domains in *B. cinerea* (Ren et al. 2024). In *Schizophyllum commune*, the WC complex also regulates mushroom development through downstream transcription factors independent of its light‐sensing role (Ohm et al. 2013; Vonk et al. 2024). Because *Sre1* expression partially rescues the trap defects of both Δ*WC1* and Δ*WC2* mutants, it is plausible that trap formation is also light‐independent, although this awaits direct testing. In the soil environment, where light is scarce, uncoupling a photoreceptor module from light dependence likely represents an evolutionary adaptation that ensures robust trap and chlamydospore development in darkness. Nutrient stress or developmental cues, rather than light, may therefore trigger WC1/WC2 complex formation and Sre1 activation.

Chlamydospore formation in filamentous fungi is governed by multiple regulators. In *C. albicans* , the MAPK, TOR and Ras‐cAMP‐PKA pathways, along with transcription factors such as Nrg1 and Efg1, regulate chlamydospore development (Hernández‐Cervantes et al. 2020). In *A. flagrans* , the global regulator LaeA, the STRIPAK component SipC, and transcription factors Swi6 and FlbD have been implicated in chlamydospore formation (Wernet et al. 2022; Yu et al. 2022; Zhang et al. 2023, 2025; Linghu et al. 2024; Yang et al. 2025). In this study, we found that all eight GATA‐type transcription factors were downregulated in the Δ*LaeA* mutants, suggesting that LaeA acts upstream of this GATA network to globally coordinate chlamydospore development (Figure S26). Furthermore, the expression levels of positive regulators associated with chlamydospore formation were significantly reduced in the Δ*WC2* mutants, whereas the expression of the negative regulators GCN4 and Ndt80 did not follow this trend (Figure S27). These findings demonstrate that chlamydospore formation results from the interplay of multiple genes, and that GATA‐type transcription factors serve as key regulatory nodes within this developmental network.

### 4.4 The WC1/WC2–Sre1 Cascade Functions as a Novel Module Regulating Both Trap and Chlamydospore Formation

In this study, integrated RNA‐seq, DAP‐seq, Y1H and ChIP‐qPCR analyses demonstrated that the WC1/WC2 complex directly binds the Sre1 promoter and activates its expression (Figure 6). This regulatory mode has been documented in several other fungi, albeit with different downstream partners. In *N. crassa* , the WC complex directly targets the central clock component frequency (*frq*) gene and the light‐responsive gene sub‐1 (Froehlich et al. 2002; Corrochano 2019). In *B. cinerea* , the transcription factor Vel1 acts downstream of BcWCL2 to regulate virulence (Ren et al. 2024), and in *S. commune* , multiple hydrophobins and transcription factors are downstream targets of the WC complex during mushroom development (Ohm et al. 2013; Vonk et al. 2024). Furthermore, WC2 regulates the expression of the phytoene desaturase gene *CrtI* and the astaxanthin synthase gene *CrtS* in *Xanthophyllomyces dendrorhous* (Huang et al. 2022). Our identification of Sre1, a GATA‐type transcription factor itself, as a direct target of WC2 reveals a novel inter‐GATA regulatory cascade. Expressing Sre1 in Δ*WC1* or Δ*WC2* mutants partially restored chlamydospore and trap formation, confirming that Sre1 is a key downstream effector.

Among the Sre1 regulon, we identified genes involved in ergosterol biosynthesis, MAPK signalling, and cell wall biogenesis. This multi‐pronged mechanism simultaneously controls membrane fluidity, stress signalling, and cell wall architecture, providing a molecular explanation for the defects in chlamydospore diameter, stress sensitivity, and trap formation observed in Δ*Sre1* mutants. This finding is consistent with the established role of Sre1 as a master regulator of iron and sterol homeostasis in fungi (Chao et al. 2008), but extends it by demonstrating a direct link to MAPK signalling and carbohydrate metabolism. Notably, the central role of iron acquisition in trap morphogenesis has been recently highlighted in *D. haptotyla*, where the siderophore biosynthetic gene *DhSip1* is essential for adhesive knob formation, and its deletion phenocopies key aspects of our Δ*Sre1* mutant (Lei et al. 2026). Both studies converge on a shared principle: iron‐dependent processes are critical for sustaining the energy‐demanding process of trap development. In *D. haptotyla*, this operates through siderophore‐mediated iron uptake, whereas in *A. flagrans* , it is achieved via Sre1. The authors of that study proposed that iron may support ATP generation and/or modulate MAPK or cAMP‐PKA signalling cascades through ROS homeostasis (Lei et al. 2026), which aligns precisely with our identification of MAPK components as Sre1 targets.

### 4.5 Limitations, Translational Potential and Future Challenges

We acknowledge several limitations of the current study that also point toward important future directions. First, while our integrative RNA‐seq and DAP‐seq analyses identified a substantial set of putative downstream targets of WC2, functional validation was largely confined to Sre1. Dozens of other candidate genes await systematic characterization. A more comprehensive gene regulatory network will require targeted deletion and phenotypic analysis of these additional candidates. Second, the negative regulatory roles of Ams2 and NsdD in virulence are inferred primarily from transcriptional correlation and comparative expression analyses. Direct evidence, such as promoter binding or biochemical interaction, is still lacking and requires further validation. Third, while our omics and RT‐qPCR analyses have preliminarily identified several downstream genes of Sre1 that may contribute to chlamydospore formation, it remains unclear whether Sre1 directly binds to their promoters or acts through intermediate transcription factors. Furthermore, except for Swi6 and chitin synthase Chs2, whose roles in chlamydospore formation have already been established, the individual contribution of the remaining target genes to chlamydospore diameter, number, and trap formation has not yet been experimentally dissected.

Despite these limitations, the candidate targets and regulatory cascades defined in this work provide a foundation for both mechanistic follow‐up and translational application. From an applied perspective, our work defines WC2 and Sre1 as actionable molecular targets for engineering superior biocontrol strains. We speculate that combining *WC2* overexpression with deletion of *Ams2* or *NsdD* might enhance trap formation, chlamydospore production, and nematicidal efficacy. However, we emphasize that such engineered strains have not yet been constructed or validated, and translating these genetic modifications into field‐applicable products requires addressing several practical challenges. As highlighted in recent analyses of microbial biocontrol agent development, the gap between laboratory efficacy and field performance remains substantial (Belt et al. 2026). Key hurdles include optimizing formulation for environmental stability, ensuring consistent performance across soil types and climatic conditions, and meeting regulatory requirements for genetically modified organisms. Future work will focus on creating and rigorously evaluating such engineered strains in microcosm and field conditions to translate these fundamental insights into a next‐generation bionematicide for the sustainable management of PPNs.

## 5 Conclusions

In conclusion, this study provides a systematic functional atlas of GATA‐type transcription factors in a nematode‐trapping fungus. We demonstrate that these GATA‐type transcription factors form a sophisticated regulatory network governing growth, stress response, pathogenicity, and chlamydospore formation. Our findings elucidate a key regulatory cascade for predatory and survival structures: the WC1/WC2 complex promotes chlamydospore and trap formation by directly inducing the expression of *Sre1* (Figure 8). This work significantly advances our understanding of the molecular mechanisms underlying fungal predation and development. The identified key regulators, particularly WC1, WC2, Sre1, Ams2, and NsdD, represent potential targets for enhancing the biocontrol efficacy of *A. flagrans* and related fungi against parasitic nematodes.

## Author Contributions

**Hanbo Zhang:** investigation, formal analysis, software. **Jiafang Zuo:** methodology, investigation, data curation, supervision. **Peiji Zhao:** methodology, software, visualization. **Minghe Mo:** investigation, methodology, software. **Qianfei Shi:** software, formal analysis, visualization, data curation. **Yu Zhang:** methodology, software, formal analysis, validation, funding acquisition, writing – original draft. **Guohong Li:** writing – review and editing, project administration, resources, conceptualization, supervision, funding acquisition.

## Funding

This research was supported by the National Key Research and Development Program (2023YFD1400400), Science and Technology Innovation Base Construction Project (202603AS090012, 202407AB110004), Projects from the Department of Science and Technology of Yunnan Province (202501AS070058, 202401BC070010, 202301BC070017), and the China Postdoctoral Science Foundation under Grant Number 2025M782758.

## Disclosure

The authors declare that no generative artificial intelligence or AI‐assisted technologies were used in the research or writing of this manuscript. All content was written, reviewed, and revised entirely by the authors, who take full responsibility for the accuracy, originality, and integrity of the work.

## Ethics Statement

The authors have nothing to report.

## Conflicts of Interest

The authors declare no conflicts of interest.

## Supporting information


**Figure S1:** Conserved domain analysis of GATA‐type transcription factors in *A. flagrans* .
**Figure S2:** Phylogenetic tree of eight GATA‐type transcription factor orthologs from different fungi using the neighbour‐joining method.
**Figure S3:** Expression profiles of eight GATA‐type transcription factors during chlamydospore and trap formation.
**Figure S4:** Targeted gene deletion of Sre1.
**Figure S5:** Targeted gene deletion of GATA1.
**Figure S6:** Targeted gene deletion of ASD4.
**Figure S7:** Targeted gene deletion of WC2.
**Figure S8:** Targeted gene deletion of AreA.
**Figure S9:** Targeted gene deletion of Ams2.
**Figure S10:** Targeted gene deletion of NsdD.
**Figure S11:** Targeted gene deletion of WC1.
**Figure S12:** Validation of gene complementation and analysis of mycelial growth in the complementation strains.
**Figure S13:** Comparison of hyphal growth between WT and eight GATA‐type transcription factor mutants.
**Figure S14:** Detection of hyperosmotic stress between WT and eight GATA‐type transcription factor mutant strains.
**Figure S15:** Detection of cell wall integrity between WT and eight GATA‐type transcription factor mutant strains.
**Figure S16:** Detection of oxidative stress between WT and eight GATA‐type transcription factor mutant strains.
**Figure S17:** Relative growth inhibition (RGI) of fungal colonies.
**Figure S18:** Analysis of the trap formation and pathogenicity in complementation strains of the eight GATA‑type transcription factors.
**Figure S19:** Analysis of extracellular protease activity.
**Figure S20:** Analysis of the chlamydospore formation in complementation strains of the eight GATA‑type transcription factors.
**Figure S21:** Analysis of the effect of light on chlamydospore formation.
**Figure S22:** RNA‐seq analysis of WT and ΔWC2 mutant strains in chlamydospore formation.
**Figure S23:** KEGG and GO annotation analysis of WC2 target genes.
**Figure S24:** Analysis of expression level of the other seven GATA‐type transcription factors in the ΔWC2 mutant strain.
**Figure S25:** RT‐qPCR analysis of the expression levels of trap formation‐related genes and virulence factors in the ΔWC2 mutants.
**Figure S26:** Analysis of the effect of the global regulator LaeA on the expression of eight GATA‐type transcription factors.
**Figure S27:** Effect of WC2 on the expression of chlamydospore formation‐related genes.
**Table S1:** A. flagrans strains and plasmids used in this study.
**Table S2:** Primers used in this study.
**Table S3:** The predicted and tallied physiochemical properties of eight GATA proteins in A. flagrans.
**Table S4:** Per‑trap killing efficiency at 12 and 24 h post‑nematode addition.
**Table S5:** Protein details in the protein–protein interaction network predicted by STRING.
**Table S6:** Downstream target genes of WC2 identified through integrated RNA‐seq and DAP‐seq analyses in chlamydospore formation.
**Table S7:** qPCR analysis to validate the series of potential genes regulated by Sre1.

## Data Availability

The *Arthrobotrys flagrans* genome database used in this study is available at the National Center for Biotechnology Information (NCBI) GenBank under the accession number PRJNA917252. The RNA‐seq data presented here are associated with NCBI BioProject PRJNA1310194 and BioSample SAMN50770116. The DAP‐seq data presented here are associated with NCBI BioProject PRJNA1455353. All other data are available from the corresponding author (or other sources, as applicable) on reasonable request.
