# Supplementary material for: WC1/WC2–Sre1 Transcriptional Cascade Controls Predation and Chlamydospore Formation in the Nematode‐Trapping Fungus Arthrobotrys flagrans
Source: Microb Biotechnol. 2026 Jul 9;19(7):e70412. doi: 10.1111/1751-7915.70412 (PMC13351115; doi:10.1111/1751-7915.70412)
Supplement: Supplementary file 1 — Figure S1: Conserved domain analysis of GATA‐type transcription factors in A. flagrans . Figure S2: Phylogenetic tree of eight GATA‐type transcription factor orthologs from different fungi using the neighbour‐joining method. Figure S3: Expression profiles of eight GATA‐type transcription factors during chlamydospore and trap formation. Figure S4: Targeted gene deletion of Sre1. Figure S5: Targeted gene deletion of GATA1. Figure S6: Targeted gene deletion of ASD4. Figure S7: Targeted gene deletion of WC2. Figure S8: Targeted gene deletion of AreA. Figure S9: Targeted gene deletion of Ams2. Figure S10: Targeted gene deletion of NsdD. Figure S11: Targeted gene deletion of WC1. Figure S12: Validation of gene complementation and analysis of mycelial growth in the complementation strains. Figure S13: Comparison of hyphal growth between WT and eight GATA‐type transcription factor mutants. Figure S14: Detection of hyperosmotic stress between WT and eight GATA‐type transcription factor mutant strains. Figure S15: Detection of cell wall integrity between WT and eight GATA‐type transcription factor mutant strains. Figure S16: Detection of oxidative stress between WT and eight GATA‐type transcription factor mutant strains. Figure S17: Relative growth inhibition (RGI) of fungal colonies. Figure S18: Analysis of the trap formation and pathogenicity in complementation strains of the eight GATA‑type transcription factors. Figure S19: Analysis of extracellular protease activity. Figure S20: Analysis of the chlamydospore formation in complementation strains of the eight GATA‑type transcription factors. Figure S21: Analysis of the effect of light on chlamydospore formation. Figure S22: RNA‐seq analysis of WT and ΔWC2 mutant strains in chlamydospore formation. Figure S23: KEGG and GO annotation analysis of WC2 target genes. Figure S24: Analysis of expression level of the other seven GATA‐type transcription factors in the ΔWC2 mutant strain. Figure S25: RT‐qPCR analysis of the expressio [file MBT2-19-e70412-s001.docx]

Supplementary information

# **WC1/WC2–Sre1 transcriptional cascade controls predation and chlamydospore formation in the nematode-trapping fungus *Arthrobotrys flagrans***

Yu Zhang ^a,b,1^, Jiafang Zuo ^a,1^, Qianfei Shi ^a^, Peiji Zhao ^a^, Hanbo Zhang ^a,b^, Minghe Mo ^a^, Guohong Li ^a*^

^a^ State Key Laboratory for Conservation and Utilization of Bio-Resources in Yunnan, Yunnan Key Laboratory of Basic Research and Innovative Application for Green Biological Production, Yunnan University, Kunming 650500, China.

^b^ School of Ecology and Environment, Yunnan University, Kunming 650500, China.

^1^ These authors contributed equally to this article.

*Correspondence:

Guohong Li: E-mail: [ligh@ynu.edu.cn](mailto:ligh@ynu.edu.cn), Tel: 86-0871-65031092

Supplementary Figure

## Figure S1


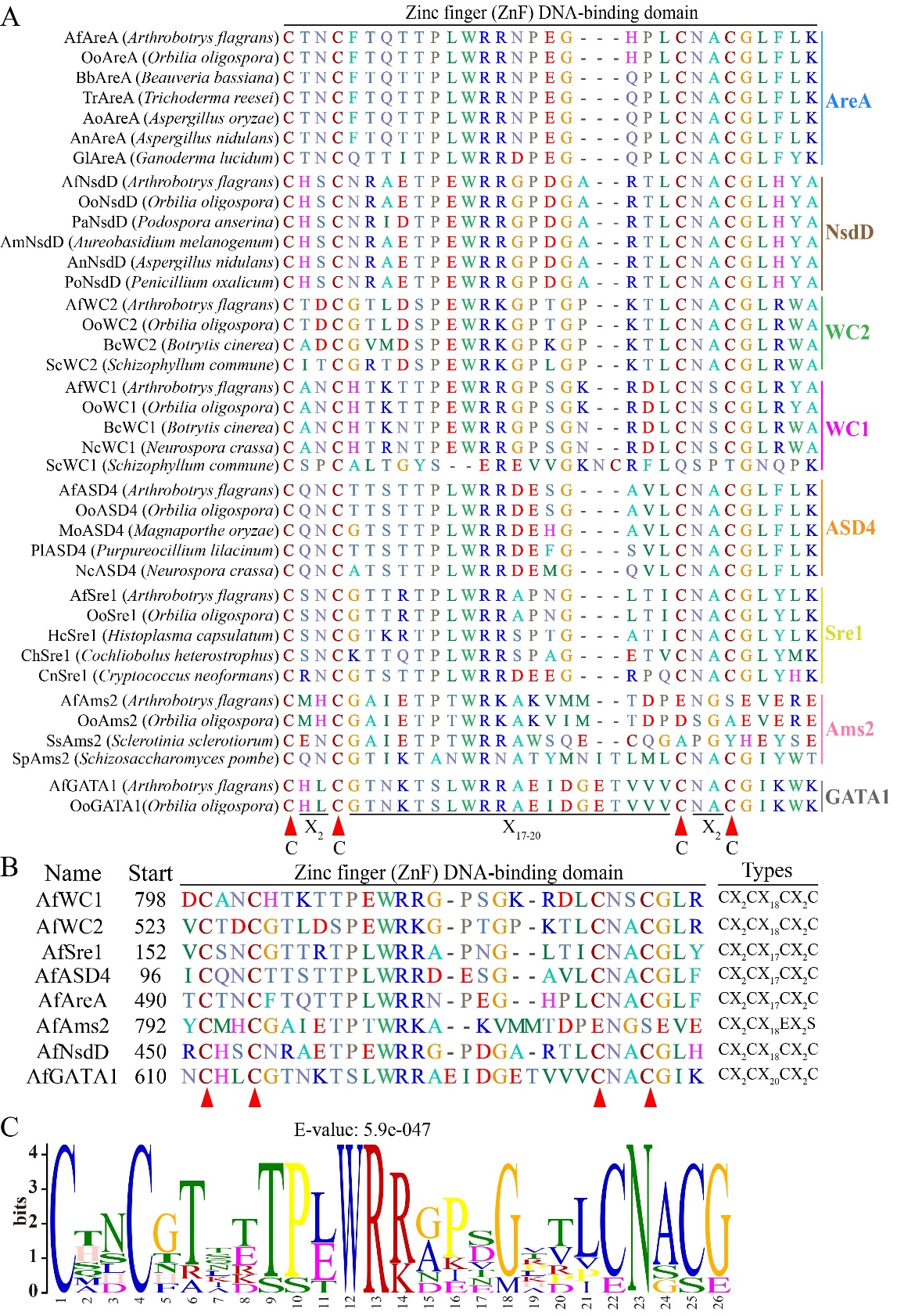


Figure S1. Conserved domain analysis of GATA-type transcription factors in *A. flagrans*.

(A) and (B) Alignment analysis of the conserved amino acid residues within the zinc finger (ZnF) DNA-binding domains from multiple fungi (A) and *A. flagrans* (B) using the BioEdit software. Conserved residues are indicated by red triangles. (C) Motif discovery of conserved amino acid sequences in the GATA-type domain by the MEME software (https://meme-suite.org/meme/tools/meme).

## Figure S2


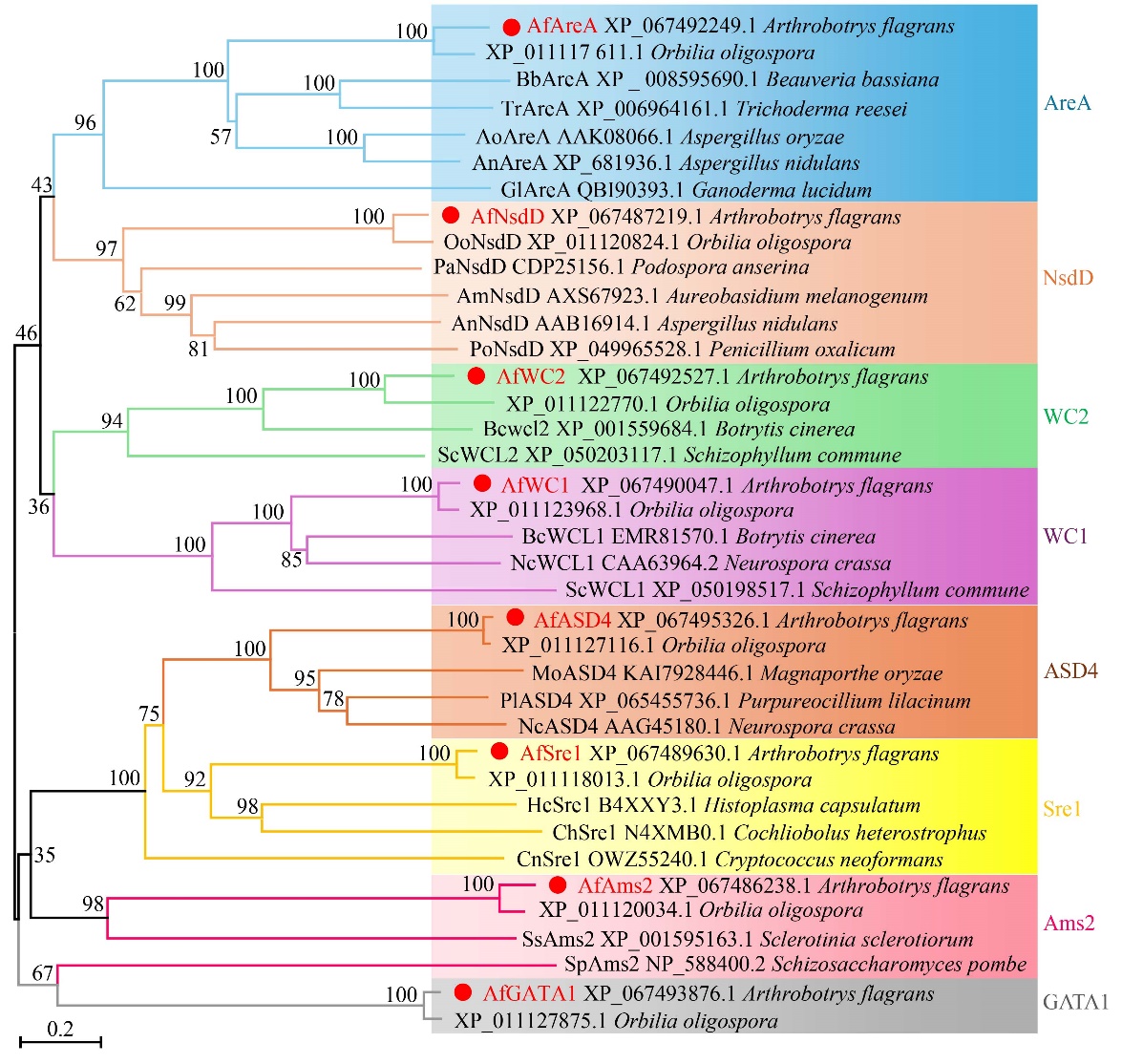


Figure S2. Phylogenetic tree of eight GATA-type transcription factor orthologs from different fungi using the neighbor-joining method.

The numbers represent bootstrap values based on 1000 replicates. All these GATA-type transcription factors from other fungi have been previously reported. The GenBank accession numbers were indicated before the species names.

## Figure S3


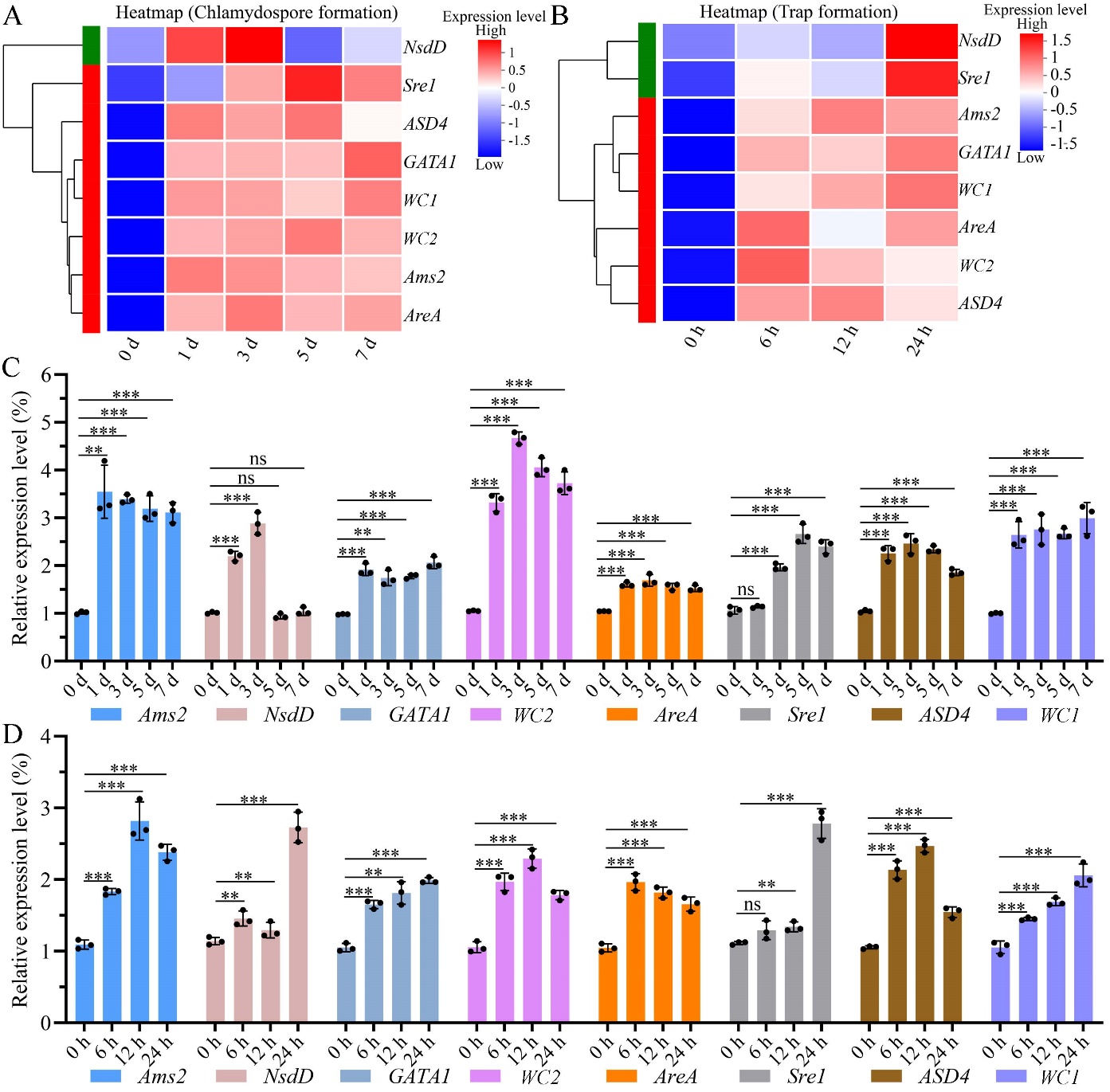


Figure S3 Expression profiles of eight GATA-type transcription factors during chlamydospore and trap formation.

(A) and (B) RNA-seq analysis of the expression levels of GATA-type transcription factors during chlamydospore development (A) and trap formation (B). (C) and (D) RT‑qPCR analysis of the expression levels of GATA-type transcription factors during chlamydospore formation (C) and trap formation (D) (Student's *t*-test; **p* < 0.05, ***p* < 0.01, ****p* < 0.001).

## Figure S4


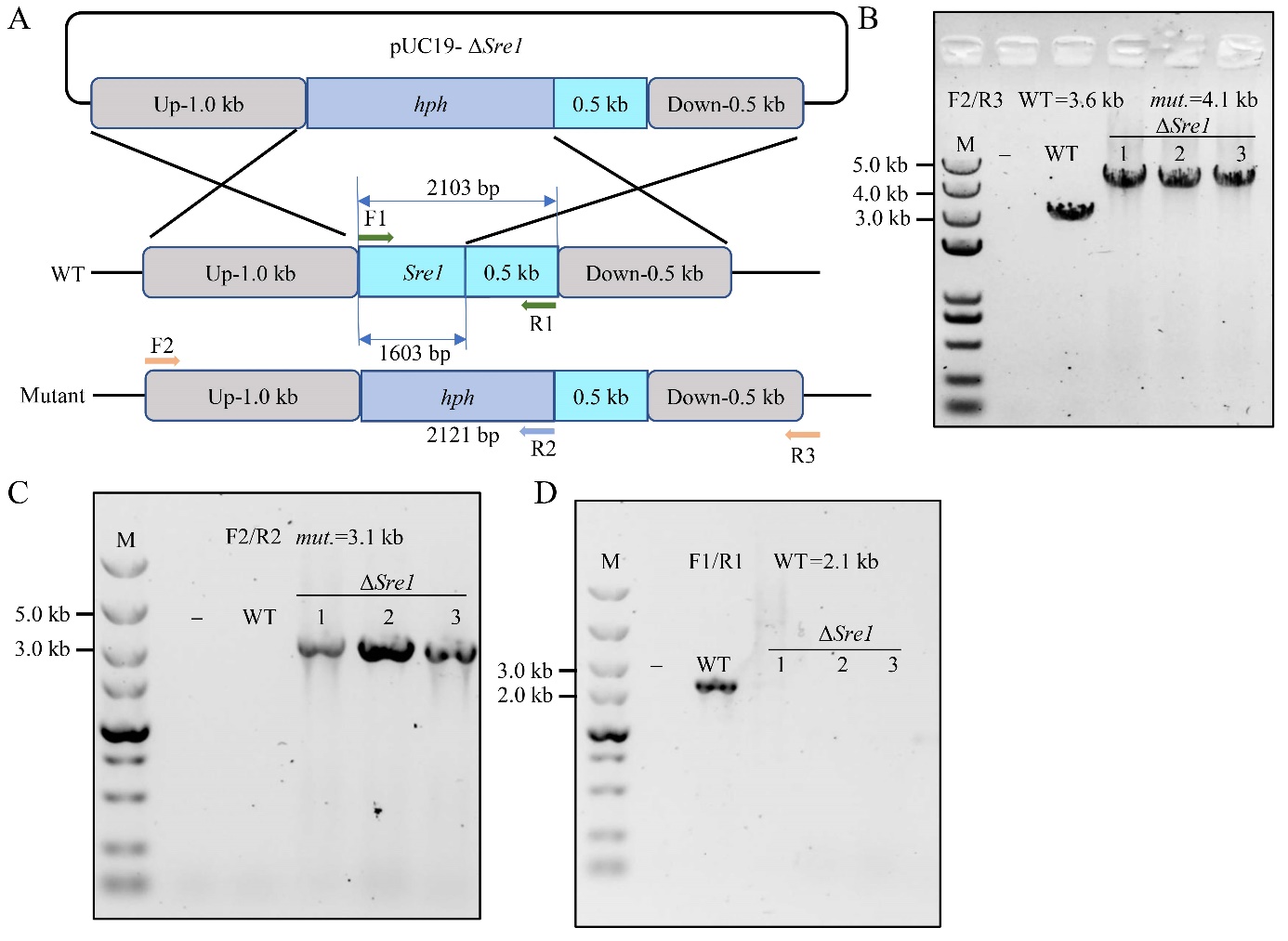


Figure S4. Targeted gene deletion of *Sre1*.

(A) Schematic diagram of the *Sre1* gene deletion. (B–D) PCR detection of differential fragments in the Δ*Sre1* mutants. “-” indicates the negative control without any template in the PCR reaction.

## Figure S5


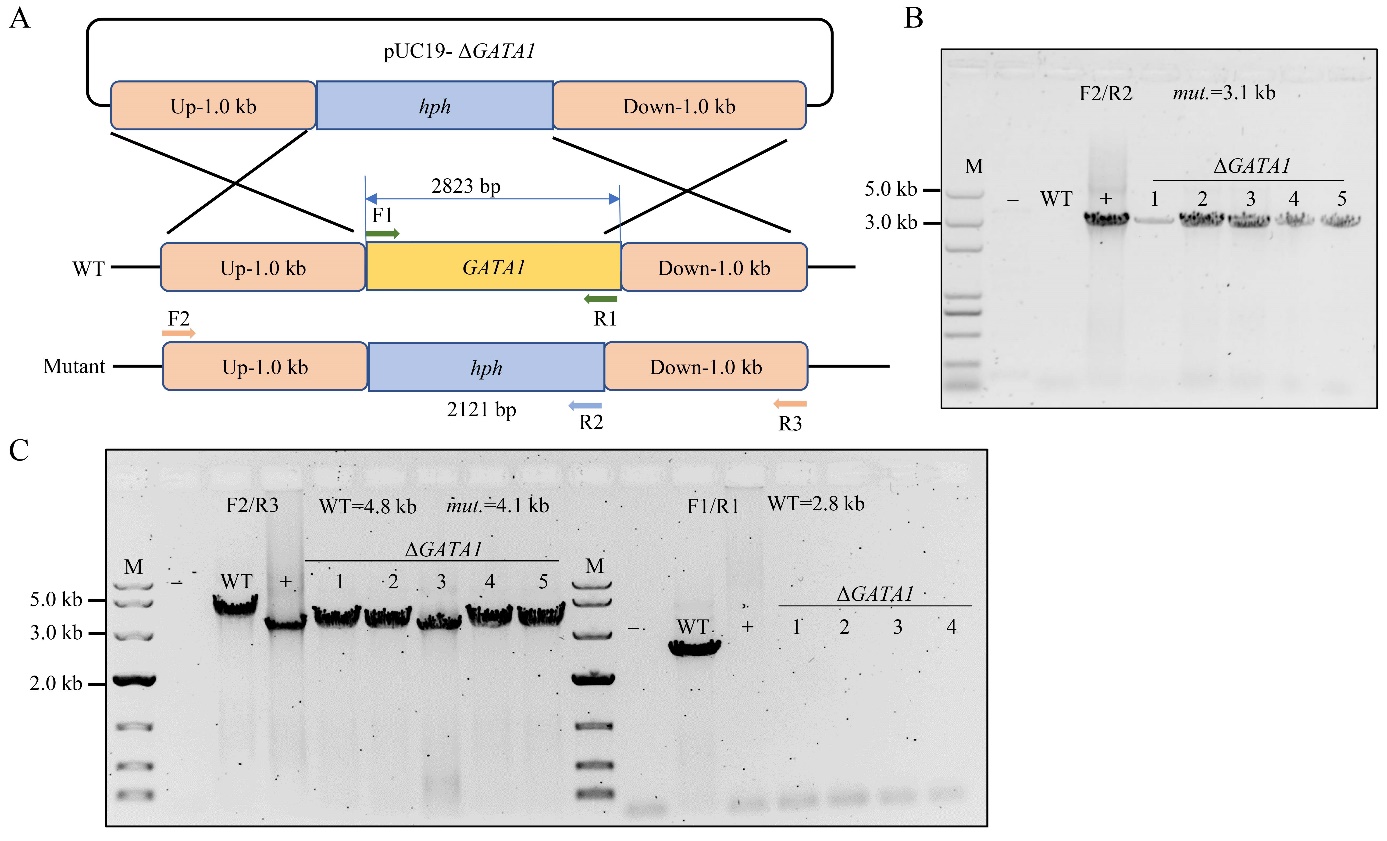


Figure S5 Targeted gene deletion of *GATA1*.

(A) Schematic diagram of the *GATA1* gene deletion. (B) and (C) PCR detection of differential fragments in the Δ*GATA1* mutants. “-” indicates the negative control without any template in the PCR reaction, and “+” indicates the positive control with the *GATA1* knockout plasmid as the template.

## Figure S6


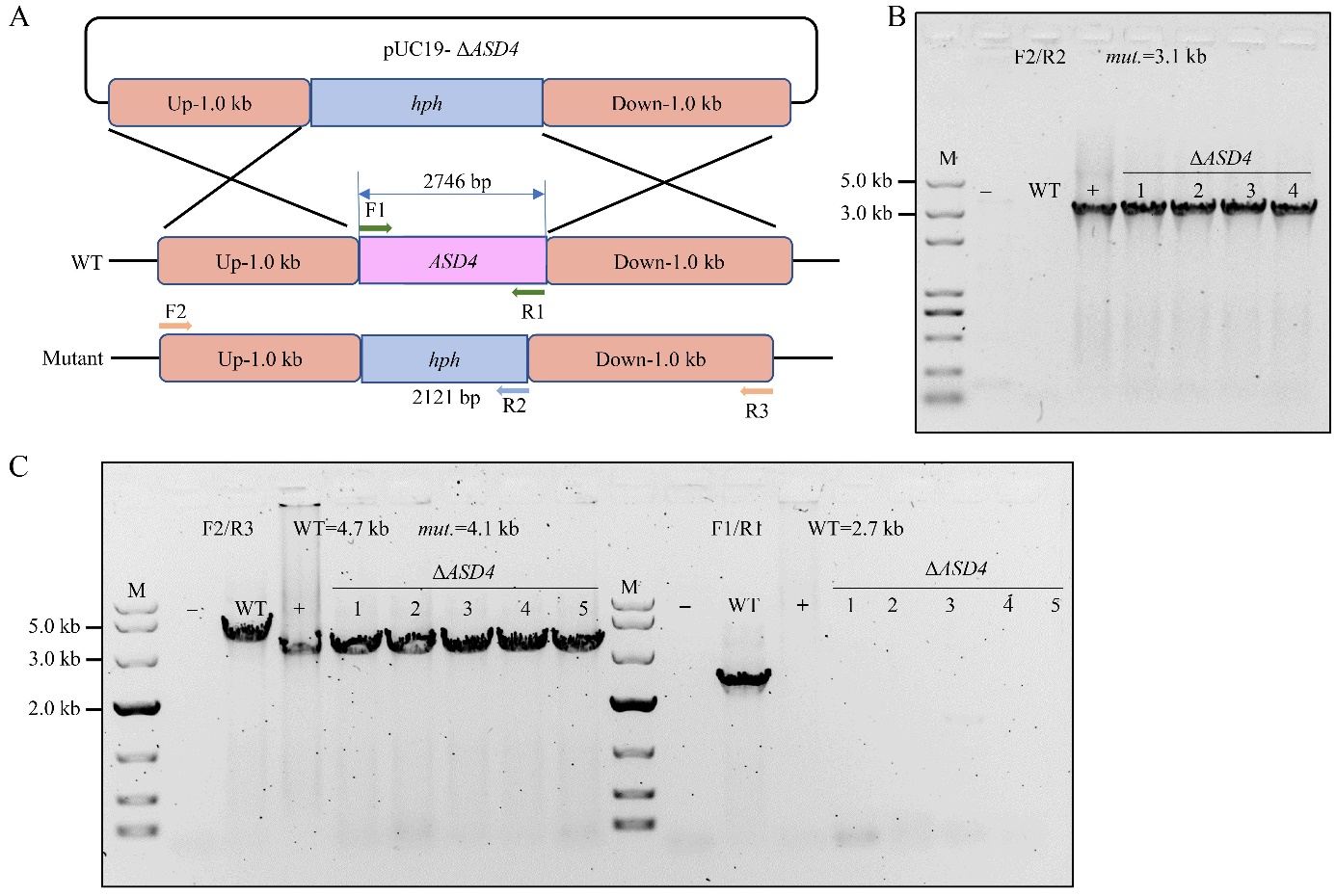


Figure S6 Targeted gene deletion of *ASD4*.

(A) Schematic diagram of the *ASD4* gene deletion. (B) and (C) PCR detection of differential fragments in the Δ*ASD4* mutants. “-” indicates the negative control without any template in the PCR reaction, and “+” indicates the positive control with the *ASD4* knockout plasmid as the template.

## Figure S7


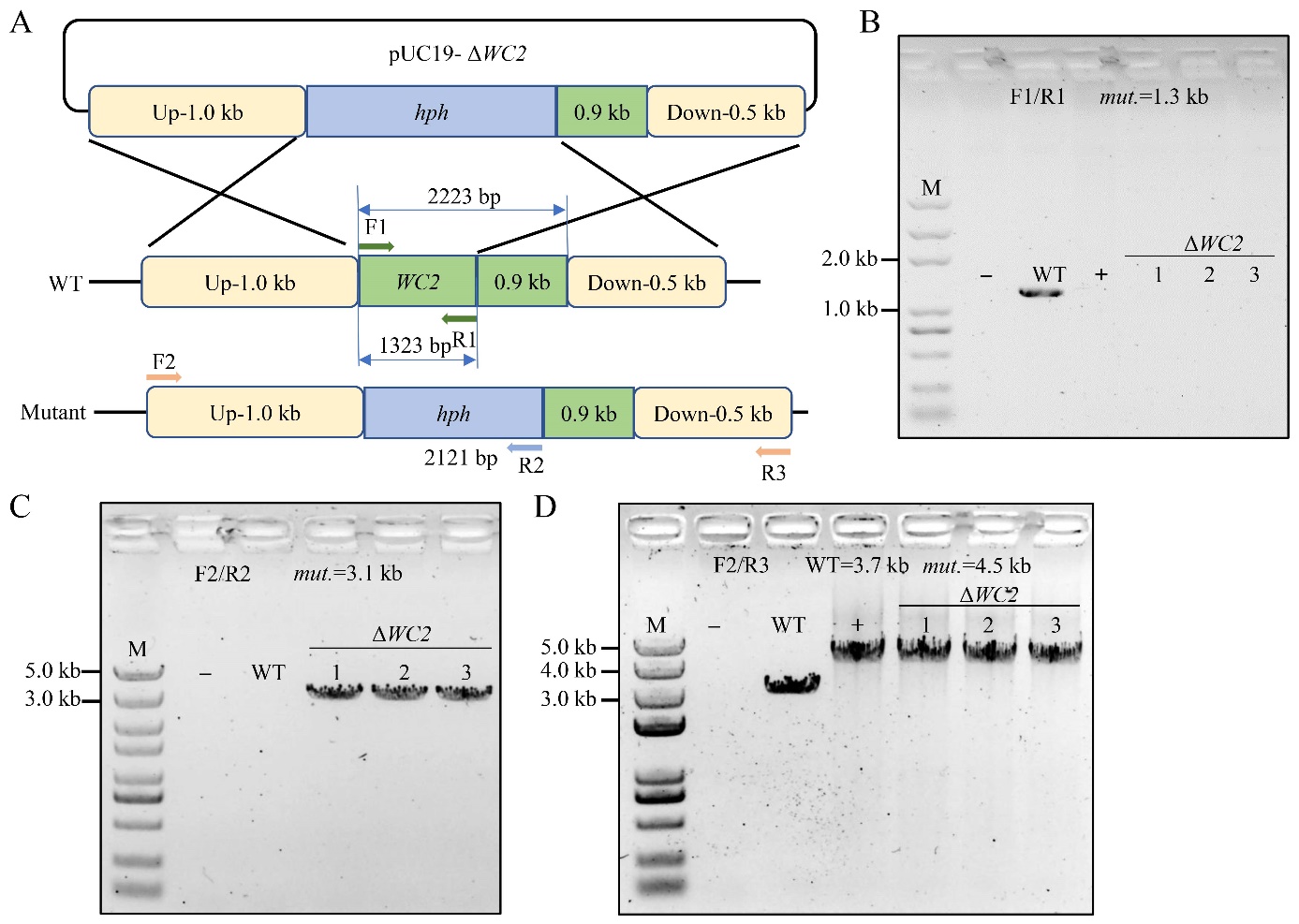


Figure S7 Targeted gene deletion of *WC2*.

(A) Schematic diagram of the *WC2* gene deletion. (B–D) PCR detection of differential fragments in the Δ*WC2* mutants. “-” indicates the negative control without any template in the PCR reaction, and “+” indicates the positive control with the *WC2* knockout plasmid as the template.

## Figure S8


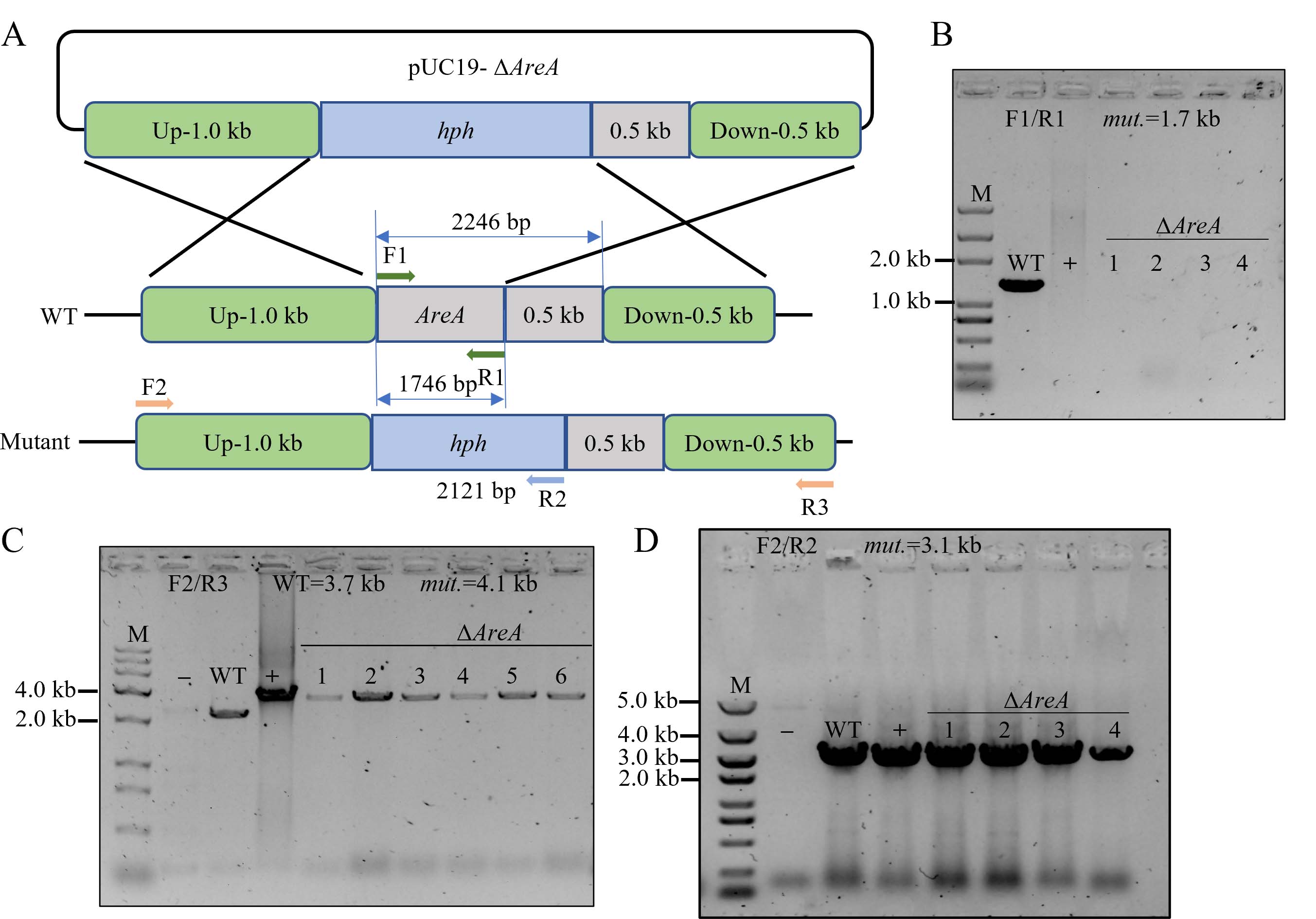


Figure S8 Targeted gene deletion of *AreA*.

(A) Schematic diagram of the *AreA* gene deletion. (B–D) PCR detection of differential fragments in the Δ*AreA* mutants. “-” indicates the negative control without any template in the PCR reaction, and “+” indicates the positive control with the *AreA* knockout plasmid as the template.

## Figure S9


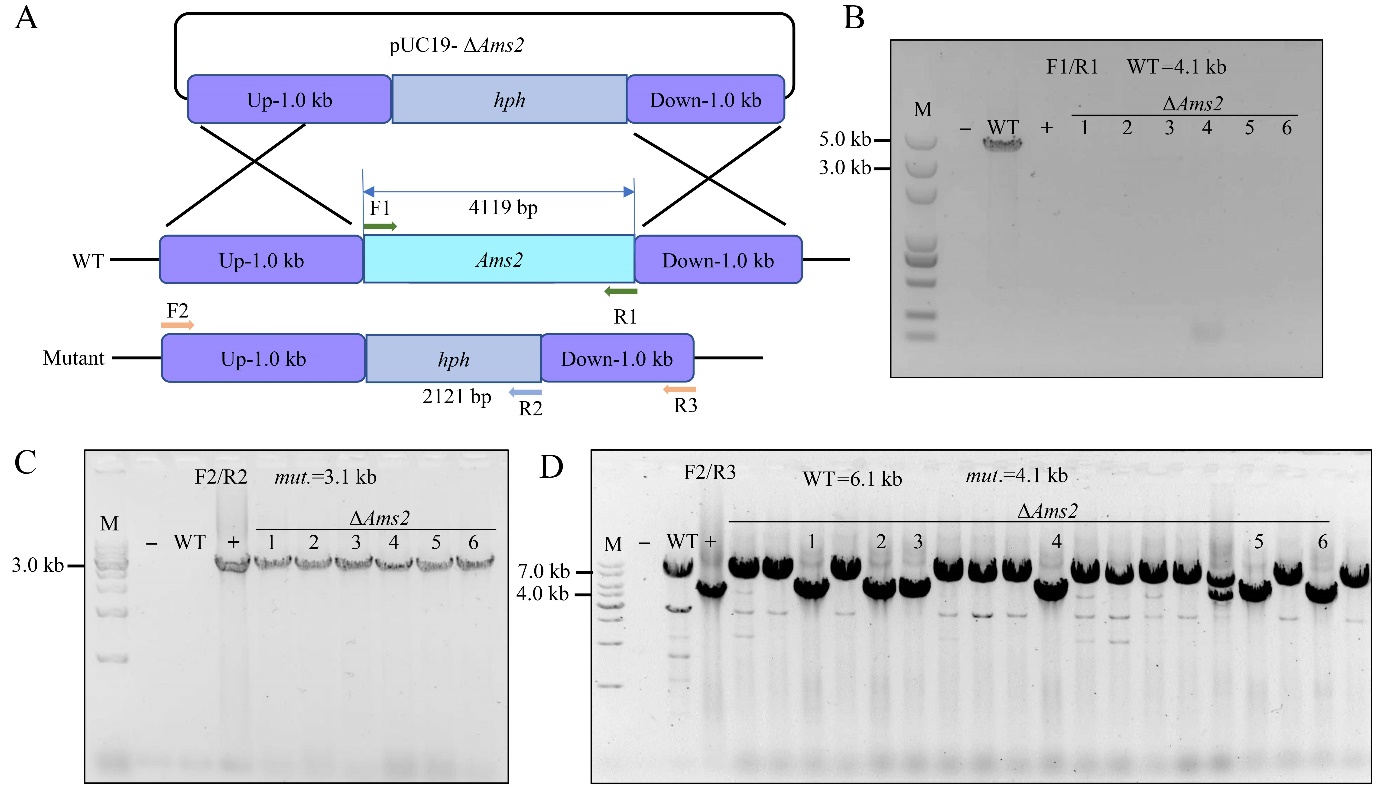


Figure S9 Targeted gene deletion of *Ams2*.

(A) Schematic diagram of the *Ams2* gene deletion. (B–D) PCR detection of differential fragments in the Δ*Ams2* mutants. “-” indicates the negative control without any template in the PCR reaction, and “+” indicates the positive control with the *Ams2* knockout plasmid as the template.

## Figure S10


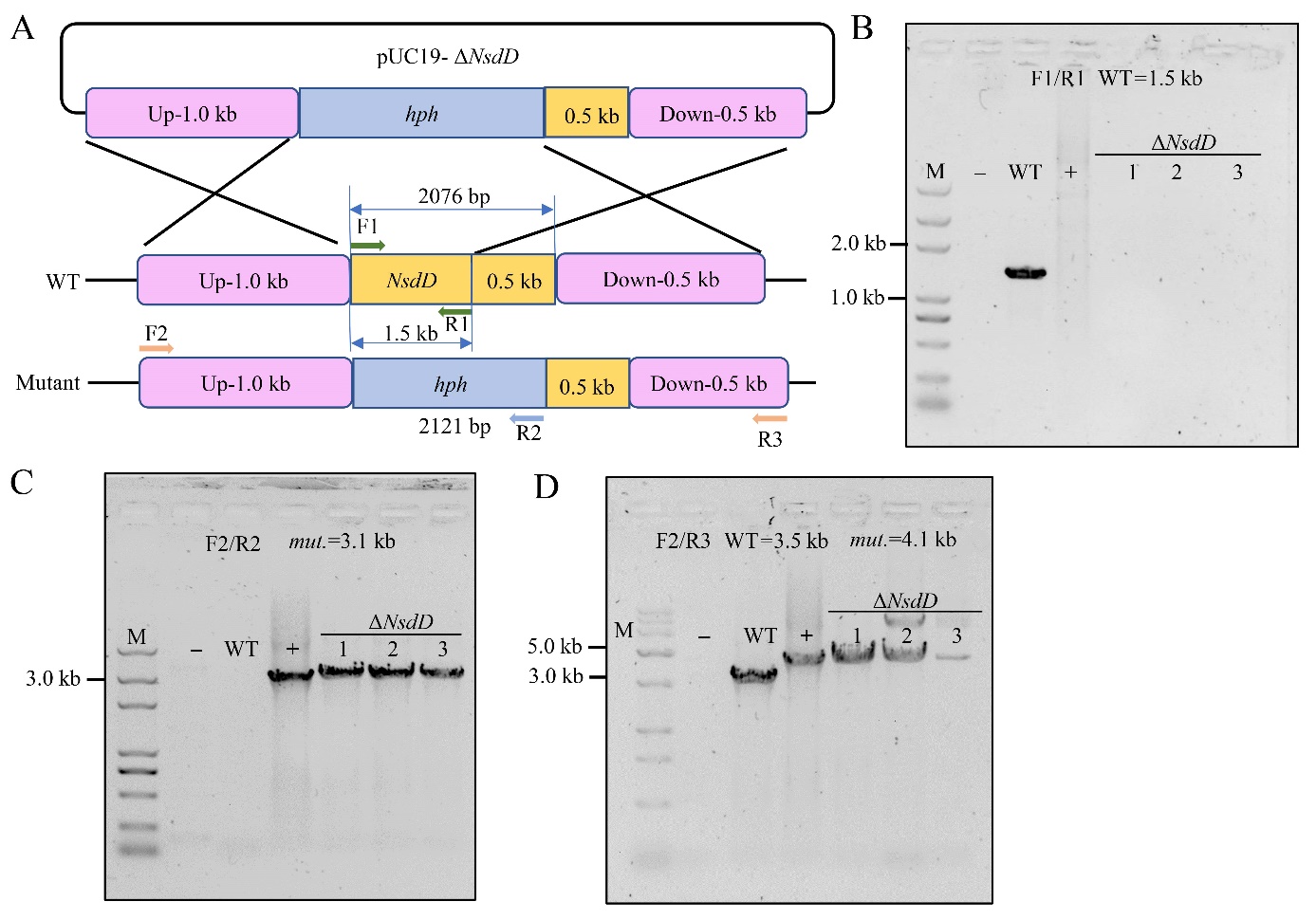


Figure S10 Targeted gene deletion of *NsdD*.

(A) Schematic diagram of the *NsdD* gene deletion. (B–D) PCR detection of differential fragments in the Δ*NsdD* mutants. “-” indicates the negative control without any template in the PCR reaction, and “+” indicates the positive control with the *NsdD* knockout plasmid as the template.

## Figure S11


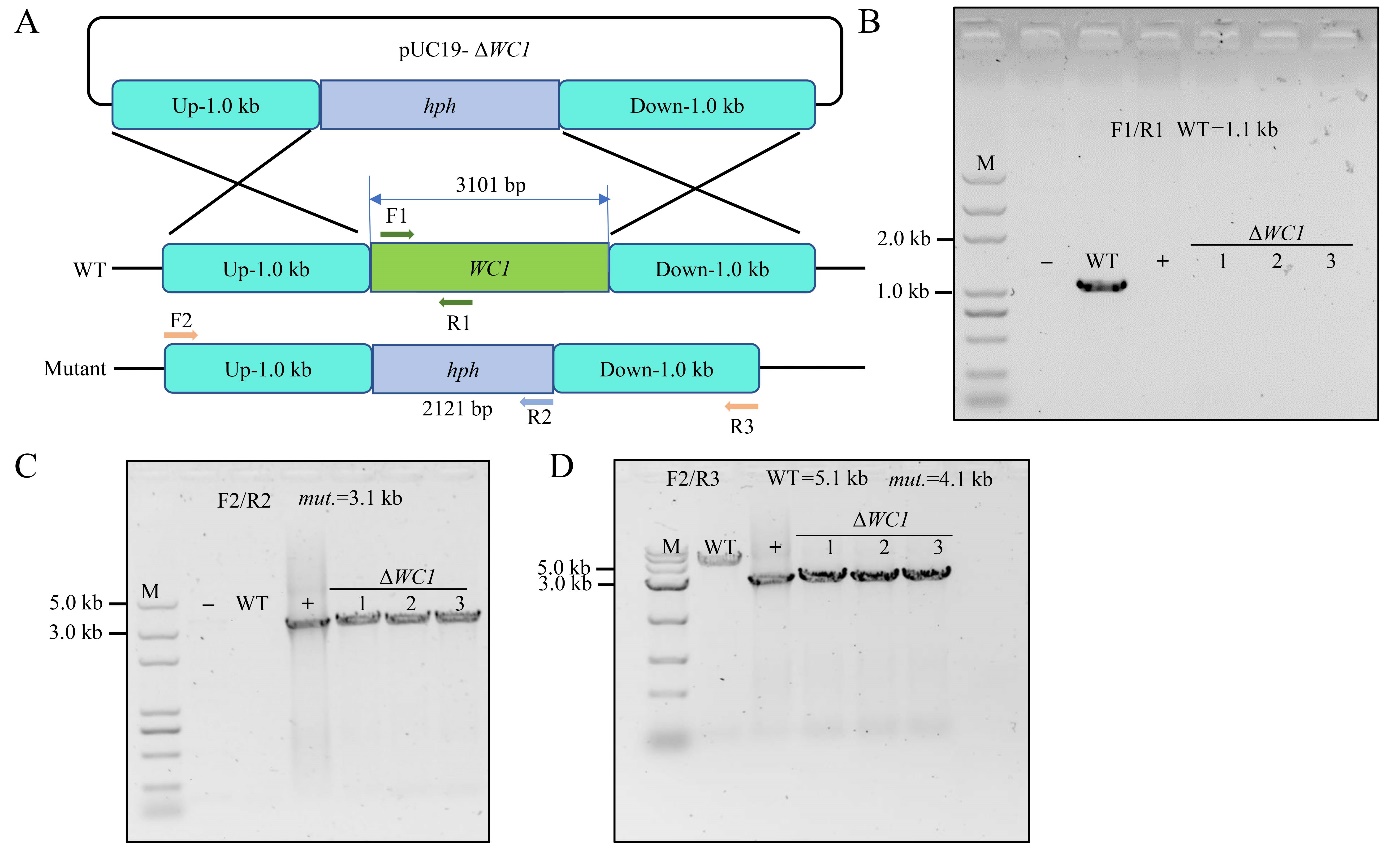


Figure S11 Targeted gene deletion of *WC1*.

(A) Schematic diagram of the *WC1* gene deletion. (B–D) PCR detection of differential fragments in the Δ*WC1* mutants. “-” indicates the negative control without any template in the PCR reaction, and “+” indicates the positive control with the *WC1* knockout plasmid as the template.

## Figure S12


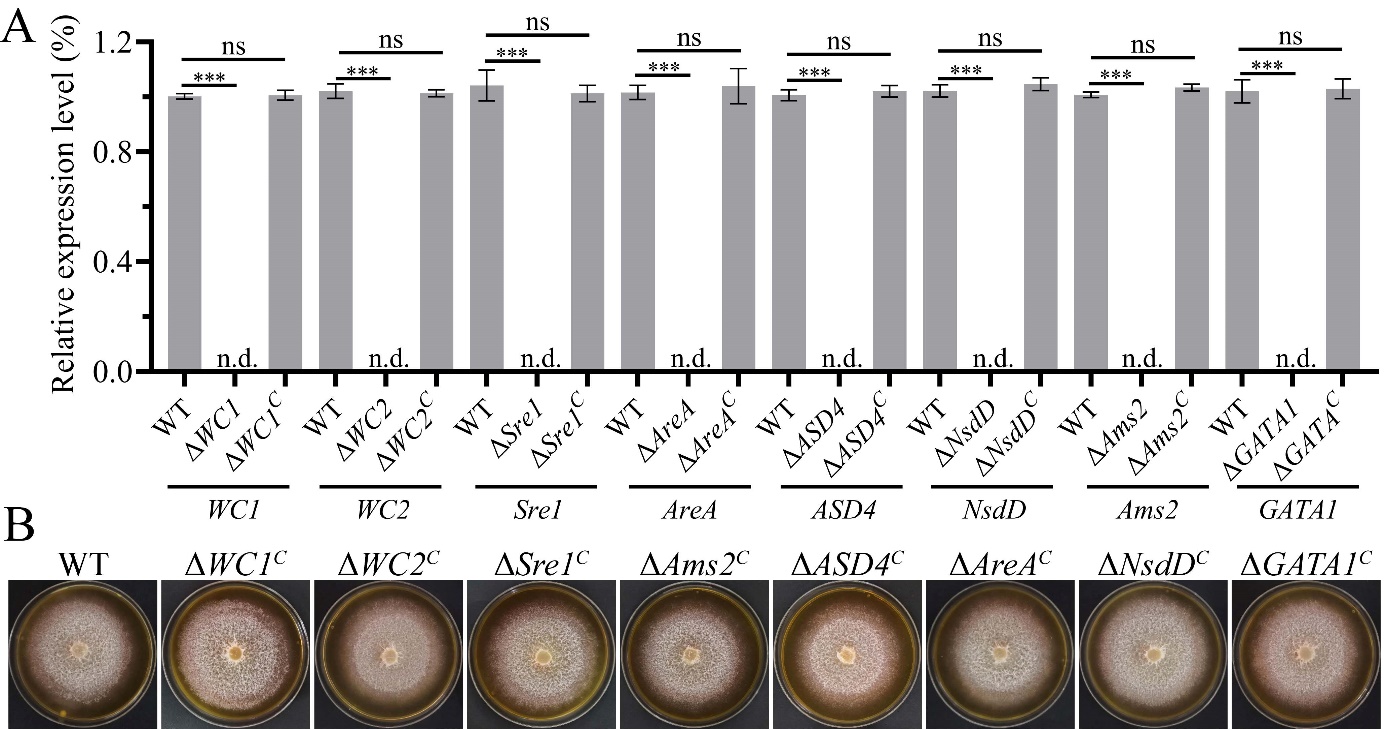


Figure S12 Validation of gene complementation and analysis of mycelial growth in the complementation strains.

(A) RT-qPCR analysis of the relative expression levels of the eight GATA‑type transcription factors in the WT, gene deletion mutants, and complementation strains (Student's *t*-test; ****p* < 0.001). (B) Analysis of the mycelial growth in complementation strains of the eight GATA‑Type transcription factors.

## Figure S13


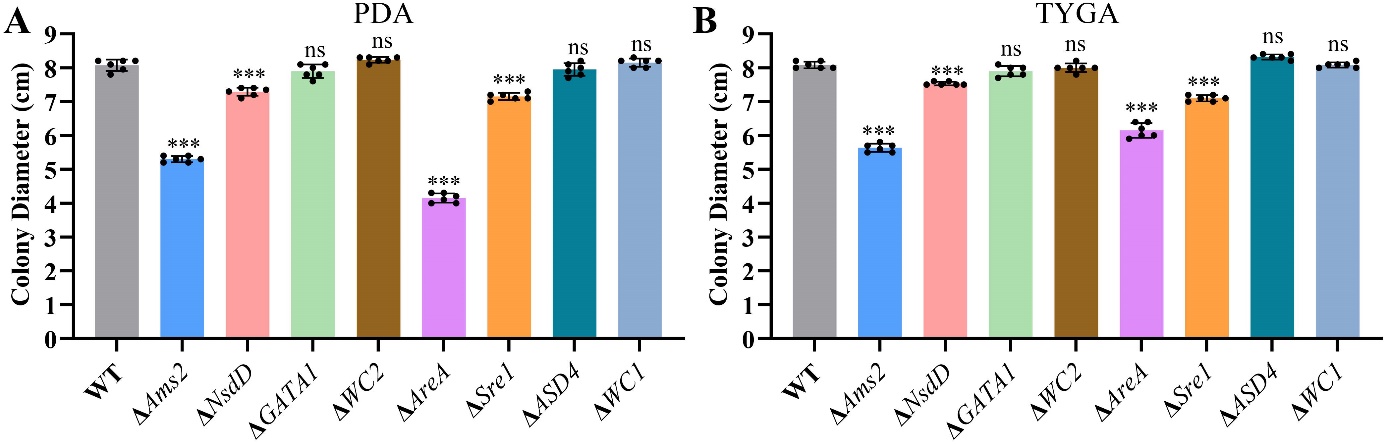


Figure S13 Comparison of hyphal growth between WT and eight GATA-type transcription factor mutants.

Quantification of colony diameter of the indicated strains grown on PDA (A) and TYGA (B) plates (Student's *t*-test; **p* < 0.05, ***p* < 0.01, ****p* < 0.001).

## Figure S14


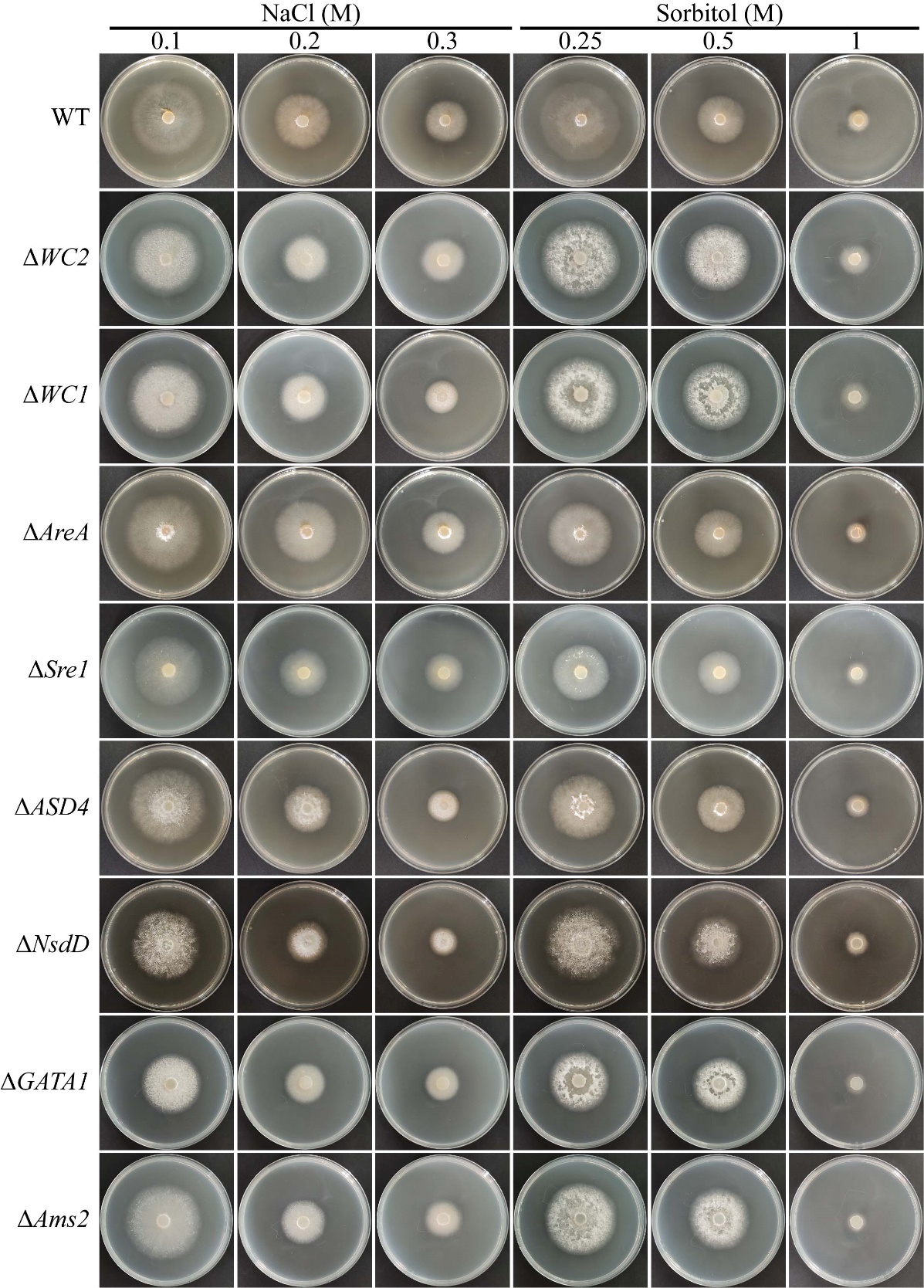


Figure S14 Detection of hyperosmotic stress between WT and eight GATA-type transcription factor mutant strains.

Colony morphology of WT and mutant strains grown on PDA plates containing different osmotic stress agents at 28℃ for 5 days.

## Figure S15


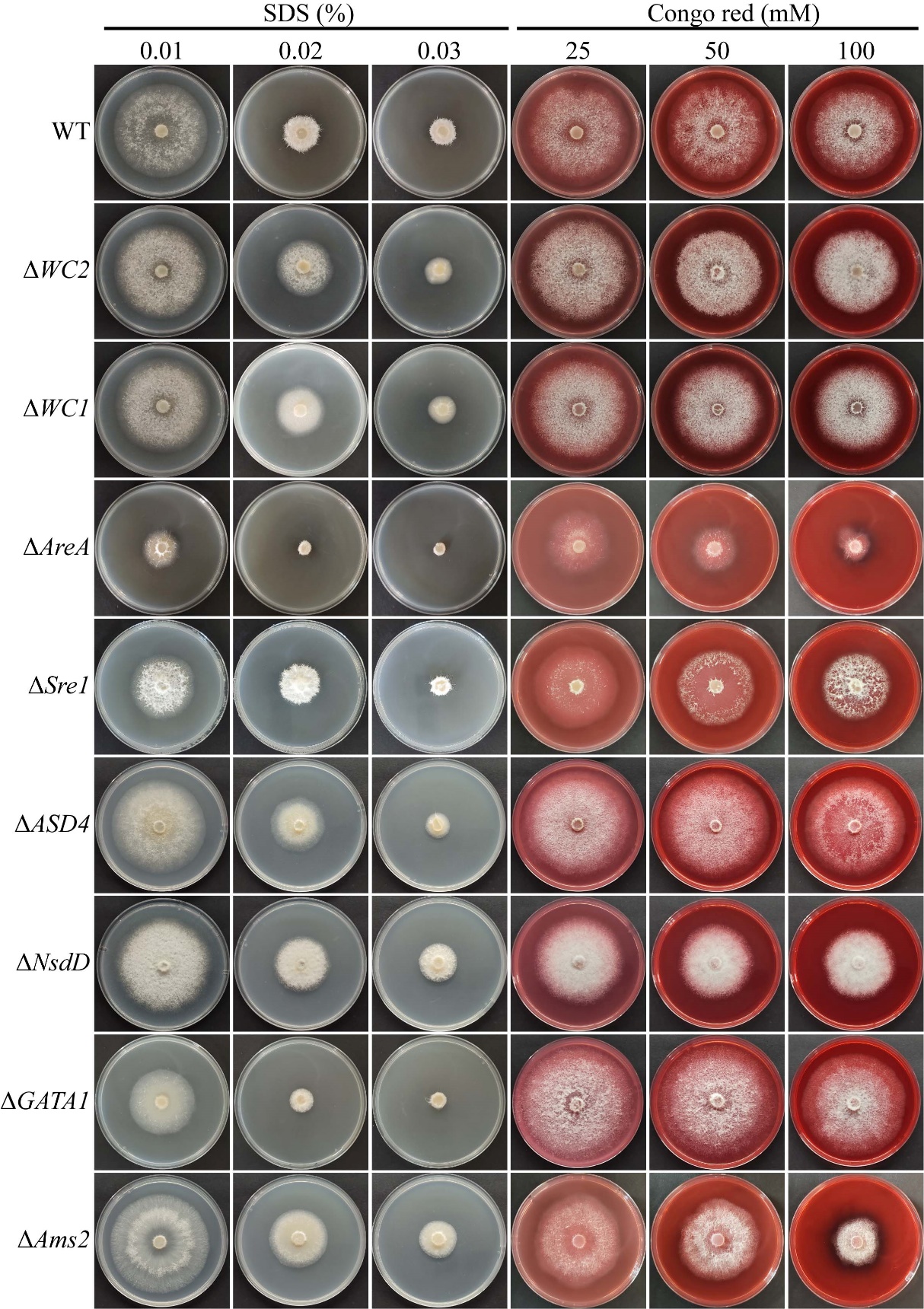


Figure S15 Detection of cell wall integrity between WT and eight GATA-type transcription factor mutant strains.

Colony morphology of WT and mutant strains grown on PDA plates containing different cell wall perturbing reagents at 28℃ for 5 days.

## Figure S16


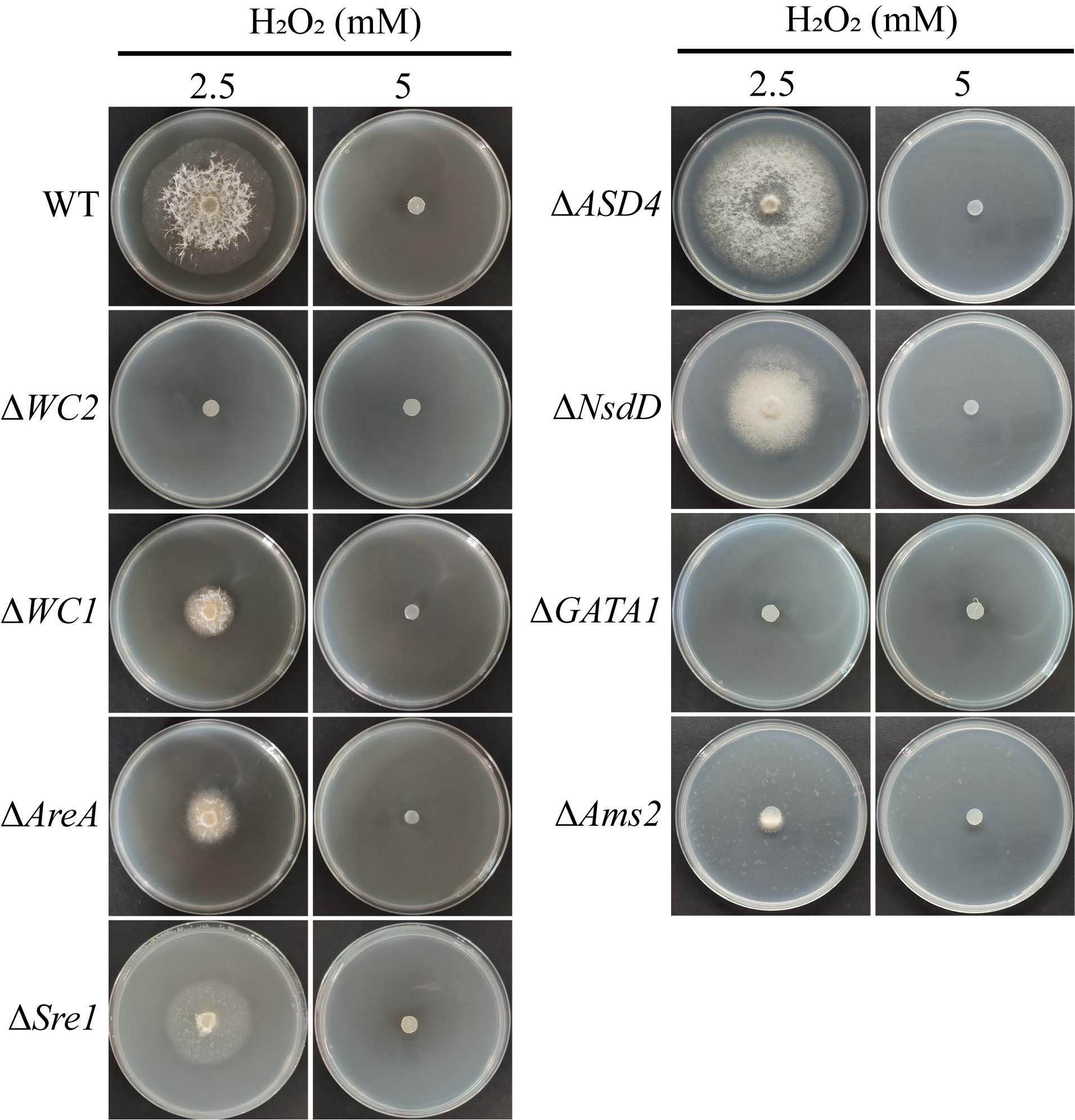


Figure S16 Detection of oxidative stress between WT and eight GATA-type transcription factor mutant strains.

Colony morphology of WT and mutant strains grown on PDA plates containing H_2_O_2_ at 28℃ for 5 days.

## Figure S17


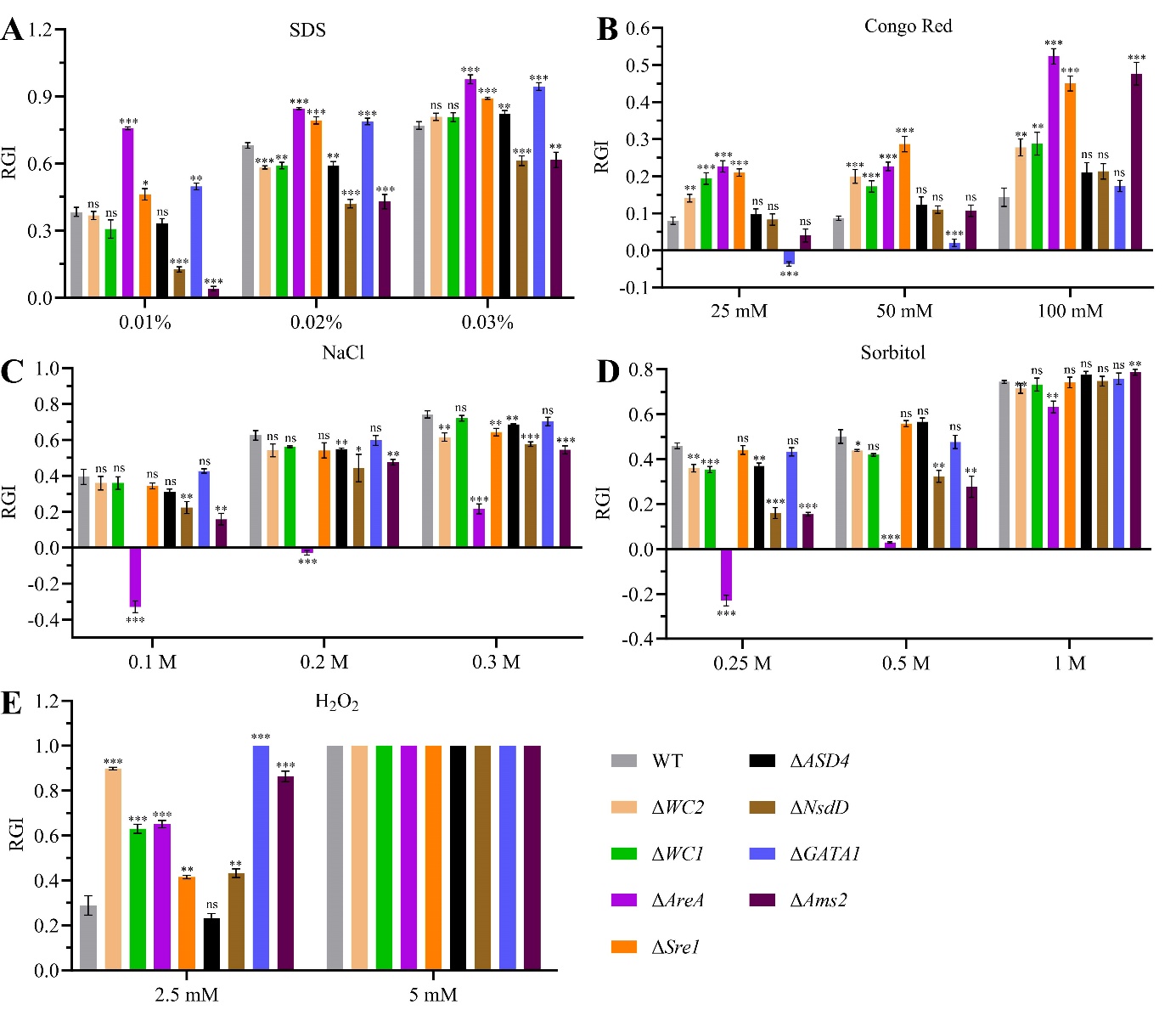


Figure S17 Relative growth inhibition (RGI) of fungal colonies.

RGI values of the WT and eight GATA-type transcription factor mutant strains on medium supplemented with SDS (0.01%, 0.02%, and 0.03%) (A), Congo red (25, 50, and 100 mM) (B), NaCl (0.1, 0.2, and 0.3 M) (C), Sorbitol (0.25, 0.5, and 1.0 M) (D), and H_2_O_2_ (2.5 and 5 mM) (E) (Student's *t*-test; **p* < 0.05, ***p* < 0.01, ****p* < 0.001).

## Figure S18


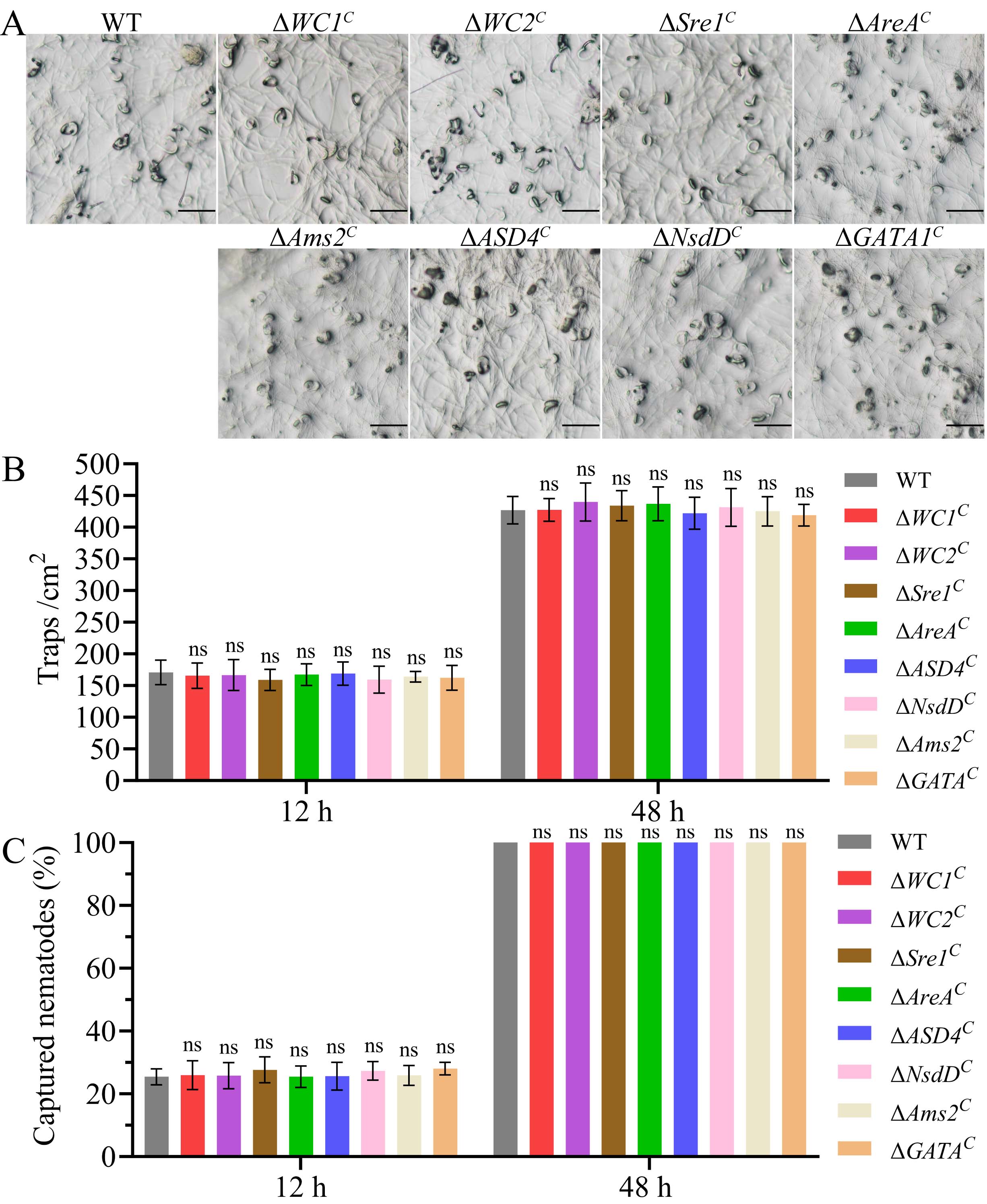


Figure S18 Analysis of the trap formation and pathogenicity in complementation strains of the eight GATA‑type transcription factors.

(A) The representative images of traps at 48 h. Scale bar, 100 μm. (B) and (C) Quantification of the number of traps (B) and captured nematodes (C) by WT and complementation strains at 12 and 48 h (Student's *t*-test; ns: no significant difference).

## Figure S19


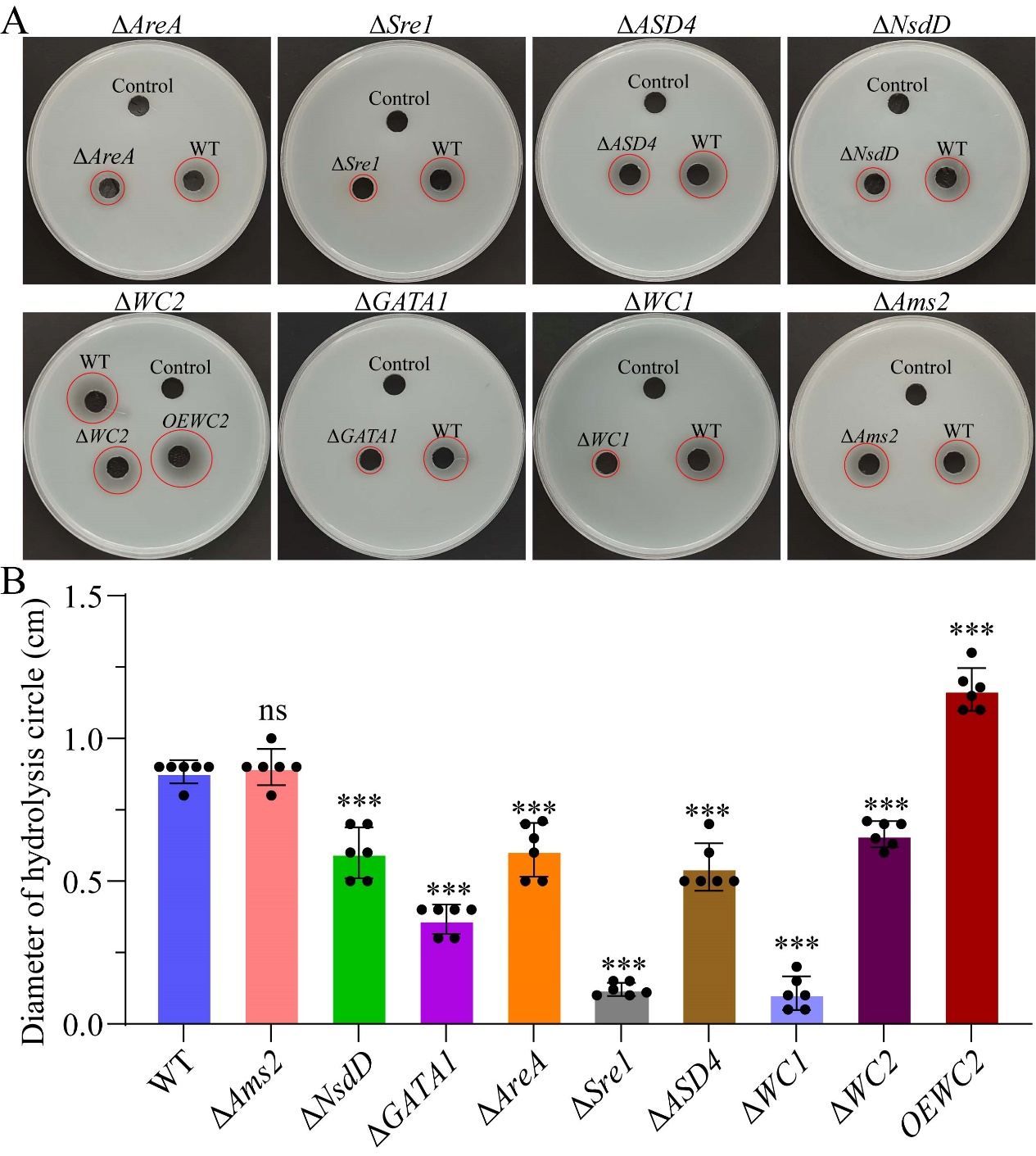


Figure S19 Analysis of extracellular protease activity.

(A) Plate assay showing the extracellular protease activity of eight GATA-type transcription factor mutant strains.

(B) Comparative analysis of the diameter of hydrolysis circles (Student's *t*-test; **p* < 0.05, ***p* < 0.01, ****p* < 0.001).

## Figure S20


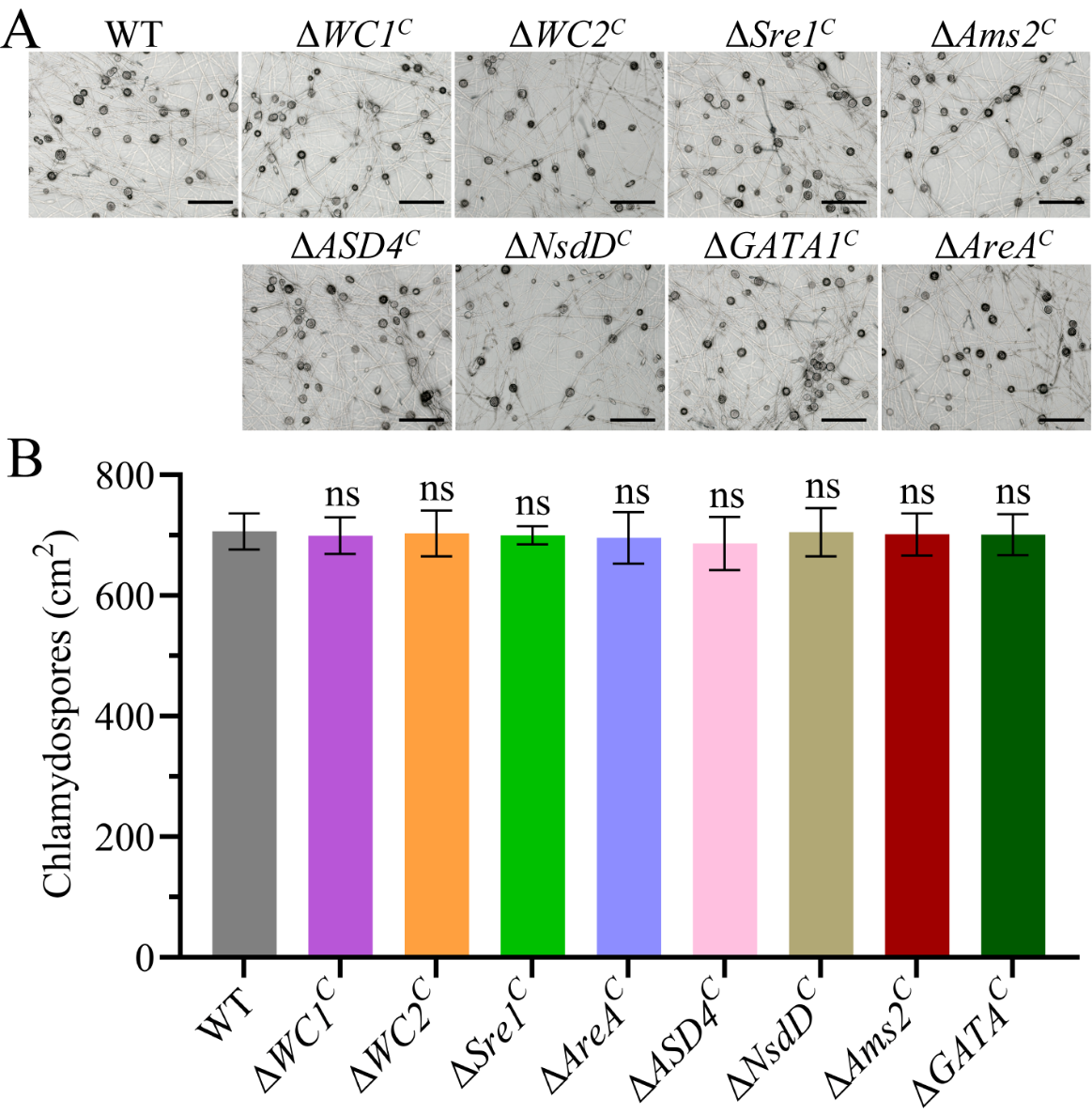


Figure S20 Analysis of the chlamydospore formation in complementation strains of the eight GATA‑type transcription factors.

(A) The morphology of chlamydospores produced by the WT and complementation strains after 14 days of culturing on WA medium. Scale bar, 100 μm. (B) The number of chlamydospores (Student's *t*-test; ns: no significant difference).

## Figure S21


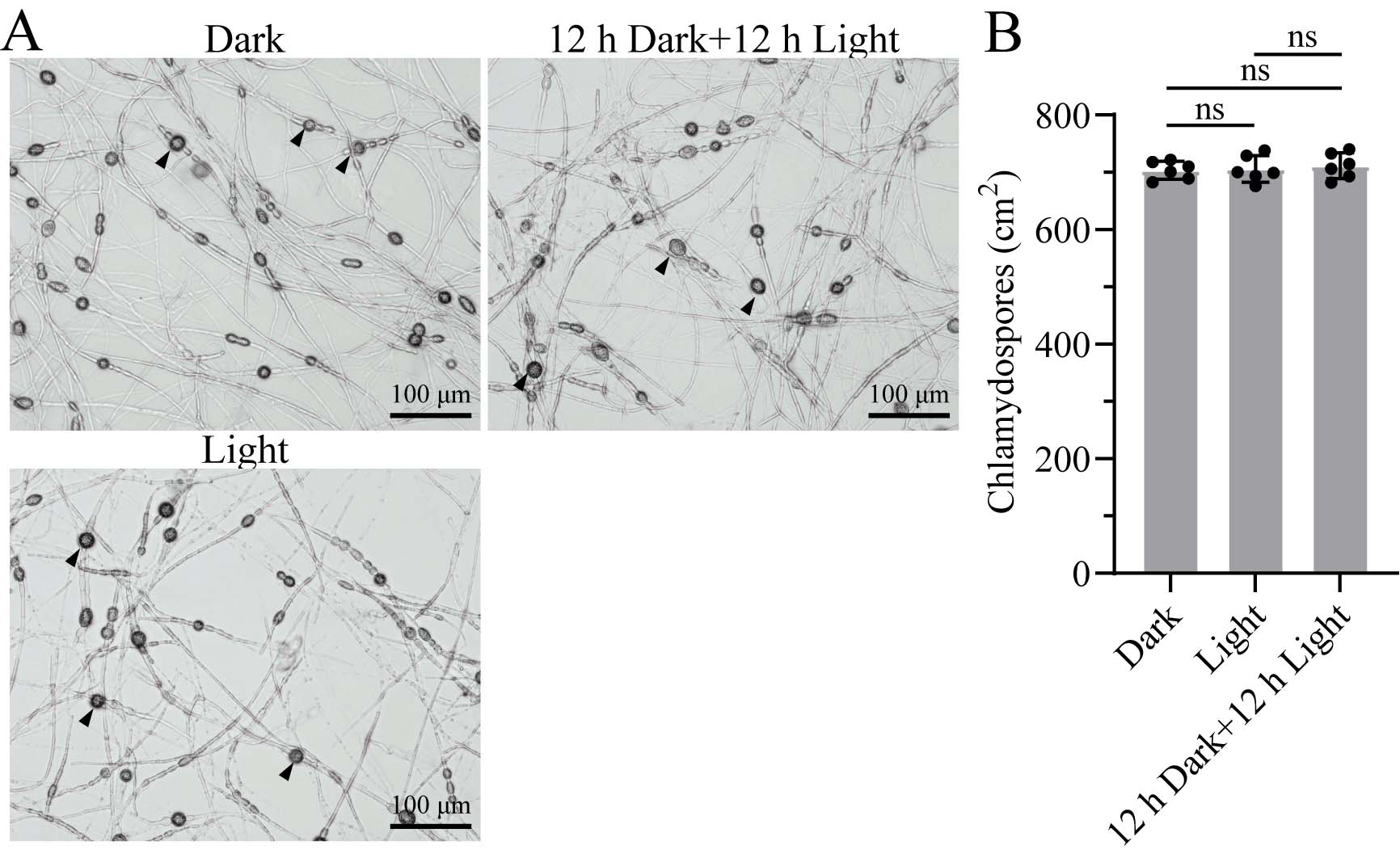


Figure S21 Analysis of the effect of light on chlamydospore formation.

(A) Analysis of chlamydospore formation under continuous darkness, continuous light, and a 12-h light/12-h dark diurnal cycle. (B) Comparison of the number of chlamydospores formed under different durations of light exposure. ns, not significant (Student’s *t*-test).

## Figure S22


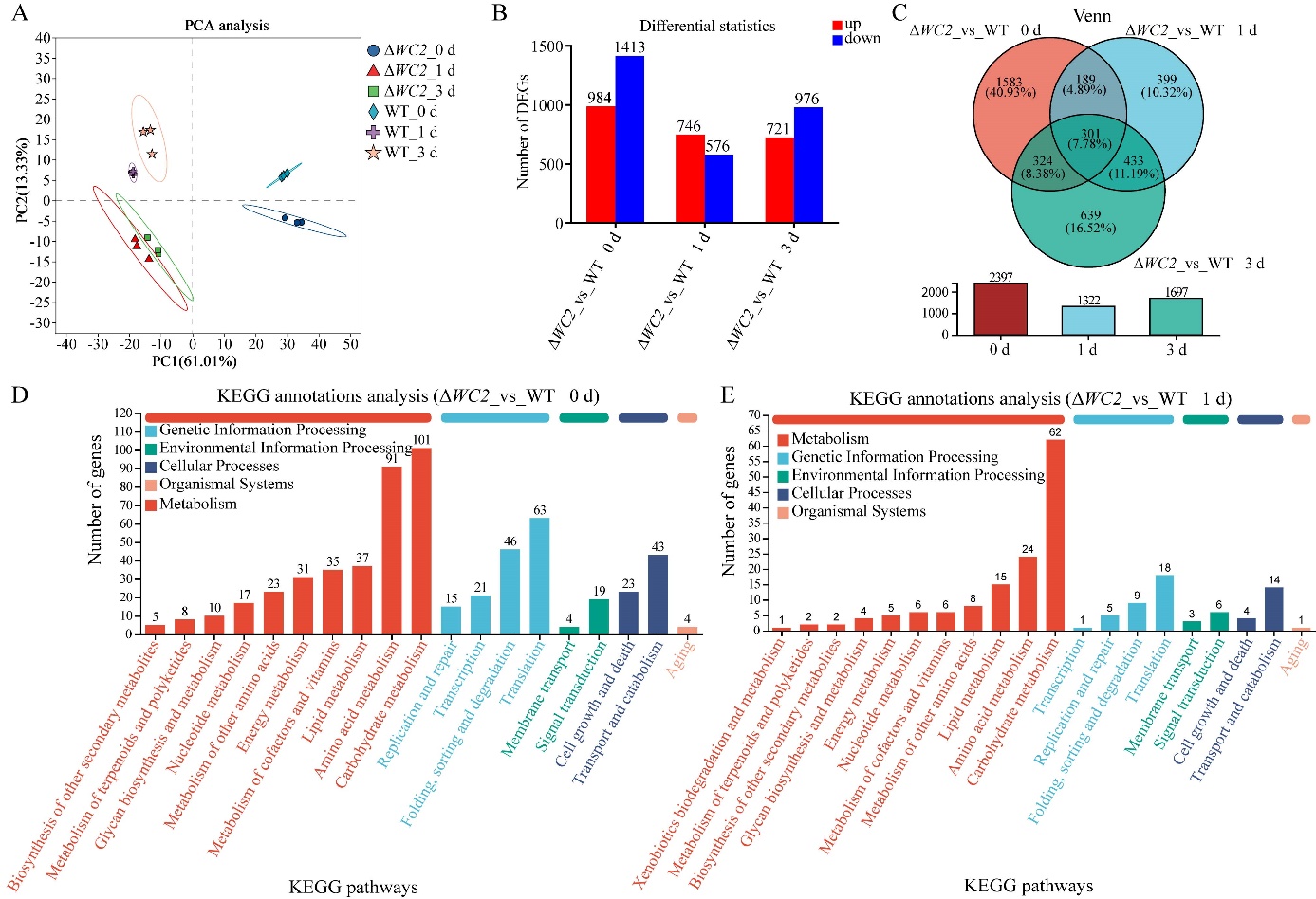


Figure S22 RNA-seq analysis of WT and Δ*WC2* mutant strains in chlamydospore formation.

(A) Principal Component Analysis (PCA) plots for WT and Δ*WC2* mutant strain at 0, 1, and 3 days. (B) Differentially expressed genes (DEGs) analysis in Δ*WC2* mutant strain at 0, 1, and 3 days. (C) Venn diagram showing the overlap of DEGs in the Δ*WC2* mutant at 0, 1, and 3 days. (D–E) KEGG annotation analysis of DEGs in the Δ*WC2* mutant at day 0 (D) and day 1 (E).

## Figure S23


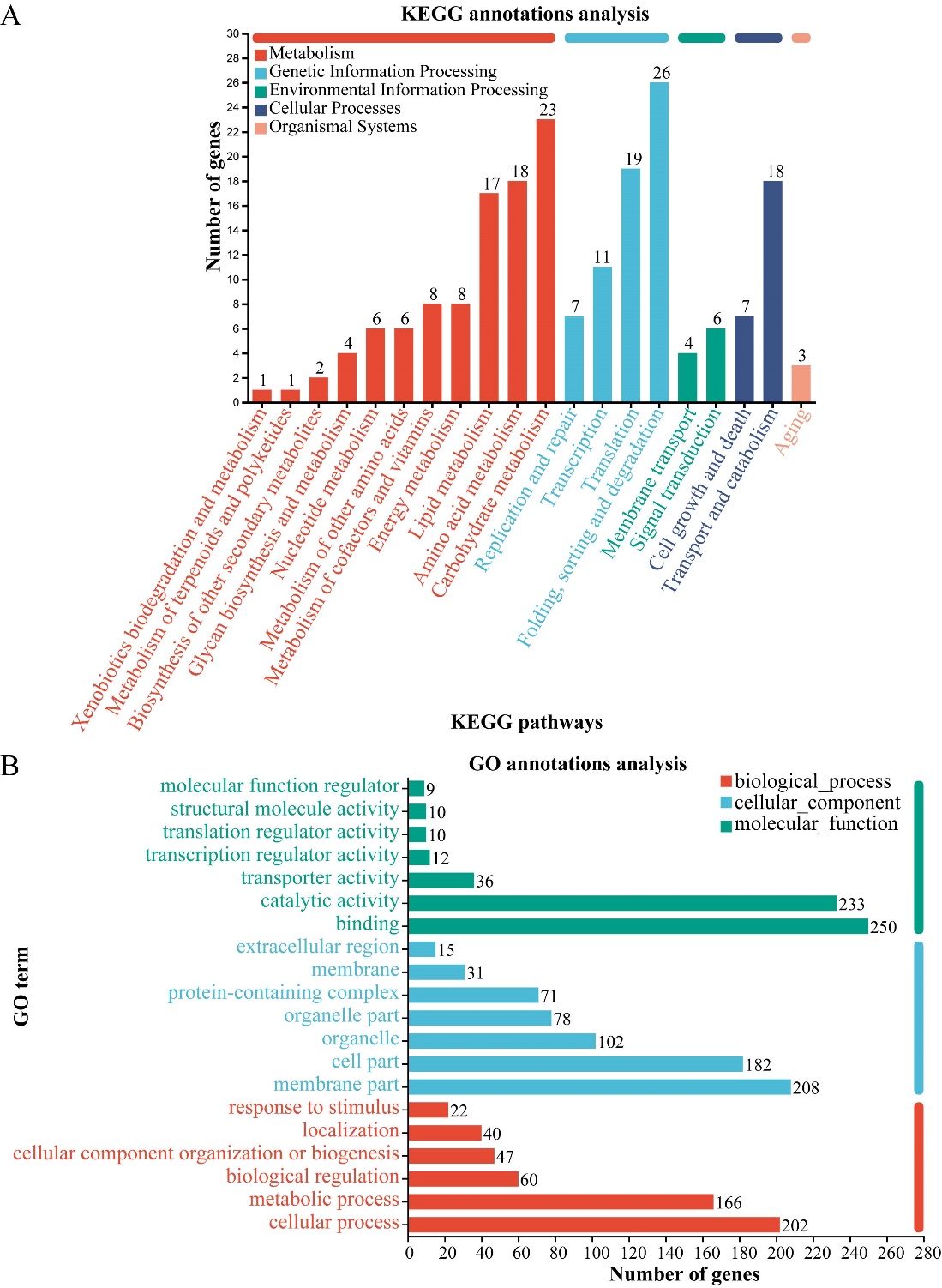


Figure S23 KEGG and GO annotation analysis of WC2 target genes.

(A) Top 20 KEGG annotation pathways of WC2 target genes. (B) Top 20 GO annotation terms of WC2 target genes.

## Figure S24


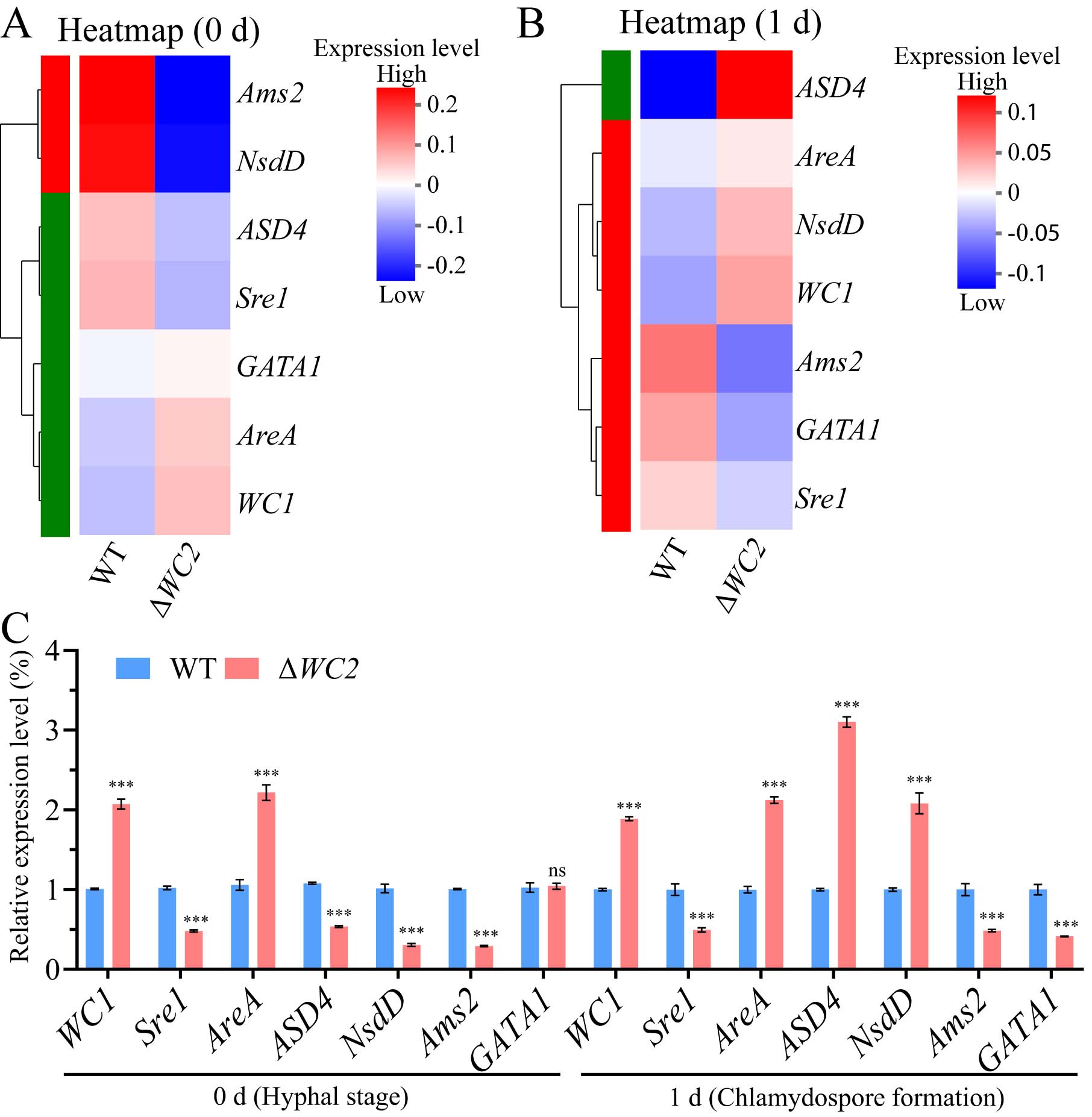


Figure S24 Analysis of expression level of the other seven GATA-type transcription factors in the Δ*WC2* mutant strain.

(A) and (B) Expression levels of the other seven GATA-type transcription factors in the Δ*WC2* mutant strain during the hyphal stage (A) and chlamydospore formation (B) were analyzed. (C) RT-qPCR analysis of the expression levels of other GATA-type transcription factors in the Δ*WC2* mutant strain (Student's *t*-test; ****p* < 0.001).

## Figure S25


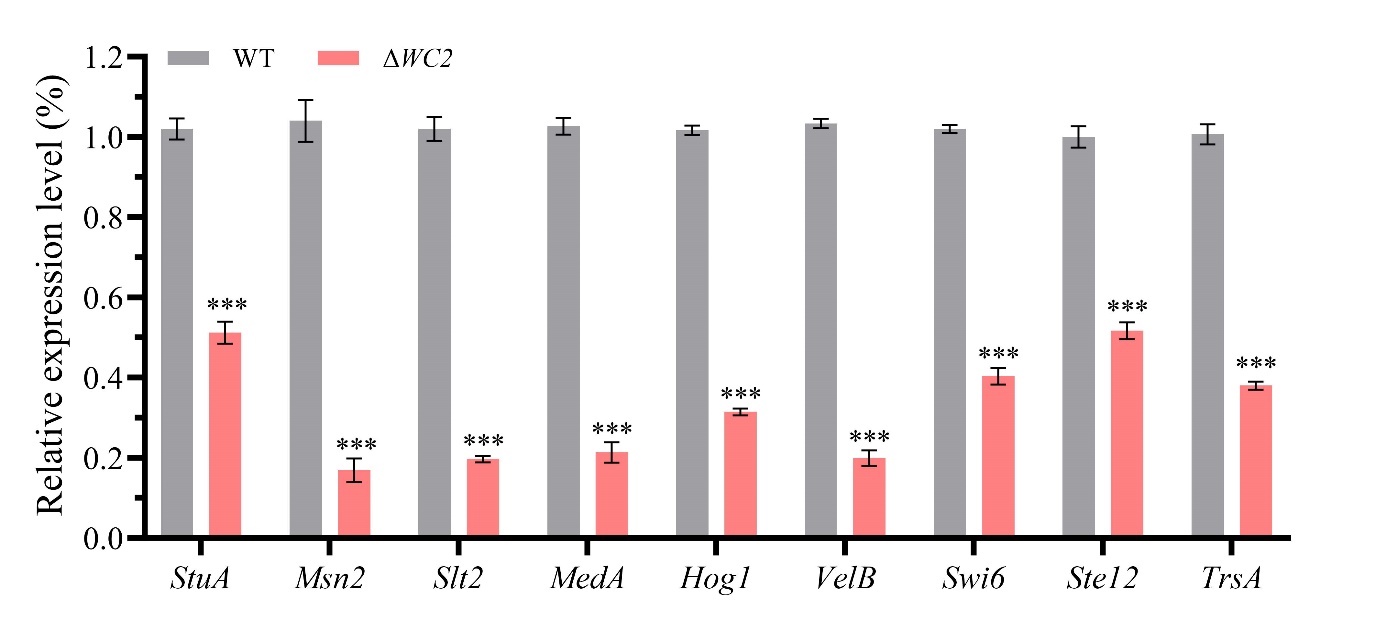


Figure S25 RT-qPCR analysis of the expression levels of trap formation-related genes and virulence factors in the Δ*WC2* mutants.

Student's *t*-test; ****p* < 0.001.

## Figure S26


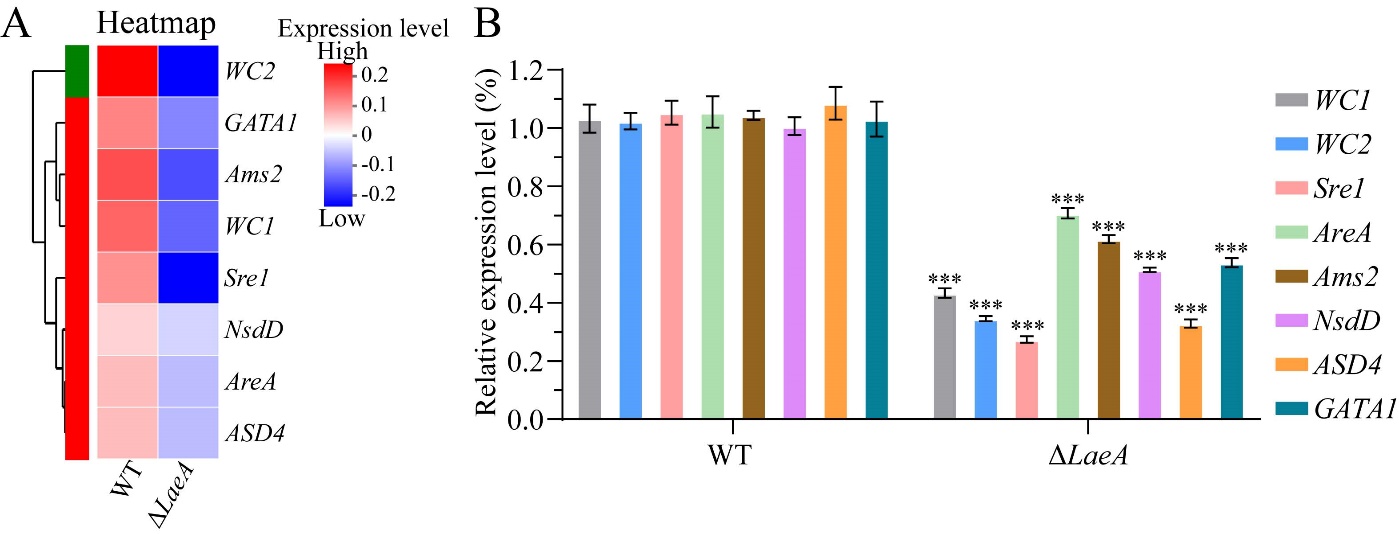


Figure S26 Analysis of the effect of the global regulator LaeA on the expression of eight GATA-type transcription factors.

Expression levels of eight GATA-type transcription factors in the Δ*laeA* mutant strain, as determined by RNA-seq (A) and RT-qPCR (B) analyses (Student's *t*-test; **p* < 0.05, ***p* < 0.01, ****p* < 0.001).

## Figure S27


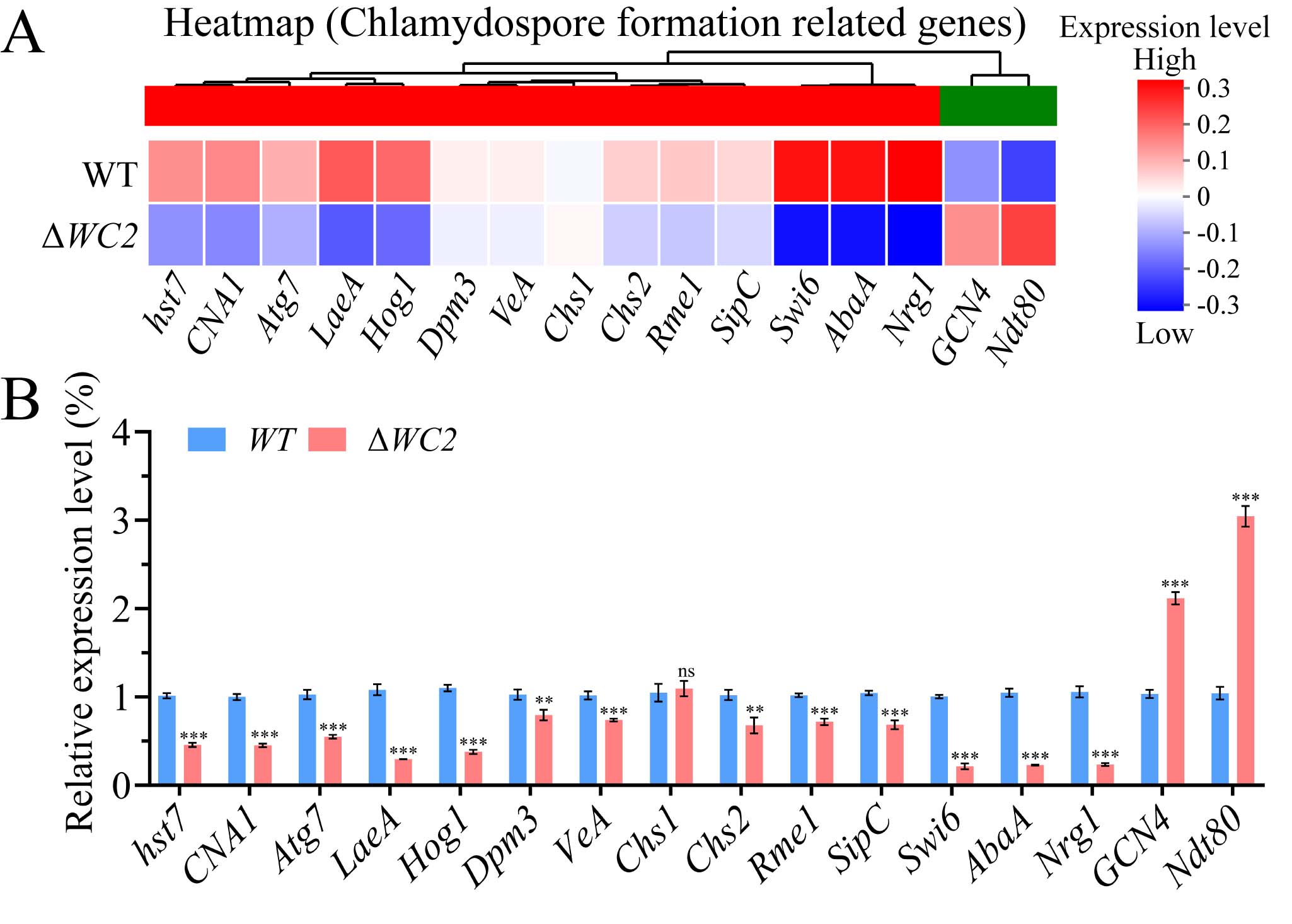


Figure S27 Effect of WC2 on the expression of chlamydospore formation-related genes.

(A) RNA-seq analysis of the expression levels of chlamydospore formation-related genes in the Δ*WC2* mutant strain. (B) RT-qPCR analysis of the expression levels of chlamydospore formation-related genes in the Δ*WC2* mutant strain (Student's *t*-test; **p* < 0.05, ***p* < 0.01, ****p* < 0.001).

Supplementary Table

## Table S1 *A. flagrans* strains and plasmids used in this study.

| Strains/Plasmids | Genotype | Description | Catalogue number | Reference |
| --- | --- | --- | --- | --- |
| *A. flagrans* | WT | WT | YMF1.01471 | This study |
| Δ*WC1* | Δ*WC1*; *hph* | Gene deletion of *WC1* | YMF1.07964 | This study |
| Δ*WC2* | Δ*WC2*; *hph* | Gene deletion of *WC2* | YMF1.07965 | This study |
| Δ*Sre1* | Δ*Sre1*; *hph* | Gene deletion of *Sre1* | YMF1.07966 | This study |
| Δ*AreA* | Δ*AreA*; *hph* | Gene deletion of *AreA* | YMF1.07967 | This study |
| Δ*Ams2* | Δ*Ams2*; *hph* | Gene deletion of *Ams2* | YMF1.07968 | This study |
| Δ*ASD4* | Δ*ASD4*; *hph* | Gene deletion of *ASD4* | YMF1.07969 | This study |
| Δ*NsdD* | Δ*NsdD*; *hph* | Gene deletion of *NsdD* | YMF1.07970 | This study |
| Δ*GATA1* | Δ*GATA1*; *hph* | Gene deletion of *GATA1* | YMF1.07971 | This study |
| *WC1-EGFP* | *WC1(p)*::*WC1*::*GFP*::*WC1(t);* *hph* | GFP-tagging of WC1 | YMF1.07972 | This study |
| *WC2-EGFP* | *WC2(p)*::*WC2*::*GFP*::*WC2(t);* *hph* | GFP-tagging of WC2 | YMF1.08004 | This study |
| *OEWC2* | *H2B(p)*:: *WC2*::*H2B(t)*;*G418* | Overexpression of *WC2* | YMF1.08005 | This study |
| *N^-WC1^* | *WC1(p)*::*WC1*::*GFP^N^*::*WC1(t);* *hph* | BiFC | YMF1.08006 | This study |
| *C^-WC2^* | *WC1(p)*::*WC2*::*GFP^C^*::*WC2(t);G418* | BiFC | YMF1.08007 | This study |
| *N^-WC1^*::*C^-WC2^* | *WC1(p)*::*WC1*::*GFP^N^*::*WC1(t);* *hph*  *WC1(p)*::*WC2*::*GFP^C^*::*WC2(t);G418* | BiFC | YMF1.08008 | This study |
| Δ*WC1*::*Sre1* | Δ*WC1; hph*  *H2B(p)*:: *Sre1*::*H2B(t)*;*G418* | *Sre1* expression in Δ*WC1* mutants | YMF1.08009 | This study |
| Δ*WC2*::*Sre1* | Δ*WC2; hph*  *H2B(p)*:: *Sre1*::*H2B(t)*;*G418* | *Sre1* expression in Δ*WC2* mutants | YMF1.08010 | This study |
| Δ*WC1^C^* | Δ*WC1*; *hph*  *WC1(p)*:: *WC1*::*WC1(t)*;*G418* | Complementation of *WC1* | YMF1.08011 | This study |
| Δ*WC2^C^* | Δ*WC2*; *hph*  *WC2(p)*:: *WC2*::*WC2(t)*;*G418* | Complementation of *WC2* | YMF1.08012 | This study |
| Δ*Sre1^C^* | Δ*Sre1*; *hph*  *Sre1(p)*::*Sre1*::*Sre1(t)*;*G418* | Complementation of *Sre1* | YMF1.08013 | This study |
| Δ*AreA^C^* | Δ*AreA*; *hph*  *AreA(p)*::*AreA*::*AreA(t)*;*G418* | Complementation of *AreA* | YMF1.08014 | This study |
| Δ*Ams2^C^* | Δ*Ams2*; *hph*  *Ams2(p)*::*Ams2*::*Ams2(t)*;*G418* | Complementation of *Ams2* | YMF1.07587 | This study |
| Δ*ASD4^C^* | Δ*ASD4*; *hph*  *ASD4(p)*::*ASD4*::*ASD4(t)*;*G418* | Complementation of *ASD4* | YMF1.07588 | This study |
| Δ*NsdD^C^* | Δ*NsdD*; *hph*  *NsdD(p)*::*NsdD*::*NsdD(t)*;*G418* | Complementation of *NsdD* | YMF1.07589 | This study |
| Δ*GATA1^C^* | Δ*GATA1*; *hph*  *GATA1(p)*::*GATA1*::*GATA1(t)*;*G418* | Complementation of *GATA1* | YMF1.07590 | This study |
| Δ*laeA* | Δ*laeA; hph* | Gene deletion of *LaeA* | YMF1.07558 | Zhang et al. 2023 |

The catalogue number refers to the strain designation in the Microbial Library of the Germplasm Bank of Wild Species from Southwest China.

## Table S2 Primers used in this study.

| Primers | Primer sequences (5'-3') | | Description |
| --- | --- | --- | --- |
| *Gpd-*for | CGAGAAGCCCGCCAAGT | | qPCR |
| *Gpd*-rev | AAAGGTGTCGGTCAAAGCAAT | |  |
| RT-*Sre1-*for | TGTGATAAAGCGGCGGAAAC | |  |
| RT-*Sre1*-rev | TTGATGGCTGCGGTGAGG | |  |
| RT-*GATA1*-for | ACGTATCAGCCTATCCAACCG | |  |
| RT-*GATA -*rev | TGGTGGCGATACTAAACTCAAATAC | |  |
| RT-*WC2*-for | CATTACGCTCCGACTCCCTT | |  |
| RT-*WC2*-rev | CTATCCAGTGTCCCACAATCC | |  |
| RT-*WC1*-for | TCAAACAACAATGTGACGACCTG | |  |
| RT-*WC1*-rev | CTGTCCCAAGAAATCGGAATAG | |  |
| RT-*AreA*-for | TGGCGTTGTTCGCCCTTTATC | |  |
| RT-*AreA*-rev | TGCTCCAGAGCCGCTATTAGTC | |  |
| RT-*Ams2*-for | CTTTCGTTACCGACGCTCAA | |  |
| RT-*Ams2-*rev | CCCATCGCATTCGTCCATACA | |  |
| RT-*ASD4*-for | ATCAGTCATCTGAAGACGAGCACA | |  |
| RT-*ASD4*-rev | GGGTTGGCACTCGGAGAACTG | |  |
| RT-*NsdD*-for | TATAGTTTCAACGGGTGGTGC | |  |
| RT-*NsdD*-rev | CGCGTAAGGCTGACGATGGAG | |  |
| AD-*WC2*-for | gtaccagattacgctATGGATCGGGTGAACGGC | | Y2H |
| AD-*WC2*-rev | atgcccacccgggtgTCAAATCATTCCATGGTTTTGC | |  |
| BD-*WC1*-for | tcagaggaggacctgATGATGGAACATCACGATTTGGA | | Y2H |
| BD-*WC1*-rev | tcgacggatccccggTCAACGATCAATGAAGGTATCATCTC | |  |
| AD-*ASD4*-for | gtaccagattacgctATGATCGAACTGTCCCCGG | | Y2H |
| AD-*ASD4*-rev | atgcccacccgggtgTTATACAGCCGACTGGGGAGTC | |  |
| AD-*Sre1*-for | gtaccagattacgctATGGCGAACCCTGAACCG | | Y2H |
| AD-*Sre1*-rev | atgcccacccgggtgCTAATTACCAGGGTTCGGTTCG | |  |
| BD-*AreA*-for | tcagaggaggacctgATGGACTTATCAAGCTCGACATCC | | Y2H |
| BD-*AreA*-rev | tcgacggatccccggCTACAGGGACATGGTGAGCCA | |  |
| P-*Sre1*-for | actatagggcgaattCCAGTTCCTATCTTGCGATAATCG | | Y1H |
| P-*Sre1*-rev | cgaacgcgtgagctcTTTTATCTAATGTTATCCCAGCATAGG | |  |
| Ko*Sre1*-up*-*for | ttcggatcttccagaTGCCAGGTAGTACTTGCTCGACC | Amplify the 5' homologous fragment of *Sre1* | |
| Ko*Sre1*-up-rev | tatcatcttctgGGCATGCGGTTCAGGGTT |  |  |
| Ko*Sre1*-hyg*-*for | cgcatgccCAGAAGATGATATTGAAGGAGCATTT | Amplify the *hph* cassette | |
| Ko*Sre1*-hyg-rev | cccgaaggcatAAAGAAGGATTACCTCTAAACAAGTGTACC |  |  |
| Ko*Sre1-*down-for | tccttctttATGCCTTCGGGAGGGATTT | Amplify the 3' homologous fragment of *SRE1* | |
| Ko*Sre1*-down*-*rev | caactgccgttcgacATTGAAGGGGCGTAGCTTAAGC |  |  |
| Ko*GATA1*-up*-*for | ttcggatcttccagaAGTTTAGAAGTCCCAAGAGGAAACA | Amplify the 5' homologous fragment of *GATA1* | |
| Ko*GATA1*-up-rev | tcatcttctgCTCACTTCCGAGATCCAGGGA |  |  |
| Ko*GATA1*-hyg*-*for | cggaagtgagCAGAAGATGATATTGAAGGAGCATTT | Amplify the *hph* cassette | |
| Ko*GATA1*-hyg-rev | gatctcccttgtAAAGAAGGATTACCTCTAAACAAGTGTACC |  |  |
| Ko*GATA1*-down-for | ccttctttACAAGGGAGATCAAGGTGTTAATTT | Amplify the 3' homologous fragment of *GATA1* | |
| Ko*GATA1*-down*-*rev | caactgccgttcgacGTTCCCAAAGCCATAACTAGTTACC |  |  |
| Ko*WC2*-up*-*for | ttcggatcttccagaAAGGCCTTGCACACGATGTC | Amplify the 5' homologous fragment of *WC2* | |
| Ko*WC2*-up-rev | CcttcaatatcatcttctgCTTTATACAATATTATATTGTATATTATATTATGTAAATATCTT |  |  |
| Ko*WC2*-hyg*-*for | gCAGAAGATGATATTGAAGGAGCATTT | Amplify the *hph* cassette | |
| Ko*WC2*-hyg-rev | caactgccgttcgacgatatcAAAGAAGGATTACCTCTAAACAAGTGTACC |  |  |
| Ko*WC2*-down-for | aggtaatccttctttGTAAACGCTGACGGATATCTATATCTAA | Amplify the 3' homologous fragment of *WC2* | |
| Ko*WC2*-down*-*rev | caactgccgttcgacTCAAATCATTCCATGGTTTTGC |  |  |
| Ko*AreA*-up*-*for | ttcggatcttccagaTCTAAAAATCTAAAAATCCTGCCATG | Amplify the 5' homologous fragment of *AreA* | |
| Ko*AreA*-up-rev | tcatcttctgCGCATTAGGGGAGGCTGTT |  |  |
| Ko*AreA*-hyg*-*for | ccctaatgcgCAGAAGATGATATTGAAGGAGCATTT | Amplify the *hph* cassette | |
| Ko*AreA*-hyg-rev | gagggtattctttgaggtttAAAGAAGGATTACCTCTAAACAAGTGTACC |  |  |
| Ko*AreA*-down-for | AAACCTCAAAGAATACCCTCAGACG | Amplify the 3' homologous fragment of *AreA* | |
| Ko*AreA*-down*-*rev | caactgccgttcgacCACAGCATTTCAAACATAAAAGACTACA |  |  |
| Ko*Ams2*-up*-*for | ttcggatcttccagaCGTTCTGACGGCGGTCTG | Amplify the 5' homologous fragment of *Ams2* | |
| Ko*Ams2*-up-rev | aatatcatcttctgGGCCCCCTGTGAGGCCAG |  |  |
| Ko*Ams2*-hyg*-*for | ggggccCAGAAGATGATATTGAAGGAGCATTT | Amplify the *hph* cassette | |
| Ko*Ams2*-hyg-rev | gggggtgtcAAAGAAGGATTACCTCTAAACAAGTGTACC |  |  |
| Ko*Ams2A*-down-for | atccttctttGACACCCCCGCGCCAGCC | Amplify the 3' homologous fragment of *Ams2* | |
| Ko*Ams2*-down*-*rev | caactgccgttcgacGTTGTGATGCCATAGTCCGCA |  |  |
| Ko*ASD4*-up*-*for | ttcggatcttccagaGGATGCCCCCATGCTCAG | Amplify the 5' homologous fragment of *ASD4* | |
| Ko*ASD4*-up-rev | ctgGTAATTAAAGGAGGGAGAAATCGG |  |  |
| Ko*ASD4*-hyg*-*for | ctccctcctttaattacCAGAAGATGATATTGAAGGAGCATTT | Amplify the *hph* cassette | |
| Ko*ASD4*-hyg-rev | gcgaagtcaagcAAAGAAGGATTACCTCTAAACAAGTGTACC |  |  |
| Ko*ASD4*-down-for | ccttctttGCTTGACTTCGCTTGGGCA | Amplify the 3' homologous fragment of *ASD4* | |
| Ko*ASD4*-down*-*rev | caactgccgttcgacAAAGCTTGTGATAGTACATACAATGCA |  |  |
| Ko*NsdD*-up*-*for | ttcggatcttccagaGGCCCCTCTTGGTTCCCT | Amplify the 5' homologous fragment of *NsdD* | |
| Ko*NsdD*-up-rev | tcttctgATCTACTACCGCCTCGGCCT |  |  |
| Ko*NsdD*-hyg*-*for | ggcggtagtagatCAGAAGATGATATTGAAGGAGCATTT | Amplify the *hph* cassette | |
| Ko*NsdD*-hyg-rev | ttggctccctgAAAGAAGGATTACCTCTAAACAAGTGTACC |  |  |
| Ko*NsdD*-down-for | tccttctttCAGGGAGCCAACCGTTTCC | Amplify the 3' homologous fragment of *NsdD* | |
| Ko*NsdD*-down*-*rev | caactgccgttcgacCTTGTGTCCAAGGTAAATCTGGTG |  |  |
| Ko*WC1*-up*-*for | ttcggatcttccagaAGGTTGTGCCGCCTTGTATC | Amplify the 5' homologous fragment of *WC1* | |
| Ko*WC1*-up-rev | tctgGGTTAGTATTTCCCGGTCGAAA |  |  |
| Ko*WC1*-hyg*-*for | ccgggaaatactaaccCAGAAGATGATATTGAAGGAGCATTT | Amplify the *hph* cassette | |
| Ko*WC1*-hyg-rev | ctctAAAGAAGGATTACCTCTAAACAAGTGTACC |  |  |
| Ko*WC1*-down-for | gaggtaatccttctttAGAGATATTATTTCACTTCTAGTTTATCAGGG | Amplify the 3' homologous fragment of *WC1* | |
| Ko*WC1*-down*-*rev | caactgccgttcgacATATGTCGTATTAGTTATCTAGTGCCTGAA |  |  |
| *WC2-GFP* | aaaacaaatttcaaaATGGATCGGGTGAACGGC | GFP fluorescence labeling of WC2 | |
|  | gcccttgctcaccatAATCATTCCATGGTTTTGC |  |  |
| *WC1-GFP* | aaaacaaatttcaaaATGATGGAACATCACGATTTGGA | GFP fluorescence labeling of WC1 | |
|  | gcccttgctcaccatTCAACGATCAATGAAGGTATCATCTC |  |  |
| *WC2-NGFP* | aggtggatctATGGATCGGGTGAACGGC | Fusion of the N-terminal fragment of GFP to WC2 | |
|  | caactgccgttcgacAATCATTCCATGGTTTTGC |  |  |
| *WC1-CGFP* | ttcggatcttccagaATGATGGAACATCACGATTTGGA | Fusion of the C-terminal fragment of GFP to WC1 | |
|  | gtaccTCAACGATCAATGAAGGTATCATCTC |  |  |

## Table S3 The predicted and tallied physiochemical properties of eight GATA proteins in *A.* *flagrans*.

| Gene ID | GeneBank ID | Name | GATA-type zinc finger motif | ORF length (bp) | Exon/Intron Number | Amino acids (aa) | Molecular weight (kDa) | Subcellular localization |
| --- | --- | --- | --- | --- | --- | --- | --- | --- |
| DFL_005854/EVM04G004560 | XP_067489630.1 | Sre1 | CX_2_CX_17_CX_2_C | 2103 | 3/2 | 620 | 66.455 | Nucleus |
| DFL_000775/EVM05G007890 | XP_067495326.1 | ASD4 | CX_2_CX_17_CX_2_C | 2746 | 5/4 | 368 | 40.207 | Nucleus |
| DFL_004967/EVM02G004450 | XP_067492249.1 | AreA | CX_2_CX_17_CX_2_C | 2246 | 2/1 | 723 | 76.602 | Nucleus |
| DFL_006252/EVM04G008530 | XP_067490047.1 | WC1 | CX_2_CX_18_CX_2_C | 3101 | 6/5 | 916 | 101.014 | Nucleus |
| DFL_005233/EVM02G001780 | XP_067492527.1 | WC2 | CX_2_CX_18_CX_2_C | 2223 | 6/5 | 590 | 64.579 | Nucleus |
| DFL_009527/EVM00G015330 | XP_067487219.1 | NsdD | CX_2_CX_18_CX_2_C | 2076 | 6/5 | 511 | 54.944 | Nucleus |
| DFL_002521/EVM01G010980 | XP_067493876.1 | GATA1 | CX_2_CX_20_CX_2_C | 2823 | 3/2 | 750 | 82.651 | Nucleus |
| DFL_008588/EVM00G005850 | XP_067486238.1 | Ams2 | CX_2_CX_18_EX_2_S | 4119 | 3/2 | 1319 | 140.635 | Nucleus |

## Table S4 Per‑trap killing efficiency at 12 h and 24 h post‑nematode addition.

| Strain | 12 h | | | 24 h | | |
| --- | --- | --- | --- | --- | --- | --- |
|  | Mortality per trap | Fold change vs. WT | Significance | Mortality per trap | Fold change vs. WT | Significance |
| WT | 0.146 ± 0.018 | 1.00 | - | 0.306 ± 0.036 | 1.00 | - |
| Δ*NsdD* | 0.352 ± 0.025 | 2.41 | *** | 0.365 ± 0.024 | 1.19 | ns |
| Δ*Ams2* | 0.265 ± 0.019 | 1.82 | *** | 0.278 ± 0.026 | 0.91 | ns |
| Δ*ASD4* | 0.205 ± 0.015 | 1.40 | ** | 0.303 ± 0.021 | 0.99 | ns |
| OE*WC2* | 0.196 ± 0.020 | 1.34 | * | 0.237 ± 0.018 | 0.77 | * |
| Δ*WC2* | 0.139 ± 0.040 | 0.95 | ns | 0.320 ± 0.070 | 1.04 | ns |
| Δ*AreA* | 0.139 ± 0.024 | 0.95 | ns | 0.512 ± 0.078 | 1.67 | *** |
| Δ*WC1* | 0.133 ± 0.011 | 0.91 | ns | 0.365 ± 0.047 | 1.19 | * |
| Δ*Sre1* | 0.132 ± 0.009 | 0.90 | ns | 0.264 ± 0.062 | 0.86 | * |
| Δ*GATA1* | 0.036 ± 0.012 | 0.25 | *** | 0.070 ± 0.016 | 0.23 | ** |

Data are presented as mean ± SD from three independent replicates. Higher values indicate higher per‑trap killing efficiency. Significance was determined by Student’s *t*‑test compared to WT at the same time point. ns, not significant; **P* < 0.05; ***P* < 0.01; ****P* < 0.001.

## Table S5 Protein details in the protein-protein interaction network predicted by STRING.

| Gene ID | GeneBank ID | Descirption |
| --- | --- | --- |
| DFL_002580 | XP_067493937.1 | NmrA domain-containing protein. |
| DFL_007411 | XP_067488551.1 | BZIP domain-containing protein. |
| DFL_001827 | XP_067493149.1 | MADS-box domain-containing protein. |
| DFL_002546 | XP_067493903.1 | Thioredoxin domain-containing protein. |
| DFL_000541 | XP_067495082.1 | ubiquitinyl hydrolase 1. |
| DFL_007032 | XP_067488155.1 | Importin subunit alpha; Belongs to the importin alpha family. |
| DFL_007755 | XP_067488909.1 | Fork-head domain-containing protein. |
| DFL_002833 | XP_067494200.1 | Beta-galactosidase. |
| DFL_001041 | XP_067495604.1 | Beta-galactosidase. |
| DFL_006804 | XP_067487922.1 | Tryptophan synthase. |
| DFL_001815 | XP_067493134.1 | Actin-related protein 8. |
| DFL_007936 | XP_067489095.1 | Uncharacterized protein. |
| DFL_007175 | XP_067488304.1 | Endoplasmic reticulum lectin. |
| DFL_001653 | XP_067492961.1 | UBX domain-containing protein. |
| DFL_005954 | XP_067489734.1 | PH-response regulator protein palH/RIM21. |
| DFL_000799 | XP_067495351.1 | Uncharacterized protein. |
| DFL_009006 | XP_067486674.1 | NmrA domain-containing protein. |
| DFL_002596 | XP_067493953.1 | Histone H2A. |
| DFL_001827 | XP_067493149.1 | MADS-box domain-containing protein. |
| DFL_002498 | XP_067493853.1 | TATA-box-binding protein. |
| DFL_009363 | XP_067487043.1 | Homeobox protein YOX1. |
| DFL_009060 | XP_067486730.1 | INO80 chromatin remodeling complex IES1. |

## Table S6 Downstream target genes of WC2 identified through integrated RNA-seq and DAP-seq analyses in chlamydospore formation.

| No. | Gene ID | Name | Protein Description | GO Description |
| --- | --- | --- | --- | --- |
| 1 | EVM04G004560 | Sre1 | GATA-type transcription factor SRE1 | transcription factor activity, sequence-specific DNA binding |
| 2 | EVM00G017500 | - | UV-damage endonuclease | mitochondrion; nucleus; endonuclease activity; hydrolase activity |
| 3 | EVM01G012620 | - | Uncharacterized protein | cellular_component |
| 4 | EVM03G014360 | CsgA | Transcriptional regulatory protein CsgA | sequence-specific DNA binding |
| 5 | EVM00G013410 | - | ABM domain-containing protein | cellular_component |
| 6 | EVM00G013260 | - | Cytochrome P450 | heme binding; iron ion binding; monooxygenase activity |
| 7 | EVM00G010250 | - | Conidiation-specific expression protein | - |
| 8 | EVM00G006990 | - | F-box domain-containing protein | cellular_component |
| 9 | EVM00G004400 | Sed3 | tripeptidyl-peptidase II (EC 3.4.14.10) | metal ion binding; serine-type endopeptidase activity; tripeptidyl-peptidase activity |
| 10 | EVM00G000310 | - | Glutamate decarboxylase 1 | glutamate metabolic process |
| 11 | EVM00G001020 | - | F-box domain-containing protein | - |
| 12 | EVM00G000760 | - | F-box domain-containing protein | - |
| 13 | EVM03G014360 | Pro1 | Zn(2)-C6 fungal-type domain-containing protein | nucleus; DNA-binding transcription factor activity; zinc ion binding |
| 14 | EVM03G014250 | - | F-box domain-containing protein | - |
| 15 | EVM03G011180 | - | Probable acetate kinase (EC 2.7.2.1) (Acetokinase) | acetate kinase activity;magnesium ion binding |
| 16 | EVM03G011110 | - | ubiquitinyl hydrolase 1 (EC 3.4.19.12) | cysteine-type peptidase activity |
| 17 | EVM03G009780 | - | Uncharacterized protein | cellular_component |
| 18 | EVM03G004970 | ALKBH2 | Fe2OG dioxygenase domain-containing protein | ferrous iron binding |
| 19 | EVM03G002710 | - | 3CxxC-type domain-containing protein | - |
| 20 | EVM03G001960 | - | Uncharacterized protein | cell wall; ubiquitin ligase complex; ubiquitin-dependent protein catabolic process |
| 21 | EVM03G001540 | - | Shugoshin C-terminal domain-containing protein | - |
| 22 | EVM03G001450 | - | Glycine zipper domain-containing protein | integral component of membrane |
| 23 | EVM04G011080 | - | Major facilitator superfamily (MFS) profile domain-containing protein | carbohydrate:proton symporter activity |
| 24 | EVM04G002710 | - | Uncharacterized protein | integral component of membrane |
| 25 | EVM04G000870 | - | AAA+ ATPase domain-containing protein | nuclear RNA-directed RNA polymerase complex; helicase activity |
| 26 | EVM04G000500 | - | Sulfite efflux pump SSU1 | plasma membrane; sulfite transmembrane transporter activity |
| 27 | EVM02G003000 | - | F-box domain-containing protein | - |
| 28 | EVM02G007770 | - | Hypervirulence associated protein TUDOR domain-containing protein | hydrolase activity |
| 29 | EVM02G008380 | - | Amine oxidase | alpha-(1->6)-fucosyltransferase activity; N-glycan fucosylation; protein N-linked glycosylation |
| 30 | EVM02G010390 | - | Hydrophobin | - |
| 31 | EVM02G019430 | - | argininosuccinate lyase (EC 4.3.2.1) (Arginosuccinase) | argininosuccinate lyase activity |
| 32 | EVM02G019440 | - | 4-dimethylallyltryptophan N-methyltransferase (EC 2.1.1.261) | methyltransferase activity; alkaloid metabolic process |
| 33 | EVM02G021280 | - | Uncharacterized protein | - |
| 34 | EVM02G021350 | - | Uncharacterized protein | - |
| 35 | EVM01G016940 | - | C2H2-type domain-containing protein | - |
| 36 | EVM01G016210 | - | Uncharacterized protein | - |
| 37 | EVM01G016190 | - | Uncharacterized protein | - |
| 38 | EVM01G016180 | - | Copper-fist domain-containing protein | - |
| 39 | EVM01G015180 | - | Uncharacterized protein | cellular_component |
| 40 | EVM01G008360 | - | PIPK domain-containing protein | 1-phosphatidylinositol-4-phosphate 5-kinase activity; ATP binding |
| 41 | EVM01G004740 | - | S-formylglutathione hydrolase (EC 3.1.2.12) | carboxylic ester hydrolase activity |
| 42 | EVM01G001210 | - | carnosine N-methyltransferase (EC 2.1.1.22) | S-adenosylmethionine-dependent methyltransferase activity |
| 43 | EVM01G000500 | - | Beta-xylanase (EC 3.2.1.8) | endo-1,4-beta-xylanase activity; xylan catabolic process |
| 44 | EVM05G012610 | - | Uncharacterized protein | integral component of membrane |
| 45 | EVM05G004810 | - | ubiquitinyl hydrolase 1 (EC 3.4.19.12) | cysteine-type peptidase activity |
| 46 | EVM05G004600 | - | Rhodopsin domain-containing protein | integral component of membrane |
| 47 | EVM05G004590 | - | Uncharacterized protein | - |
| 48 | EVM05G003730 | - | Uncharacterized protein | - |

“-” indicated that no functional description is available for this protein.

## Table S7 qPCR analysis to validate the series of potential genes regulated by Sre1.

| Gene ID | Name | Description | Function |
| --- | --- | --- | --- |
| EVM02G009470 | *FDFT1* | Squalene synthase | Ergosterol biosynthesis |
| EVM05G002020 | *ERG4* | Delta(24(24(1)))-sterol reductase | Ergosterol biosynthesis |
| EVM03G012670 | *CYP51* | Cytochrome P450 | Ergosterol biosynthesis |
| EVM05G004640 | *CDC42* | Serine Threonine protein kinase | MAPK signaling pathway |
| EVM05G008210 | *Hog1* | Mitogen-activated protein kinase | MAPK signaling pathway |
| EVM05G009000 | *Swi6* | Transcription factor | MAPK signaling pathway |
| EVM02G003540 | *UGP2* | UTP-glucose-1-phosphate uridylyltransferase | UDP‑glucose synthesis |
| EVM00G006520 | *FKS1* | 1,3-beta-glucan synthase | β‑glucan synthesis |
| EVM05G010260 | *Chs2* | Chitin synthase 2 | Chitin synthesis |
| EVM03G010710 | *CDA2* | Chitin deacetylase | Chitosan biosynthesis |

Supplementary Materials and Methods

## S1. Strains and culture conditions

The wild-type (WT) strain *A. flagrans* CBS 565.50 was grown on potato dextrose agar (PDA) medium at 28°C. Mutants and transgenic strains were maintained on PDA supplemented with the appropriate antibiotics: knockout mutants, *WC1-EGFP*, *WC2-EGFP*, and *N^-WC1^* strains with 100 μg/mL hygromycin B (Sigma-Aldrich, 400051); *OEWC2* and *C^-WC2^* strains with 50 μg/mL G418 (Sigma-Aldrich, G8168); and Δ*WC1*::*Sre1*, Δ*WC2*::*Sre1*, and *N^-WC1^*::*C^-WC2^* strains with both 50 μg/mL G418 and 100 μg/mL hygromycin B. A comprehensive list of all strains used can be found in Table S1. All strains were stored in the Microbial Library of the Germplasm Bank of Wild Species from Southwest China.

## S2. Gene structure and conserved protein domain analysis

The gene structures of the eight GATA-type transcription factors were analyzed using the Gene Structure Display Server (<http://gsds.cbi.pku.edu.cn/>). Conserved domains were predicted with the NCBI CD-Search tool (<https://www.ncbi.nlm.nih.gov/Structure/cdd/wrpsb.cgi>) and visualized using TBtools software. For phylogenetic analysis, sequences were aligned in MEGA-X, and a neighbor-joining tree was constructed with 1000 bootstrap replicates.

## S3. Gene deletion and complementation

To construct deletion mutants for each of the eight GATA-type transcription factor genes, upstream and downstream homologous arms (~1 kb each) were designed and amplified from genomic DNA using primers generated with CE Design software (https://tool.vazyme.com:18002/cetool/multifragment.html). The hygromycin resistance gene (*hph*) was amplified from plasmid pCSN44. For each gene, the upstream arm, *hph*, and downstream arm were then assembled in order into the pUC19 vector with the ClonExpress Ultra One Step Cloning Kit (Vazyme, C115-02), generating a gene-specific knockout plasmid (Zhang et al. 2023). Finally, the linear gene-targeting cassettes, amplified from these plasmids, were transformed into *A. flagrans* protoplasts. To generate complemented strains for the eight genes, we amplified their individual expression cassettes (each containing ~2 kb promoter, open reading frame (ORF), and ~1 kb terminator) by PCR. We then assembled these fragments, along with a G418 resistance cassette, into the pUC19 vector. After verifying all constructs by sequencing, we transformed them into their respective knockout strains and selected for transformants on PDA plates containing G418.

## S4. Protoplast preparation and transformation

As previously described (Zhang et al. 2025), mycelia cultured in PDB medium (shaken at 28°C, 180 rpm for 24 h) were collected and washed with STC buffer (10 mM Tris-HCl, 1 M sorbitol, 50 mM CaCl_2_). Subsequently, the mycelia were then transferred into 20 mL of cell wall‑lysing enzyme solution and digested on a shaker at 28°C, 90 rpm for 5 h. The protoplasts were collected using centrifugation at 4°C, 3000 × *g* for 8 min. Subsequently, 8 μg of DNA was added to 10^7^ protoplasts. After incubation on ice for 30 min, 1 mL of PTC buffer (10 mM Tris-HCl [pH 7.5], 50 mM CaCl_2_, 50% [wt/vol] polyethylene glycol [PEG] 3350) was added, and the mixture was gently mixed by pipetting, followed by incubation at 28°C for 40 min. After incubation, the entire liquid content of the centrifuge tube was transferred into a 9 cm Petri dish and overlaid with protoplast regeneration medium SSPDA (PDA supplemented with 0.6 M of sucrose, 0.3 g/L peptone, 0.3 g/L tryptone, 0.3 g/L yeast extract). Positive transformants were subsequently selected on PDA medium supplemented with hygromycin B (final concentration 100 μg/mL) or G418 (final concentration 50 μg/mL).

## S5. RNA extraction and RT-qPCR

Total RNA was extracted from WT and mutants using the UNlQ-10 Column Trizol Total RNA Isolation Kit (Sangon Biotech, B511321-0100), followed by first-strand cDNA synthesis with HiScript III 1st Strand cDNA Synthesis Kit (Vazyme, R312-01). RT-qPCR was performed on a Roche LightCycler 480 system (Roche Applied Science, Rotkreuz, Switzerland) using ChamQ SYBR qPCR Master Mix (Vazyme, Q311-02) and gene-specific primers (Table S2). The *gpd* (glyceraldehyde-3-phosphate dehydrogenase) gene was used as the reference, and relative transcript levels were calculated via the 2^–ΔΔCT^ method with three biological replicates (Zhang et al. 2023).

## S6. Chromatin immunoprecipitation (ChIP) analysis

To investigate the transcriptional regulation of downstream genes, ChIP assays were performed using the Magna ChIP^®^ HiSens Chromatin Immunoprecipitation Kit (Sigma-Aldrich, 17-10460). Fungal biomass (~1 g) collected 1 day (24 h) after chlamydospore induction was cross-linked with 1% formaldehyde for 15 min, followed by quenching with 125 mM glycine at 28°C under shaking (100 rpm) for 5 min. Samples were washed twice with 20 mL ice-cold 1× PBS containing 1 mM PMSF and 1 μg/mL protease inhibitor cocktail, immediately frozen in liquid nitrogen, and ground into a fine powder. The powder was resuspended in 1 mL pre-chilled nuclear extraction buffer, and chromatin was fragmented by sonication (30% power, 5 s on/off cycles for 3 min total) to obtain fragments of 100–1000 bp. After centrifugation at 3000 *g* for 20 min at 4°C, the supernatant was collected, with 50 μL retained as the Input control. For immunoprecipitation, 500 μL of supernatant was incubated with 1 μg of Anti-GFP antibody (abcam; ab316291) overnight at 4°C with rotation. Then, 35 μL of Protein A/G Magnetic Beads was added, and the mixture was incubated for an additional 30 min at 4°C to capture antibody-bound chromatin complexes. The beads were washed sequentially with low-salt wash buffer, high-salt wash buffer, and TE buffer. Chromatin was eluted in ChIP elution buffer with agitation at 62°C for 2 h, followed by cross-link reversal at 95°C for 10 min. The DNA was purified using a DNA purification kit, quantified, and subjected to ChIP-qPCR to assess enrichment of target genomic regions. The enrichment level was determined using the 2^−ΔΔCT^ method (Shin et al. 2024).

## S7. RNA-seq analysis

To identify the downstream genes of WC2 and elucidate the molecular mechanism by which WC2 regulates chlamydospore formation in *A. flagrans*, we performed transcriptomic analysis of the Δ*WC2* mutants. Briefly, fresh mycelia from the WT and mutants were harvested after one day of growth in PDB medium. Approximately 10–50 mg of mycelia were evenly spread on water agar (WA) plates supplemented with 2.5 g/L glucose and incubated at 28°C. Chlamydospore development was monitored, and samples were collected at days 0, 1, and 3 post-induction. Three biological replicates were included for each time point. All samples were flash-frozen in liquid nitrogen and stored at –80°C until RNA extraction. The RNA sequencing was performed by Majorbio Bio-Pharm Technology Co., Ltd. (Shanghai, China). Library products were sequenced using an Illumina NovaSeq X Plus. Raw sequencing data were further filtered by fastp (version 0.18.0), and reads were aligned to the reference genome using HISAT2 (version 2.1.0). The differentially expressed genes (DEGs) were identified using DESeq2 (v1.6.3) with the following thresholds: |log_2_(fold change)| ≥ 2 and *p* < 0.05 (Zhang et al. 2023).

## S8. Yeast one-hybrid (Y1H) assay

To investigate the binding interaction between WC2 and *Sre1* promoter region, we performed Y1H assay using the Y187-pHis2 system (Coolaber, YH1011-10T). Briefly, a ~2.0 kb promoter of the *Sre1* gene was cloned into the pHis2 vector to generate pHis2-*Sre1* plasmid. The coding sequence (CDS) of *WC2* was inserted into the pGADT7-Rec2 vector to create pGADT7-Rec2-*WC2* plasmid. Both plasmids were co-transformed into Y187 yeast competent cells. The transformants, along with positive and negative control strains, were then plated on SD/–Leu/–Trp agar plates and incubated at 30°C for 3 days. Fresh colonies (2–3 mm in diameter) were then resuspended in 0.9% NaCl, adjusted to an OD_600_ of 0.2, and serially diluted (50- and 100-fold). Aliquots (5 μL) of the dilutions were spotted onto SD/–Leu/–Trp and SD/–His/–Leu/–Trp plates supplemented with 50 mM 3-amino-1,2,4-triazole (3-AT) to suppress background reporter activity. Protein-DNA interaction was assessed by monitoring yeast growth on the selective plates after a 3-day incubation at 30°C (Liu et al. 2025).

## S9. Interaction network construction

The eight GATA-type transcription factors were submitted to the STRING online platform (https://string-db.org/) for protein-protein interaction network analysis using the "Multiple proteins" module. The minimum required interaction score was determined to be 0.7 (high confidence). To validate selected interactions, we performed yeast two-hybrid (Y2H) assays using the Y2HGold-GAL4 system (Coolaber, YH2011-10T). The coding sequences of *WC2* and *AreA* genes were cloned into the pGADT7 vector (prey, AD), while the coding sequences of *WC1*, *ASD4*, and *Sre1* genes were amplified and cloned into the pGBKT7 vector (bait, BD). Corresponding AD and BD plasmids were co-transformed into Y2HGold strains and cultured on SD/−Leu/−Trp, SD/−Leu/−Trp/−His/−Ade, and SD/−Leu/−Trp/−His/−Ade+X-gal medium at 30°C for 3 days (Zhang et al. 2023).

## S10. Assay of proteolytic activity

To evaluate proteolytic activity, WT, gene knockout, and overexpression strains were individually inoculated into 100 mL of LMZ liquid medium (containing gelatin 20 g/L, peptone 8 g/L, yeast extract 1 g/L, (NH_4_)_2_SO_4_ 0.5 g/L, MgSO_4_ 0.5 g/L, and FeSO_4_ 0.01 g/L) and cultured at 28℃ with shaking at 180 rpm for 7 days. Subsequently, the fermentation supernatants were collected, and proteolytic activity was assessed using the skim milk plate method (Zhang et al. 2023).

## S11. Bimolecular fluorescence complementation (BiFC) assay

The BiFC assay was conducted based on a split-GFP system (Hu et al. 2024). Briefly, GFP was split into the N-terminal (GFP^N^) and the C-terminal (GFP^C^) fragments. The GFP^N^ was fused to the N-terminus of WC1, and the GFP^C^ to the C-terminus of WC2. Each fusion gene, along with its native promoter and terminator, was then cloned into pUC19 vector with the *hph* gene (for the *N^-WC1^* plasmid) or a G418 cassette (for the *C^-WC2^* plasmid) as a selectable marker, yielding the plasmids *N^-WC1^* and *C^-WC2^*, respectively. For the interaction assay, these two plasmids were co-transformed into *A. flagrans* protoplasts. Corresponding control strains were generated by individually transforming the *N^-WC1^* or *C^-WC2^* plasmid into the WT strain.

## S12. GFP fluorescence localization

For the GFP fluorescence localization of the WC1 and WC2, the promoter region (~2.0 kb), the open reading frame (without the stop codon), and the terminator region (~1.0 kb) were amplified from genomic DNA. The green fluorescent protein (GFP) coding sequence was amplified from the plasmid pCT74. For each construct, these fragments, together with the hygromycin resistance gene (*hph*), were assembled into the pUC19 vector to generate the *WC1-EGFP* and *WC2-EGFP* plasmids. Subsequently, the expression cassette was amplified and transformed into *A. flagrans* protoplasts (Kriegler et al. 2025; Zhang et al. 2025). Nuclei were stained with 20 µg/mL 4',6-diamidino-2-phenylindole (DAPI; Sigma-Aldrich, 32670-5MG-F). Meanwhile, cell walls were visualized by staining with 20 µg/mL Calcofluor White (CFW; Sigma-Aldrich, 18909-100ML-F).

References

Hu, X., Hoffmann, D. S., Wang, M., Schuhmacher, L., Stroe, M. C., Schreckenberger, B. et al. 2024. "GprC of the nematode-trapping fungus *Arthrobotrys flagrans* activates mitochondria and reprograms fungal cells for nematode hunting." *Nature Microbiology* 9: 1752-1763.

Kriegler, M., Wernet, V., Hetzer, B., Herrero, S., Wei, A., Wäckerle, J. et al. 2025. "Cell-end marker proteins are required for hyphal ring formation and size determination of traps in *Arthrobotrys flagrans*." *Journal of Cell Science* 138: jcs263744.

Liu, F., Long, S., Hu, J., Qiao, X., Chen, L., Zhou, Y., and Zou, X. 2025. "The CsLOB1-CsERF027 regulatory module positively enhances citrus target spot disease resistance by regulating CsRAP2.3-CsERF1 cascade." *Plant Biotechnology Journal* 23: 5745-5761.

Shin, S., Park, J., Yang, L., Kim, H., Choi, G. J., Lee, Y. W. et al. 2024. "Con7 is a key transcription regulator for conidiogenesis in the plant pathogenic fungus *Fusarium graminearum*." *mSphere* 9: e0081823.

Zhang, Y., Peng, S. Q., He, W. T., Gao, F. F., Shi, Q. F., and Li, G. H. 2025. "FlbD: a regulator of hyphal growth, stress resistance, pathogenicity, and chlamydospore production in the nematode-trapping fungus *Arthrobotrys flagrans*." *Microorganisms* 13: 1847.

Zhang, Y., Wang, X., Ran, Y., Zhang, K. Q., and Li, G. H. 2023. "AfLaeA, a global regulator of mycelial growth, chlamydospore production, pathogenicity, secondary metabolism, and energy metabolism in the nematode-trapping fungus *Arthrobotrys flagrans*." *Microbiology Spectrum* 11: e0018623.
